# Effect of uridine protecting groups on the diastereoselectivity of uridine-derived aldehyde 5’-alkynylation

**DOI:** 10.3762/bjoc.13.153

**Published:** 2017-08-04

**Authors:** Raja Ben Othman, Mickaël J Fer, Laurent Le Corre, Sandrine Calvet-Vitale, Christine Gravier-Pelletier

**Affiliations:** 1 Laboratoire de Chimie et Biochimie Pharmacologiques et Toxicologiques, UMR 8601 CNRS, Université Paris Descartes, Sorbonne Paris Cité (USPC), Centre Interdisciplinaire Chimie Biologie-Paris (CICB-Paris), 45 rue des Saints Pères, 75270 Paris 06, France

**Keywords:** diastereoselective alkynylation, nucleoside, protecting groups, uridine

## Abstract

The 5’-alkynylation of uridine-derived aldehydes is described. The addition of alkynyl Grignard reagents on the carbonyl group is significantly influenced by the 2’,3’-di-*O*-protecting groups (R^1^): *O*-alkyl groups led to modest diastereoselectivities (65:35) in favor of the 5’*R*-isomer, whereas *O*-silyl groups promoted higher diastereoselectivities (up to 99:1) in favor of the 5’*S*-isomer. A study related to this protecting group effect on the diastereoselectivity is reported.

## Introduction

**Figure 1:**
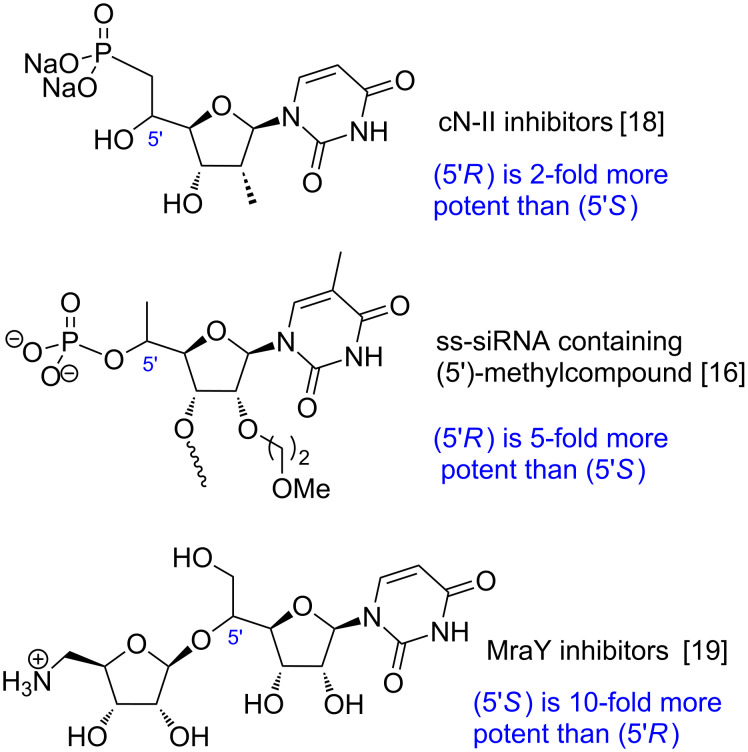
C-5’ configuration of nucleoside derivatives and related biological activity.

Nucleoside and nucleotide derivatives or analogues are biologically active compounds of major interest [1–2]. Their widespread applications span from therapeutic agents, such as antibacterial [3–5], antiviral [6] or antitumor [7–8] drugs, to epigenetic modulators [9] or chemical tools [10–11]. Furthermore, they represent central monomers for oligonucleos(t)ide synthesis [12–17]. In the past decades, chemical modifications of these crucial building blocks have been extensively studied, both on the nucleobase itself and on the sugar moiety [6]. Many of them concern the C-2’ and C-3’ sugar positions. However, functionalization at C-5’ has been much less reported, particularly the stereocontrolled formation of a stereogenic center at C-5’, in spite of its important role for biological activity (Figure 1) [16–19].

In numerous approaches, this 5′ stereogenic center comes from the chiral pool and the nucleobase is introduced under Vorbrüggen conditions [20–26]. It can also be created directly on the nucleoside, either by functionalization of an alkene at C-5’ [27–32] or by the diastereoselective addition of a nucleophile on a carbonyl group. The latter can involve either the reduction of a ketone at C-5’ [12,33–35], or the addition on an aldehyde of various nucleophiles such as enolates [15,36–38], allylborane [39], dialkyl phosphites [40], TMSCN [41] or Grignard reagents [12,17,35,42–47]. Aside from the use of chiral ligands promoting an excellent facial discrimination of the aldehyde [34], the addition of Grignard reagents usually proceed with moderate diastereoselectivity and yield (Table 1) [12,35,43–46,48].

**Table 1:** Reported C-5’ diastereoselectivity for the addition of an organometallic reagent on nucleoside aldehydes.

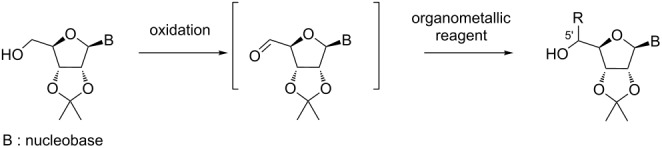								

Entry	Base^a^	Organometallic reagent	Solvent	Temp (°C)	dr	C-5’^b^	Yield (%)^c^	Ref.

1	A(Bz)	CH_3_MgBr	THF	−78	3:2	*R*	n.d.	[35]
2	U	AllylMgBr	THF	100	16:1	*R*	n.d.	[42]
3	A	TMSC≡CMgBr	THF	−20	2:1	*R*	44^d^	[43]
4	U	TMSC≡CMgBr	THF	−15	2:1	*R*	21	[12]
5	U	TESC≡CMgBr	THF	−15	2:1	*R*	42	[12]
6	A(Bz)	TMSC≡CMgBr	THF	−15	2:1	*R*	40	[44]
7	U	AllylMgBr	THF	−78	5:1	*R*	n.d.	[45]
8	U	CH_2_=CHMgBr	THF	−78	5:1	*R*	n.d.	[46]
9	U(MTPM)^e^	4-phenyl-1-butyne, iPrMgCl, Zn(OTf_2_)	toluene	rt	1:1.7	*S*	47	[48]

^a^The nature of the nucleobase protecting group is mentioned in brackets; ^b^C-5’ configuration of the major diastereomer; ^c^isolated yield of the major diastereomer; ^d^contaminated with a small amount of the other isomer; ^e^MTPM: monomethoxytetrachlorodiphenylmethoxymethyl.

In the course of our program devoted to the synthesis of new MraY inhibitors [49–50], we were interested in developing a more efficient access to 5’-ethynyluridine, a crucial building block for the further synthesis of triazole-containing compounds [49]. Intrigued by the moderate diastereomeric ratio reported for the addition or organometallic reagents onto nucleoside aldehyde (Table 1), we decided to investigate the influence of the protecting groups of the uridine aldehyde on the stereochemical outcome of the nucleophilic addition of a Grignard reagent and we wish to report herein the results of our study.

## Results and Discussion

**Scheme 1:**
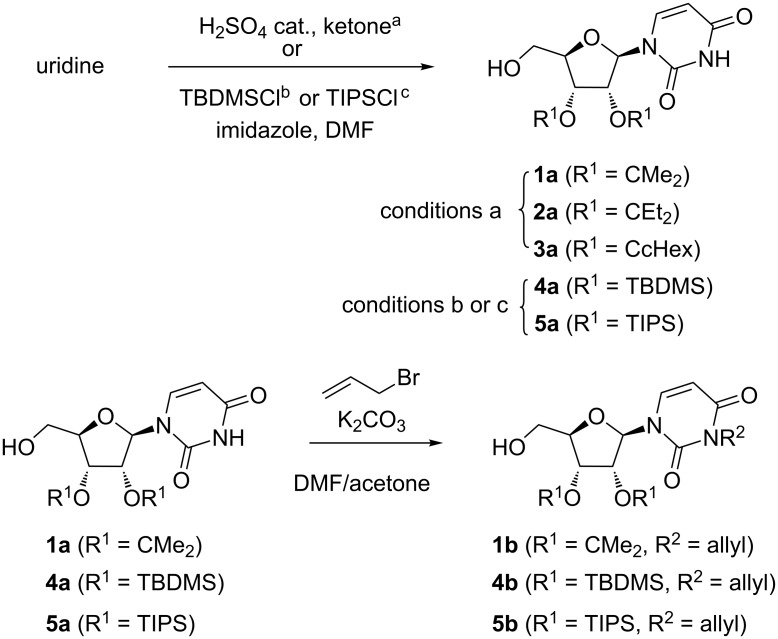
Synthesis of alcohols **1–5**.

We first prepared primary alcohols **1**–**5** from uridine and differing by the nature of R^1^ protecting groups. The secondary alcohols at C-2’ and C-3’ were protected either as a cyclic ketal (isopropylidene (**1a**) [12], isopentylidene (**2a**), cyclohexylidene (**3a**)) or as acyclic silyl ethers (**4a** [15], R^1^ = TBDMS and **5a** [51], R^1^ = TIPS). Some compounds were also N3-allylated (**1b**, **4b** and **5b**) to evaluate the possible influence of R^2^ on the diastereoselectivity of the nucleophilic addition (Scheme 1).

**Scheme 2:**
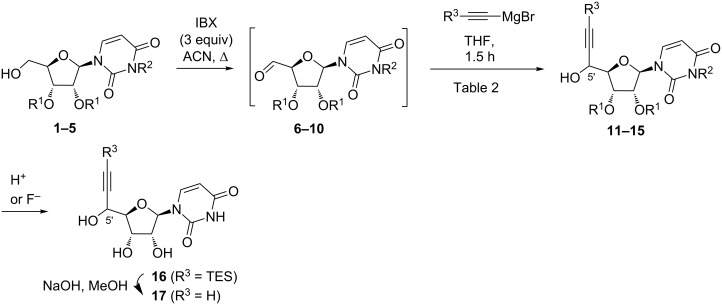
Synthesis of propargylic alcohols **11–15** and their partial or complete deprotection.

Primary alcohols **1–5** were submitted to an oxidation/Grignard addition sequence leading to the corresponding propargyl alcohols **11**–**15** (Scheme 2). The aldehydes **6–10** resulting from oxidation with IBX were isolated and directly submitted to Grignard addition without further purification. Grignard reagents were prepared from trimethylsilyl-, triethylsilyl- or triisopropylsilylacetylene and ethylmagnesium bromide in THF at 0 °C, in order to vary the steric hindrance of the silyl group (R^3^). To unambiguously determine the ratio of diastereomers and the C-5’ configuration of the major one for the synthesized propargylic alcohols **11**–**15**, their partial or complete deprotection was carried out to take advantage of an unequivocal ^1^H NMR comparison with known compounds, or reliable derivatives.

**Scheme 3:**
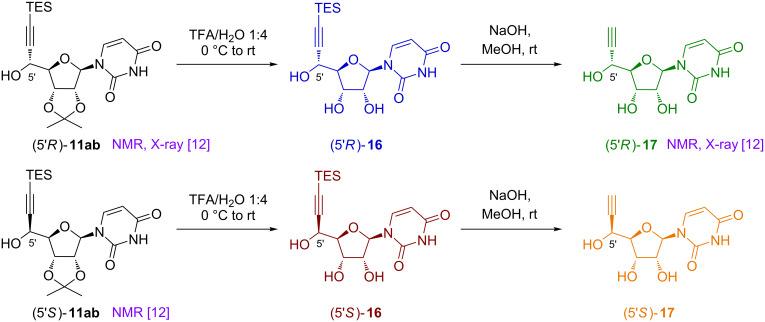
Synthesis of reference compounds and strategy for assignment of C-5’ configuration.

Thus, we prepared compounds (5’*R*)-**11ab** [12] and (5’*S*)-**11ab** [12] from protected uridine **1a** and triethylsilylethynylmagnesium chloride. Indeed, Vasella’s group devoted a huge amount of work to the synthesis and characterization of these compounds and notably reported that TES was better than TMS for efficient separation of both isomers, resulting in an improved 42% yield for the isolated major diastereomer (5’*R*)**-11ab** [12]. Then, subsequent acidic hydrolysis afforded (5’*R*)-**16** and (5’*S*)-**16** and alkyne desilylation under basic conditions provided (5’*S*)-**17** and (5’*R*)-**17** [12] (Scheme 3).

**Figure 2:**
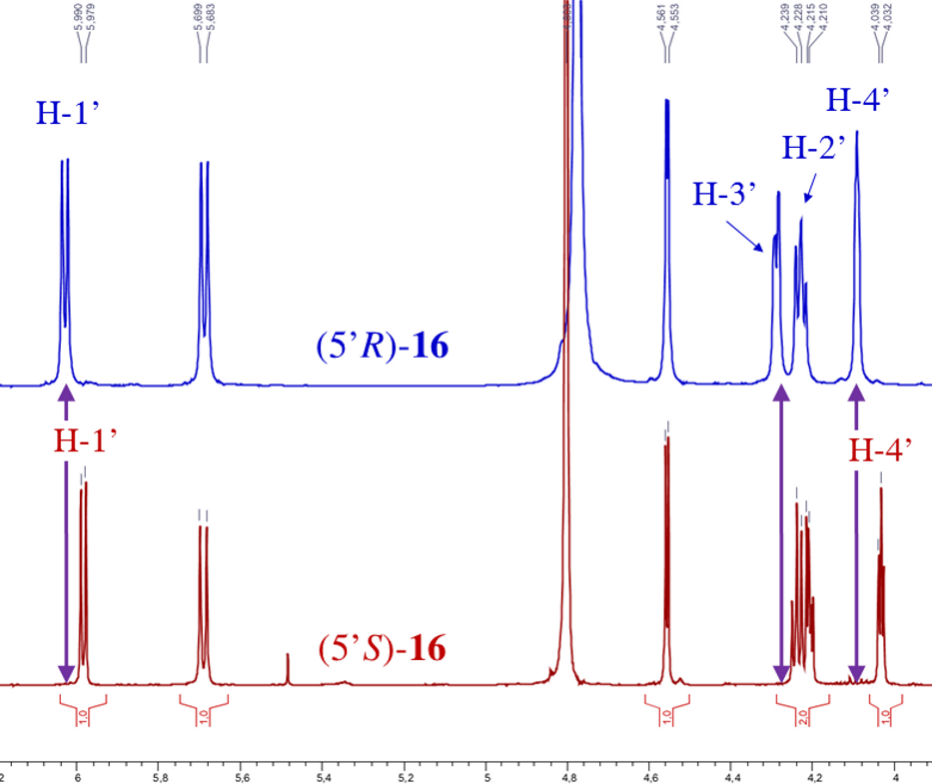
^1^H NMR of (5’*R*)-**16** and (5’*S*)-**16** and of a (5’*R*)/(5’*S*)-**16** mixture.

**Figure 3:**
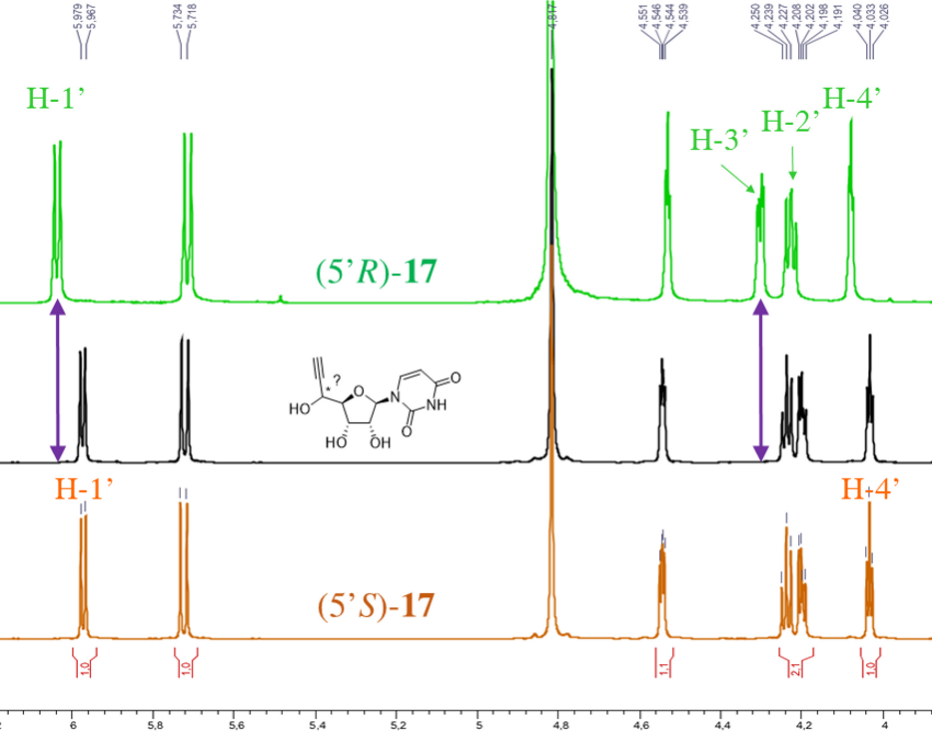
^1^H NMR of (5’*R*)-**17** and (5’*S*)-**17** and example of configuration determination for a pure isolated compound with unknown configuration.

The differences between ^1^H NMR data of the related diastereomeric compounds (Figure 2 and Figure 3) provided a conclusive evidence to unequivocally attribute the absolute configuration at C-5’ of the synthesized compounds. Thus, for compounds **11–15** with R^3^ = TES, the configuration at C-5’ was determined according to the ^1^H NMR spectra of (5’*R*) or (5’*S*)-**16** derivative (Figure 2). Indeed, in ^1^H NMR spectrum (500 MHz, CDCl_3_), H-1’ appears as a doublet at 6.02 ppm for (5’*R*)-**16** (*^3^J*_H-1’-H-2’_ = 7.0 Hz) and as a doublet at 5.98 ppm for (5’*S*)-**16** (*^3^J*_H-1’-H-2’_ = 6.0 Hz). Furthermore, H-4’ appears as a multiplet at 4.11–4.09 ppm for (5’*R*)-**16** and as a triplet at 4.05 ppm for (5’*S*)-**16** (*^3^J*_H-4’-H-3’_ = *^3^J*_H-4’-H-5’_ = 3.0 Hz). For compounds **11–15** with R^3^ ≠ TES, the configuration at C-5’ was attributed after complete deprotection leading to compound (5’*R*) or (5’*S*)-**17** (Figure 3). As observed for compound **16**, the H-1’ chemical shift is more shielded for (5’*S*)-**17** isomer (doublet (*^3^J*_H-1’-H-2’_ = 6.0 Hz) at 5.98 ppm) than for the (5’*R*)-**17** diastereomer (doublet (*^3^J*_H-1’-H-2’_ = 7.0 Hz) at 6.04 ppm). Similarly, the H-4’ chemical shift is more shielded for (5’*S*)-**17** isomer (triplet (*^3^J*_H-4’-H-3’_ = *^3^J*_H-4’-H-5’_ = 3.5 Hz) at 4.03 ppm) than for the (5’*R*)-**17** diastereomer (triplet (*^3^J*_H-4’-H-3’_ = *^3^J*_H-4’-H-5’_ = 2.5 Hz) at 4.07 ppm). Moreover, chemical shifts for H-3’ (doublet of doublet (^3^*J*_H3’-H2’_ = 5.5 Hz, ^3^*J*_H3’-H4’_ = 2.5 Hz) at 4.30 ppm) and H-2’ (doublet of doublet (^3^*J*_H2’-H1’_ = 7.0 Hz, ^3^*J*_H2’-H3’_ = 5.5 Hz) at 4.22 ppm) for the (5’*R*)-**17** isomer are also significantly different from that of the (5’*S*)-**17** isomer (triplet (^3^*J*_H2’-H1’_ = ^3^*J*_H2’-H3’_ = 6.0 Hz) at 4.23 ppm for H-2’ and doublet of doublet (^3^*J*_H3’-H2’_ = 6.0 Hz, ^3^*J*_H3’-H4’_ = 3.5 Hz) at 4.20 ppm for H-3’.

The results and conditions of assays involving the various synthesized substrates **1–5** and Grignard reagents are reported in Table 2. The diastereomeric ratio of **11–15** was determined by ^1^H NMR or HPLC analysis of the crude mixture and the C-5’ configuration of the major diastereomer was assigned as explained above. To evaluate the effect of the temperature on the diastereomeric ratio (Table 2, entries 1 and 2), we first tried to carry out the reaction at lower temperature but it remained close to 2:1 in favor of the (5’*R*)-**11ab**, either with conventional or inverse order of addition. We also tried to increase the reaction temperature [52] but, even at 0 °C, the components in the reaction mixture were largely degraded. The protection of uracil nitrogen at N-3 position by an allyl group did not significantly modify the 5’*R*/5’*S* ratio (Table 2, entry 3). Increasing the bulkiness of the ketal protecting group by introducing an isopentylidene **2a** (Table 2, entry 4) or a cyclohexylidene **3a** (Table 2, entry 5) did not modify the observed diastereoselectivity.

**Table 2:** Influence of the protecting groups and conditions on the diastereoselectivity of the alkynyl Grignard addition on uridine derived aldehydes.

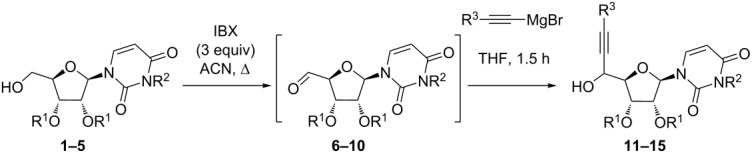										

Entry	Alcohol			Aldehyde^a^	Grignard reagent		Temperature (°C)	Propargyl alcohol		
	**1–5**	R^1^	R^2^	**6–10**	R^3^	equiv		**11–15**	5’*R*/5’*S* ratio^b^	Yield (%)^c^

1	**1a**	CMe_2_	H	**6a**	TES	2.5	−50	**11ab**	65:35^d^	conv: 65%
2	**1a**	CMe_2_	H	**6a**	TES	2.5	−78	**11ab**	65:35^d^	conv: 65%
3	**1b**	CMe_2_	allyl	**6b**	TES	2.5	−15	**11bb**	65:35^e^	52 (24)
4	**2a**	CEt_2_	H	**7a**	TES	2.5	−15	**12ab**	65:35	54 (15)
5	**3a**	C*c*Hex	H	**8a**	TES	2.5	−15	**13ab**	70:30	65 (18)
6	**4a**	TBDMS	H	**9a**	TES	2.5	−15	**14ab**	15:85	64 (48)
7	**4a**	TBDMS	H	**9a**	TES	3.5	−78	**14ab**	15:85	51 (38)
8	**4b**	TBDMS	allyl	**9b**	TES	3.5	−78	**14bb**	9:91^e^	59 (39)
9	**5a**	TIPS	H	**10a**	TES	5	−15	**15ab**	5:95	64 (53)
10	**5a**	TIPS	H	**10a**	TES	5	−78	**15ab**	5:95	65 (32)
11	**5a**	TIPS	H	**10a**	TMS	5	−78	**15aa**	10:90	66 (36)
12	**5a**	TIPS	H	**10a**	TIPS	5	−78	**15ac**	1:99	61
13	**5a**	TIPS	H	**10a**	TIPS	5	−15	**15ac**	2:98	55
14	**5a**	TIPS	H	**10a**	TIPS	2.5	−15	**15ac**	2:98	conv: 85%
15	**5b**	TIPS	allyl	**10b**	TIPS	5	−78	**15bc**	10:90^e^	43 (27)

^a^Normal addition procedure; ^b^determined by ^1^H NMR and/or HPLC of the crude mixture; ^c^yield of the mixture of diastereomers over two steps from the corresponding primary alcohol, isolated yield of the major diastereomer is shown in brackets; ^d^an “inverse” order of addition led to the same diastereomeric ratio; ^e^5’-configuration of the major diastereomer was not determined.

We next turned to the use of acyclic protecting groups. *tert*-Butyldimethylsilyl ether **4a** (Table 2, entry 6) led to an improved 85:15 ratio. Furthermore, we were delighted to discover that, contrary to that which was observed with ketal groups, the major diastereomer **4a** obtained using *tert*-butyldimethylsilyl ether displayed the 5’*S* configuration. As was noted for isopropylidene as a protecting group, decreasing the temperature (Table 2, entry 7) or protecting the N-3 nitrogen of uracil (Table 2, entry 8) did not significantly change the 5’*R*/5’*S* ratio. In both cases, using 3.5 equivalents of Grignard reagent was required to complete the reaction.

In order to improve this reverse diastereoselectivity, we envisaged the use of a more bulky protecting group, such as the triisopropylsilyl group. The addition of triethylsilylacetylide magnesium bromide gave a 5’*R* /5’*S* ratio of 5:95 (Table 2, entries 9 and 10). To determine if R^3^ was able to influence this ratio, we also tested trimethylsilylacetylmagnesium bromide (Table 2, entry 11) and triisopropylsilylacetylmagnesium bromide (Table 2, entry 12). When R^3^ = TMS, the 5’*R*/5’*S* ratio was only 10:90, whereas for R^3^ = TIPS, an excellent 5’*R*/5’*S* 1:99 ratio was reached, allowing the direct synthesis of (5’*S*)-**15ac** with a substantial 61% isolated yield over two steps. Running this reaction at −15 °C (Table 2, entry 13) slightly diminished the yield to 55%. The bulkiness of the TIPS groups required the use of 5 equivalents of Grignard reagent to get a clean reaction and complete conversion of the starting material (Table 2, entry 14). The protection of the N-3 nitrogen with an allyl group was unfavorable and led to a 5’*R*/5’*S* 10:90 ratio (Table 2, entry 15). The three attempts of C-5’ alkynylation of N-3-allylated uridine aldehydes, did not revealed marked influence on the diastereoselective ratio (Table 2, entries 3, 8, 15).

**Figure 4:**
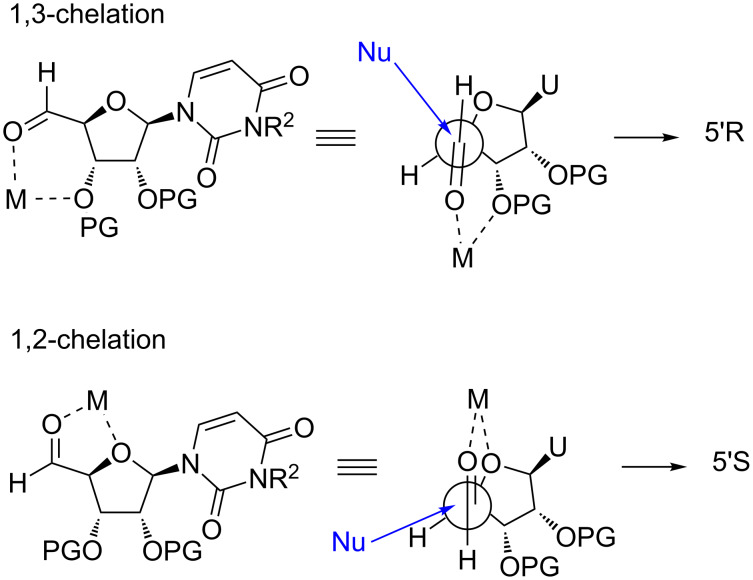
Hypothetical Cram chelated models.

To explain the diastereoselective outcome of the reaction, we first considered Cram chelated models (Figure 4).

A 1,3-chelation of the metal with the carbonyl group and the protecting group oxygen atom at C-3’ would promote an attack of the nucleophile on the *Re* face of the aldehyde and thus the formation of the 5’*R* diastereoisomers, whereas a 1,2-metal chelation with the carbonyl group and the endocyclic oxygen atom of uridine at C-4’ would explain the formation of the 5’*S* diastereoisomer (attack of the nucleophile on the *Si* face of the aldehyde, Figure 4). In such a case, the poor chelating ability of silyl ether groups, along with their bulkiness, would strongly disfavor the 1,3-chelate and the 5’*R*-isomer formation, justifying the high selectivity observed for the silyl protected compounds in favor of the 5’*S*-isomer. However, when ketal groups are used, the 5’*R*-isomer is predominantly formed with a modest diastereoselectivity, meaning that the most stable transition state would involve a 1,3-metal chelation with the carbonyl group and the protecting group oxygen atom at C-3’ of uridine. Even if the later 1,3-chelation can be envisioned, we found this hypothesis very unlikely due to the large distance between the two oxygen atoms.

**Figure 5:**
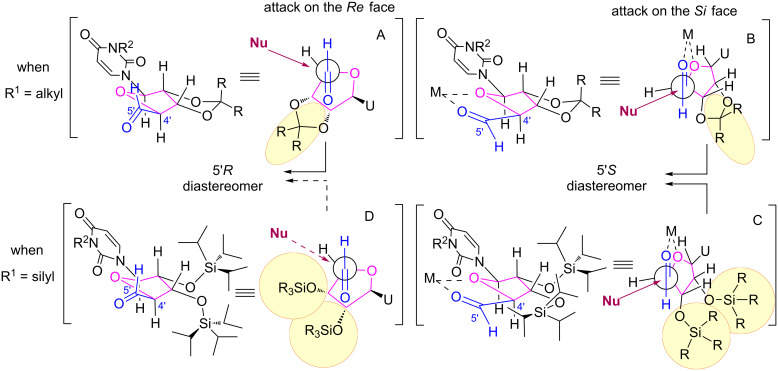
Proposed stereochemical models.

Therefore, the following models would be more consistent with our results (Figure 5).

When R^1^ is a ketal group (R^1^ = alkyl, Figure 5 top), on the one hand, the major formation of the 5’*R*-isomer would be explained by the Felkin Anh model (Figure 5A top, left) with an attack of the nucleophile on the *Re* face of the aldehyde. On the other hand, the minor formation of the 5’*S* diastereomer would result from an attack of the nucleophile on the *Si* face of the aldehyde on the 1,2-chelated model (Figure 5B top, right).

When R^1^ is a trialkylsilyl protecting group (R^1^ = silyl, Figure 5 bottom), on the one hand, the 5’*S* diastereomer formation is in agreement with an attack on the least hindered face of a 1,2-chelated conformer (Figure 5C bottom, right). On the other hand, the minute formation of the 5’*R*-isomer that would be explained by the Felkin–Ahn model (Figure 5D bottom, left) is strongly disfavored due to the the bulkiness of the silyl groups hampering an approach of the nucleophile on the *Re* face: the bulkier the silyl group, the stronger the effect.

## Conclusion

In summary, we report a study on the diastereoselective 5’-alkynylation of uridine aldehydes displaying various protecting groups on the secondary alcohols at C-2’ and C-3’ and at the N-3 position of the uracile. Our results show that while the N-3 protection has little influence on the alkynylation diastereoselectivity, the nature of the diol protecting group strongly impact the diastereoselective outcome of the reaction. Indeed, whatever the ketal group used, the major 5’*R*-isomer is obtained with a 2:1 ratio whereas the protection as silyl ethers leads to an inverse diastereoselectivity in favor of the 5’*S*-isomer. We propose stereochemical models to rationalize the observed diastereoselectivity. Furthermore by increasing the bulkiness of the silyl group both on the diol and the Grignard reagent, we manage to obtain an excellent diastereoselectivity. Indeed, by using the most bulky 2’,3’-*O*-TIPS protecting groups and TIPS-ethynylmagnesium bromide, the 5’-ethynylation was achieved in a 99:1 ratio in favor of the 5’*S-*isomer. The resulting building block with a broad potential in nucleos(t)ide derivative syntheses is obtained in a very satisfactory 61% yield over two steps.

## Experimental

**General procedure for N3-allylation of protected uridine derivatives.** To a solution of protected uridine derivative (1 equiv) in DMF/acetone (1:1, final concentration 0.4 M) was added K_2_CO_3_ (1.8 equiv) and allyl bromide (1.5 equiv). The suspension was stirred at 50 °C for 12 h, filtered and concentrated in vacuo. The resulting residue was submitted to flash chromatography (elution conditions mentioned below) to afford the corresponding *N*-allyl compound.

**General procedure for the oxidation of uridine derivatives 1–5.** To a suspension of protected uridine derivative **1–5** (1 equiv) in acetonitrile (5 × 10^−2^ M) was added IBX (3 equiv). The suspension was refluxed for 45 min–1.5 h until complete conversion of starting material (TLC). The suspension was cooled to rt, filtered on a celite^®^ pad and the cake was washed with EtOAc. The filtrate was then concentrated in vacuo to afford crude aldehydes **6–10** which were used without other purification (quantitative yield). Aldehydes have been characterized by ^1^H and ^13^C NMR. Since they are quite unstable, crude aldehydes were used without any further purification and directly engaged in the alkynylation reaction.

**General procedure for the 5'-alkynylation of uridine-derived aldehydes 6–10.** At 0 °C, under Ar, to a solution of trialkylsilylacetylene (1.5–2.45 equiv), in THF (0.25 M) was added dropwise a solution of ethylmagnesium bromide (3 M in Et_2_O, 1.5–2.45 equiv). The solution was stirred at 0 °C for 10 min and then at rt for 1 h. Crude aldehydes **6–10** were dissolved in THF (0.1 M), transferred into a dropping funnel and slowly added to the solution of the Grignard reagent at −15 °C. The mixture was stirred at −15 °C for 1 h 30 and was allowed to slowly warm to rt for 24 h. The reaction was then quenched by addition of a saturated aqueous solution of NH_4_Cl (40 mL) and THF was removed in vacuo. The aqueous phase was extracted with EtOAc and the combined organic layers were dried over Na_2_SO_4_, filtered and concentrated in vacuo. The crude foam was purified by flash chromatography.

## Supporting Information

File 1 Description of the materials and methods, and the preparation and characterization of new compounds.

File 2 Copies of spectra for final compounds and NMR studies.
