# Supplementary material for: Effect of uridine protecting groups on the diastereoselectivity of uridine-derived aldehyde 5’-alkynylation
Source: Beilstein J Org Chem. 2017 Aug 4;13:1533–41. doi: 10.3762/bjoc.13.153 (PMC5550804; doi:10.3762/bjoc.13.153)
Supplement: File 1 — Description of the materials and methods, and the preparation and characterization of new compounds. [file Beilstein_J_Org_Chem-13-1533-s001.pdf]

Supporting Information

for

Effect of uridine protecting groups on the diastereoselectivity of uridine-derived aldehyde 5’-alkynylation

Raja Ben Othman[‡], Mickaël J. Fer[‡], Laurent Le Corre, Sandrine Calvet-Vitale* and Christine Gravier-Pelletier*

Address: Laboratoire de Chimie et Biochimie Pharmacologiques et Toxicologiques, UMR 8601 CNRS, Université Paris Descartes, Sorbonne Paris Cité (USPC), Centre Interdisciplinaire Chimie Biologie-Paris (CICB-Paris), 45 rue des Saints Pères, 75270 Paris 06, France.

Email: Sandrine Calvet-Vitale - sandrine.calvet-vitale@parisdescartes.fr; Christine Gravier-Pelletier - christine.gravier-pelletier@parisdescartes.fr

*Corresponding author

[‡]Equal contributors

Description of materials and methods,
and preparation and characterization of new compounds

N^3-Allyl-2′,3′-*O*-isopropylideneuridine (1b). Compound **1b** was synthesized according to the general procedure for *N*-allylation of protected uridine derivatives from acetonide **1a**[1] (1.0 g, 3.52 mmol, 1 equiv). Flash chromatography of the residue (cyclohexane/EtOAc 1:1) afforded **1b** as a gum (981 mg, 86% yield): R_f 0.34 (cyclohexane/EtOAc 1/1); $[\alpha]_D$ - 28 (*c* 0.5, CH_2Cl_2); IR (film) 3607*br*, 2297*m*, 1700*s*, 1676*s*, 1453*m*, 1040*m*; ^{1}H NMR δ 7.21 (d, 1H, $J_{H6\text{-}H5}$ = 8.5 Hz, H_6), 5.87 (ddt, 1H, $J_{H8\text{-}H9a}$ = 17.0 Hz, $J_{H8\text{-}H9b}$ = 10.0 Hz, $J_{H8\text{-}H7a}$ = $J_{H8\text{-}H7b}$ = 6.0 Hz, H_8), 5.77 (d, 1H, $J_{H5\text{-}H6}$ = 8.5 Hz, H_5), 5.54 (d, 1H, $J_{H1'\text{-}H2'}$ = 3.0 Hz, $H_{1'}$), 5.29-5.24 (m, 1H, H_{9a}), 5.22-5.18 (m, 1H, H_{9b}), 5.07 (dd, 1H, $J_{H2'\text{-}H3'}$ = 6.5 Hz, $J_{H2'\text{-}H1'}$ = 3.0 Hz, $H_{2'}$), 4.99 (dd, 1H, $J_{H3'\text{-}H2'}$ = 6.5 Hz, $J_{H3'\text{-}H4'}$ = 3.5 Hz, $H_{3'}$), 4.54-4.52 (m, 2H, H_7), 4.31-4.29 (m, 1H, $H_{4'}$), 3.93 (dt, 1H, $J_{H5'a\text{-}H5'b}$ = 12.0 Hz, $J_{H5'a\text{-}H4'}$ = $J_{H5'a\text{-}OH}$ = 3.0 Hz, $H_{5'a}$), 3.81 (ddd, 1H, $J_{H5'b\text{-}H5'a}$ = 12.0 Hz, $J_{H5'b\text{-}H4'}$ = 3.0 Hz, $J_{H5'b\text{-}OH}$ = 7.5 Hz, $H_{5'b}$), 2.70 (dd, 1H, $J_{OH\text{-}H5'a}$ = 3.0 Hz, $J_{OH\text{-}H5'b}$ = 7.5 Hz, OH), 1.58 (s, 3H, $H_{7'}$), 1.37 (s, 3H, $H_{7'}$); ^{13}C NMR *δ* 162.2 (C_4), 150.9 (C_2), 140.9 (C_6), 131.3 (C_8), 118.5 (C_9), 114.5 ($C_{6'}$), 102.4 (C_5), 97.4 ($C_{1'}$), 87.1 ($C_{4'}$), 83.8 ($C_{2'}$), 80.5 ($C_{3'}$), 62.9 ($C_{5'}$), 43.2 (C_7), 27.5, 25.4 ($C_{7'}$); HRMS ESI$^+$ calcd for $C_{15}H_{21}N_2O_6^+$ (M + H)$^+$ 325.1394, found 325.1388.

2′,3′-*O*-Isopentylideneuridine (2a). To a suspension of uridine (1.0 g, 4.09 mmol, 1 equiv) in pentanone (21.5 mL, 203.1 mmol, 49.6 equiv), was added sulfuric acid (300 µL, 4.09 mmol, 1 equiv) and anhydrous $MgSO_4$ (985 mg, 8.19 mmol, 2 equiv). The suspension was stirred at rt for 48 h, neutralized by a dropwise addition of triethylamine and concentrated in vacuo. Flash chromatography of the resulting orange oil (cyclohexane/EtOAc 1:9 to 0:10) afforded the isopentylidene **2a** as a white foam (1.0 g, 78% yield): R_f 0.35 (Cyclohexane/EtOAc 1/9); mp 112-115 °C; $[\alpha]_D$ - 35 (*c* 1.0, CH_2Cl_2); IR (film) 2999*br*, 1691*s*, 1463*m*, 1384*w*, 1265*m*, 1087*m*; ^{1}H NMR δ 9.30 (br s, 1H, NH), 7.36 (d, 1H, $J_{H6\text{-}H5}$ = 8.5 Hz, H_6), 5.73 (dd, 1H, $J_{H5\text{-}H6}$ = 8.5 Hz, $J_{H5\text{-}NH}$ = 1.5 Hz, H_5), 5.60 (d, 1H, $J_{H1'\text{-}H2'}$ = 3.0 Hz, $H_{1'}$), 5.06 (dd, 1H, $J_{H2'\text{-}H3'}$ = 7.0 Hz, $J_{H2'\text{-}H1'}$ = 3.0 Hz, $H_{2'}$), 4.97 (dd, 1H, $J_{H3'\text{-}H2'}$ = 7.0 Hz, $J_{H3'\text{-}H4'}$ = 4.0 Hz, $H_{3'}$), 4.32-4.30 (m, 1H, $H_{4'}$), 3.92 (dt, 1H, $J_{H5'a\text{-}H5'b}$ = 12.0 Hz, $J_{H5'a\text{-}H4'}$ = $J_{H5'a\text{-}OH}$ = 4.0 Hz, $H_{5'a}$), 3.85-3.80 (m, 1H, $H_{5'b}$), 2.99 (dd, 1H, $J_{OH\text{-}H5'a}$ = 4.0 Hz, $J_{OH\text{-}H5'b}$ = 7.0 Hz, OH), 1.80 (q, 2H, $J_{H7'\text{-}H8'}$ = 7.5 Hz, $H_{7'}$), 1.63 (q, 2H, $J_{H7'\text{-}H8'}$ = 7.5 Hz, $H_{7'}$), 0.99 (t, 3H, $J_{H8'\text{-}H7'}$ = 7.5 Hz, $H_{8'}$), 0.89 (t, 3H, $J_{H8'\text{-}H7'}$ = 7.5 Hz, $H_{8'}$); ^{13}C NMR *δ* 163.2 (C_4), 150.4 (C_2), 143.1 (C_6), 119.1 ($C_{6'}$), 102.8 (C_5), 96.3 ($C_{1'}$), 87.3 ($C_{4'}$), 84.1 ($C_{2'}$), 80.6 ($C_{3'}$), 62.9 ($C_{5'}$), 29.5, 29.4 ($C_{7'}$), 8.5, 7.9 ($C_{8'}$); HRMS ESI$^-$ calcd for $C_{14}H_{19}N_2O_6^-$ (M - H)$^-$ 311.1249, found 311.1252.

2′,3′-*O*-Cyclohexylideneuridine (3a). To a solution of uridine (1.0 g, 4.09 mmol, 1 equiv) in cyclohexanone (6 mL, 57.9 mmol, 14.1 equiv) was added sulfuric acid (cat). The suspension was stirred at 50 °C for 12 h, cooled to rt, neutralized by a dropwise addition of triethylamine and concentrated *in vacuo*. Flash chromatography of the resulting orange oil (Cyclohexane/EtOAc 6/4 to

[1] Eppacher, S.; Solladie, N.; Bernet, B.; Vasella, A. *Helv. Chim. Acta* **2000**, *83*, 1311−1330.

3/7) afforded the cyclohexylidene **3a** as a white foam (1.18 g, 89% yield): R_f 0.22 (Cyclohexane/EtOAc 3/7); mp 136-140 °C; $[\alpha]_D$ - 36 (*c* 1.0, CH_2Cl_2); IR (film) 2917*br*, 1691*s*, 1463*m*, 1388*w*; ^{1}H NMR δ 9.22 (br s, 1H, NH), 7.39 (d, 1H, J_{H6-H5} = 8.0 Hz, H_6), 5.74 (d, 1H, J_{H5-H6} = 8.0 Hz, H_5), 5.60 (d, 1H, $J_{H1'-H2'}$ = 3.0 Hz, $H_{1'}$), 5.04 (dd, 1H, $J_{H2'-H3'}$ = 6.5 Hz, $J_{H2'-H1'}$ = 3.0 Hz, $H_{2'}$), 4.94 (dd, 1H, $J_{H3'-H2'}$ = 6.5 Hz, $J_{H3'-H4'}$ = 3.5 Hz, $H_{3'}$), 4.30-4.28 (m, 1H, $H_{4'}$), 3.92 (dt, 1H, $J_{H5'a-H5'b}$ = 12.0 Hz, $J_{H5'a-H4'}$ = $J_{H5'a-OH}$ = 3.0 Hz, $H_{5'a}$), 3.84-3.79 (m, 1H, $H_{5'b}$), 2.99 (dd, 1H, $J_{OH-H5'a}$ = 3.0 Hz, $J_{OH-H5'b}$ = 7.5 Hz, OH), 1.79-1.76 (m, 2H, $H_{8'a}$), 1.69-1.64 (m, 2H, $H_{8'b}$), 1.59-1.54 (m, 4H, $H_{7'}$), 1.43-1.39 (m, 2H, $H_{9'}$); ^{13}C NMR *δ* 163.3 (C_4), 150.5 (C_2), 143.2 (C_6), 115.4 ($C_{6'}$), 102.8 (C_5), 96.2 ($C_{1'}$), 87.2 ($C_{4'}$), 83.3 ($C_{2'}$), 80.1 ($C_{3'}$), 62.8 ($C_{5'}$), 37.3, 34.9 ($C_{7'}$), 25.0, 24.1 ($C_{8'}$), 23.7 ($C_{9'}$); HRMS ESI$^+$ calcd for $C_{15}H_{21}N_2O_6^+$ $(M + H)^+$ 325.1394, found 325.1391.

N^3-Allyl-2′,3′-*O*-(*tert*-butyldimethylsilyl)uridine (4b)

Compound **4b** was synthesized according to the general procedure for *N*-allylation of protected uridine derivatives from compound **4a**[2] (400 mg, 0.85 mmol, 1 equiv). Flash chromatography of the residue (cyclohexane/EtOAc 8:2) afforded **4b** as a white solid (404 mg, 93% yield): R_f 0.25 (cyclohexane/EtOAc 8:2); mp 201-207 °C; $[\alpha]_D$ - 21 (*c* 1.0, CH_2Cl_2); IR (film) 3710*br*, 3005*br*, 2369*w*, 1708*m*, 1664*s*, 1461*m*, 940*m*; ^{1}H NMR δ 7.51 (d, 1H, J_{H6-H5} = 8.0 Hz, H_6), 5.87 (ddt, 1H, J_{H8-H9a} = 17.0 Hz, J_{H8-H9b} = 10.0 Hz, J_{H8-H7a} = J_{H8-H7b} = 6.0 Hz, H_8), 5.76 (d, 1H, J_{H5-H6} = 8.0 Hz, H_5), 5.48 (d, 1H, $J_{H1'-H2'}$ = 6.0 Hz, $H_{1'}$), 5.25 (d, 1H, J_{H9a-H8} = 17.0 Hz, $J_{H9a-H7a}$ = $J_{H9a-H7b}$ = 2.0 Hz, H_{9a}), 5.17 (dt, 1H, J_{H9b-H8} = 10.0 Hz, $J_{H9a-H7a}$ = $J_{H9a-H7b}$ = 2.0 Hz, H_{9b}), 4.61 (t, 1H, $J_{H2'-H3'}$ = 6.0 Hz, $J_{H2'-H1'}$ = 6.0 Hz, $H_{2'}$), 4.55-4.42 (m, 2H, H_7), 4.18 (dd, 1H, $J_{H3'-H2'}$ = 6.0 Hz, $J_{H3'-H4'}$ = 3.0 Hz, $H_{3'}$), 4.09-4.06 (m, 1H, $H_{4'}$), 3.89 (dt, 1H, $J_{H5'a-H5'b}$ = 12.0 Hz, $J_{H5'a-H4'}$ = $J_{H5'a-OH}$ = 2.5 Hz, $H_{5'a}$), 3.72 (ddd, 1H, $J_{H5'b-H5'a}$ = 12.0 Hz, $J_{H5'b-H4'}$ = 2.5 Hz, $J_{H5'b-OH}$ = 8.5 Hz, $H_{5'b}$), 3.21 (dd, 1H, $J_{OH-H5'a}$ = 2.5 Hz, $J_{OH-H5'b}$ = 8.5 Hz, OH), 0.92 (s, 9H, $-C(C\underline{H_3})_3$), 0.86 (s, 9H, $-C(C\underline{H_3})_3$), 0.09 (s, 3H, $C\underline{H_3}$), 0.09 (s, 3H, $C\underline{H_3}$), 0.05 (s, 3H, $C\underline{H_3}$), -0.02 (s, 3H, $C\underline{H_3}$); ^{13}C NMR *δ* 162.4 (C_4), 150.9 (C_2), 141.3 (C_6), 131.4 (C_8), 118.6 (C_9), 101.9 (C_5), 94.9 ($C_{1'}$), 86.8 ($C_{4'}$), 73.3 ($C_{2'}$), 72.4 ($C_{3'}$), 62.1 ($C_{5'}$), 43.2 (C_7) 25.9, 25.9 ($-C(\underline{C}H_3)_3$), 18.2, 18.1 ($-\underline{C}(CH_3)_3$), -4.3, -4.4, -4.6, -4.7 ($-(\underline{C}H_3)_2$); HRMS ESI$^+$ calcd for $C_{24}H_{45}N_2O_6Si_2^+$ $(M + H)^+$ 513.2811, found 513.2813.

N^3-Allyl-2’,3’-Di-*O*-(triisopropylsilyl)uridine (5b). Compound **5b** was synthesized according to the general procedure for *N*-allylation of protected uridine derivatives from the compound **5a**[3] (500 mg, 0.89 mmol, 1 equiv). This compound was obtained as a white solid (525 mg, 98% yield): R_f 0.34 (cyclohexane/EtOAc 7:3); mp 188–190 °C; $[\alpha]_D$ – 58 (*c* 0.5, $CHCl_3$); IR (film) 3464, 2945, 2865,

---

[2] Maturano, M.; Catana, D.-A.; Lavedan, P.; Tarrat, N.; Saffon, N.; Payrastre, C.; Escudier, J.-M. *Eur. J. Org. Chem.* **2012**, 721−730.

[3] Hwu, J. R. Jain, M. L.; Tsai ,F.-Y.; Tsay, S.-C. Balakumar , A.; Hakimelahi, G. H. *J. Org. Chem.* **2000**, *65*, 17, 5077-5088.

1697, 1658, 1658, 1455, 1391, 1172; 1095, 991, 824; ^{1}H NMR δ 7.51 (d, $J_{H6\text{-}H5}$ = 8.1 Hz, 1H, H_6), 5.86 (ddt, $J_{H8\text{-}H9a}$ = 16.2 Hz, $J_{H8\text{-}H9b}$ = 10.2 Hz, $J_{H8\text{-}H7a}$ = 6.0 Hz, 1H, H_8), 5.79 (d, $J_{H5\text{-}H6}$ = Hz, 1H, H_5), 5.61 (d, $J_{H1'\text{-}H2'}$ = 7.0 Hz, 1H, $H_{1'}$), 5.33 - 5.25 (m, 1H, H_{9a}), 5.23 - 5.16 (m, 1H, H_{9b}), 4.93 (dd, $J_{H1'\text{-}H2'}$ = 7.0 Hz, $J_{H2'\text{-}H3'}$ = 4.2 Hz, 1H, $H_{2'}$), 4.58 - 4.49 (m, 2H, H_7), 4.42 (dd, $J_{H3'\text{-}H2'}$ = 4.2 Hz, $J_{H3'\text{-}H4'}$ = 1.4 Hz, 1H, $H_{3'}$), 4.17 - 4.09 (m, 1H, $H_{4'}$), 3.90 (dt, $J_{H5'a\text{-}H5'b}$ = 12.3 Hz, $J_{H5b'\text{-}H4'}$ = 2.9 Hz, 1H, $H_{5'b}$), 3.75 (ddd, $J_{H5'a\text{-}H5'b}$ = 12.3 Hz, $J_{H5'a\text{-}OH}$ = 7.8 Hz, $J_{H5'a\text{-}H4'}$ = 2.4 Hz, 1H, $H_{5'a}$), 3.50 (dd, $J_{H5'a\text{-}OH}$ = 7.7 Hz, $J_{H5b'\text{-}H4'}$ = 2.9 Hz, 1H, OH), 1.13 - 1.00 (m, 42H, (-C$\underline{H}$(C$\underline{H}_3$)$_2$)); ^{13}C NMR δ 162.29 (C_4), 150.89 (C_2), 141.75 (C_6), 131.21 (C_8), 118.46 (C_9), 102.04 (C_5), 94.41 ($C_{1'}$), 86.44 ($C_{4'}$), 74.15 ($C_{3'}$), 73.61 ($C_{2'}$), 62.65 ($C_{5'}$), 43.07 (C_7), 18.04, 18.1, 18.0, 17.9, 12.9, 12.6 ((-$\underline{C}$($\underline{C}H_3$)$_3$); HRMS ESI$^+$ calcd for $C_{30}H_{57}N_2O_6Si_2^+$ (M + H)$^+$ 597.3750, found 597.3757.

5′(*R*)-C-(Triethylsilylethynyl)-2′,3′-*O*-isopropylideneuridine (11bb). Compound **6b** was synthesized from compound **1b** (236 mg, 0.73 mmol, 1 equiv) according to the general procedure for 5 the oxidation of uridine derivatives. Complete conversion was observed in 1 h and aldehyde **11b** was directly engaged in alkynylation reaction. Data for **11b**: R_f 0.37 (cyclohexane/EtOAc 1:1); ^{1}H NMR δ 9.37 (s, 1H, $H_{5'}$), 7.20 (d, 1H, $J_{H6\text{-}H5}$ = 8.0 Hz, H_6), 5.83 (ddt, 1H, $J_{H8\text{-}H9a}$ = 17.0 Hz, $J_{H8\text{-}H9b}$ = 10.0 Hz, $J_{H8\text{-}H7a}$ = $J_{H8\text{-}H7b}$ = 6.0 Hz, H_8), 5.81 (d, 1H, $J_{H5\text{-}H6}$ = 8.0 Hz, H_5), 5.48 (s, 1H, $H_{1'}$), 5.25 (dd, 1H, $J_{H3'\text{-}H2'}$ = 6.0 Hz, $J_{H3'\text{-}H4'}$ = 1.5 Hz, $H_{3'}$), 5.24-5.20 (m, 1H, H_{9a}), 5.19-5.16 (m, 1H, H_{9b}), 5.10 (d, 1H, $J_{H2'\text{-}H3'}$ = 6.0 Hz, $H_{2'}$), 4.55 (d, 1H, $J_{H4'\text{-}H3'}$ = 1.5 Hz, $H_{4'}$), 4.51 (ddt, 1H, $J_{H7a\text{-}H7b}$ = 14.5 Hz, $J_{H7a\text{-}H8}$ = 6.0 Hz, $J_{H7a\text{-}H9a}$ = $J_{H7a\text{-}H9b}$ = 1.5 Hz, H_{7a}), 4.42 (ddt, 1H, $J_{H7b\text{-}H7a}$ = 14.5 Hz, $J_{H7b\text{-}H8}$ = 6.0 Hz, $J_{H7b\text{-}H9a}$ = $J_{H7b\text{-}H9b}$ = 1.5 Hz, H_{7b}), 1.55 (s, 3H, $H_{7'}$), 1.38 (s, 3H, $H_{7'}$); ^{13}C NMR δ 199.3 ($C_{5'}$), 162.0 (C_4), 151.1 (C_2), 141.9 (C_6), 131.1 (C_8), 118.5 (C_9), 113.7 ($C_{6'}$), 102.7 (C_5), 101.2 ($C_{1'}$), 94.3 ($C_{2'}$), 85.1, ($C_{4'}$), 84.2 ($C_{3'}$), 43.0 (C_7), 26.6, 24.9 ($C_{7'}$). Compound **11bb** was synthesized according to the general procedure for the 5'-alkynylation of uridine-derived aldehydes: a dropwise addition of the crude aldehyde **6b** (235 mg, 0.73 mmol, 1 equiv) solubilized in dry THF (7.2 mL), on a freshly prepared triethylsilylacetylide magnesium bromide solution (2.18 mmol, 3 equiv) in THF (7.2 mL). After work up, the crude mixture **11bb** was isolated as a 65/35 mixture of diasteromers. Flash chromatography of the residue (cyclohexane/EtOAc 1:1) afforded alcohol **11bb** as a colourless gum (176 mg, 52% combined yield). The major diastereoisomer has been isolated with 24% yield as a colourless gum: R_f 0.34 (cyclohexane/EtOAc 1:1); $[\alpha]_D$ - 30 (*c* 1.0, CH_2Cl_2); IR (film) 3710*br*, 2356*w*, 1704*m*, 1662*s*, 1451*m*, 939*m*; ^{1}H NMR δ 7.54 (d, 1H, $J_{H6\text{-}H5}$ = 8.0 Hz, H_6), 5.85 (ddt, 1H, $J_{H8\text{-}H9a}$ = 17.0 Hz, $J_{H8\text{-}H9b}$ = 10.0 Hz, $J_{H8\text{-}H7a}$ = $J_{H8\text{-}H7b}$ = 6.0 Hz, H_8), 5.77 (d, 1H, $J_{H1'\text{-}H2'}$ = 4.0 Hz, $H_{1'}$), 5.74 (d, 1H, $J_{H5\text{-}H6}$ = 8.0 Hz, H_5), 5.27-5.22 (m, 1H, H_{9a}), 5.19-5.15 (m, 1H, H_{9b}), 5.01 (dd, 1H, $J_{H3'\text{-}H2'}$ = 6.5 Hz, $J_{H3'\text{-}H4'}$ = 2.5 Hz, $H_{3'}$), 4.86 (dd, 1H, $J_{H2'\text{-}H3'}$ = 6.5 Hz, $J_{H2'\text{-}H1'}$ = 4.0 Hz, $H_{2'}$), 4.65-4.62 (m, 1H, $H_{5'}$), 4.56-4.46 (m, 2H, H_7), 4.36 (t, 1H, $J_{H4'\text{-}H3'}$ = $J_{H4'\text{-}H5'}$ = 2.5 Hz, $H_{4'}$), 3.65 (brd, 1H, $J_{OH\text{-}H5'}$ = 3.0 Hz, OH), 1.58 (s, 3H, $H_{11'}$), 1.35 (s, 3H, $H_{11'}$), 0.98 (t, 9H, $J_{H9'\text{-}H8'}$ = 7.5 Hz, $H_{9'}$), 0.62 (q,

6H, $J_{H9'-H8'}$ = 7.5 Hz, $H_{8'}$); ^{13}C NMR δ 162.5 (C_4), 150.9 (C_2), 139.9 (C_6), 131.3 (C_8), 118.5 (C_9), 114.3 ($C_{10'}$), 103.2 ($C_{6'}$), 101.9 (C_5), 95.7 ($C_{1'}$), 90.3 ($C_{7'}$), 87.8 ($C_{4'}$), 83.6 ($C_{2'}$), 80.5 ($C_{3'}$), 63.2 ($C_{5'}$), 43.3 (C_7), 27.3, 25.2 ($C_{11'}$), 7.5 ($C_{9'}$), 4.3 ($C_{8'}$); HRMS ESI$^+$ calcd for $C_{23}H_{35}N_2O_6Si^+$ (M + H)$^+$ 463.2259, found 463.2266.

5′(*R*)-C-(Triethylsilylethynyl)-2′,3′-*O*-isopentylideneuridine (12ab). Compound **7a** was synthesized from compound **2a** (180 mg, 0.57 mmol, 1 equiv) according to the general procedure for the oxidation of uridine derivatives. Complete conversion was observed in 45 min and aldehyde **7a** was directly engaged in alkynylation reaction. Data for **7a:** R_f 0.30 (cyclohexane/EtOAc 1:1); ^1H NMR δ 9.44 (s, 1H, $H_{5'}$), 8.59 (br s, 1H, NH), 7.27 (d, 1H, J_{H6-H5} = 8.0 Hz, H_6), 5.76 (d, 1H, J_{H5-H6} = 8.0 Hz, H_5), 5.50 (s, 1H, $H_{1'}$), 5.24 (dd, 1H, $J_{H3'-H2'}$ = 7.0 Hz, $J_{H3'-H4'}$ = 2.0 Hz, $H_{3'}$), 5.11 (d, 1H, $J_{H2'-H3'}$ = 7.0 Hz, $H_{2'}$), 4.58 (d, 1H, $J_{H4'-H3'}$ = 2.0 Hz, $H_{4'}$), 1.76 (q, 2H, $J_{H7'-H8'}$ = 7.5 Hz, $H_{7'}$), 1.63 (q, 2H, $J_{H7'-H8'}$ = 7.5 Hz, $H_{7'}$), 0.96 (t, 3H, $J_{H8'-H7'}$ = 7.5 Hz, $H_{8'}$), 0.90 (t, 3H, $J_{H8'-H7'}$ = 7.5 Hz, $H_{8'}$); ^{13}C NMR δ 199.4 ($C_{5'}$), 162.6 (C_4), 150.3 (C_2), 143.9 (C_6), 118.3 ($C_{6'}$), 102.8 (C_5), 100.4 ($C_{1'}$), 94.3 ($C_{4'}$), 85.2 ($C_{2'}$), 84.1 ($C_{3'}$), 29.3, 28.8 ($C_{7'}$), 8.3, 8.4 ($C_{8'}$). Compound **12ab** was synthesized according to the general procedure for the 5'-alkynylation of uridine-derived aldehydes: a dropwise addition of a solution of the crude aldehyde **7a** (179 mg, 0.57 mmol, 1 equiv) in dry THF (5.6 mL), on a freshly prepared triethylsilylacetylide magnesium bromide solution (1.49 mmol, 2.6 equiv) in THF (5.6 mL). After work up, the crude mixture **12ab** was isolated as a 65/35 mixture of diasteromers. Flash chromatography of the residue (cyclohexane/EtOAc 7:3 to 6:4) afforded alcohol **12ab** as a white solid (139 mg, 54% combined yield). The major diastereoisomer (5’*R*)-**12ab** has been isolated with 15% yield as a white foam. Data for (5’*R*)-**12ab** R_f 0.37 (Cyclohexane/EtOAc 1/1); mp 149-152 °C; $[\alpha]_D$ - 26 (*c* 0.5, CH_2Cl_2); IR (film) 3320*br,* 2356*w*, 1702*m*, 1664*s*, 1450*m*, 940*m*; ^1H NMR δ 9.50 (br s, 1H, NH), 7.52 (d, 1H, J_{H6-H5} = 8.0 Hz, H_6), 5.81 (d, 1H, $J_{H1'-H2'}$ = 4.0 Hz, $H_{1'}$), 5.73 (dd, 1H, J_{H5-H6} = 8.0 Hz, J_{H5-NH} = 1.5 Hz H_5), 5.03 (dd, 1H, $J_{H3'-H2'}$ = 6.5 Hz, $J_{H3'-H4'}$ = 2.5 Hz, $H_{3'}$), 4.89 (dd, 1H, $J_{H2'-H3'}$ = 6.5 Hz, $J_{H2'-H1'}$ = 4.0 Hz, $H_{2'}$), 4.65 (t, 1H, $J_{H5'-H4'}$ = $J_{H5'-OH}$ = 2.5 Hz, $H_{5'}$), 4.35 (t, 1H, $J_{H4'-H5'}$ = $J_{H4'-H3'}$ = 2.5 Hz, $H_{4'}$), 3.36 (brs, 1H, OH), 1.80 (q, 2H, $J_{H11'-H12'}$ = 7.0 Hz, $H_{11'}$), 1.63 (q, 2H, $J_{H11'-H12'}$ = 7.0 Hz, $H_{7'}$), 1.01 (t, 3H, $J_{H12'-H11'}$ = 7.0 Hz, $H_{12'}$), 0.99 (t, 9H, $J_{H8'-H9'}$ = 8.0 Hz, $H_{8'}$), 0.88 (t, 3H, $J_{H12'-H11'}$ = 7.0 Hz, $H_{12'}$), 0.62 (q, 6H, $J_{H9'-H8'}$ = 8.0 Hz, $H_{9'}$); ^{13}C NMR δ 163.2 (C_4), 150.4 (C_2), 141.2 (C_6), 119.0 ($C_{10'}$), 103.2 ($C_{6'}$), 102.8 (C_5), 94.5 ($C_{1'}$), 90.3 ($C_{7'}$), 87.8 ($C_{4'}$), 83.4 ($C_{2'}$), 80.4 ($C_{3'}$), 63.2 ($C_{5'}$), 29.4, 29.3 ($C_{11'}$), 8.4, 7.9 ($C_{12'}$), 7.5 ($C_{9'}$), 4.3 ($C_{8'}$); HRMS ESI$^+$ calcd for $C_{22}H_{35}N_2O_6Si^+$ (M + H)$^+$ 451.2259, found 451.2270.

5′(*R*)-C-(Triethylsilylethynyl)-2′,3′-*O*-cyclohexylideneuridine (13ab). Compound **8a** was synthesized from compound **3a** (250 mg, 0.77 mmol, 1 equiv) according to the general procedure for the oxidation of uridine derivatives. Complete conversion was observed in 1 h and aldehyde **8a** was directly engaged in alkynylation reaction. Data for **8a:** R_f 0.32 (cyclohexane/EtOAc 6/4); ^1H NMR δ

9.45 (s, 1H, $H_{5'}$), 8.53 (br s, 1H, NH), 7.26 (d, 1H, J_{H6-H5} = 8.0 Hz, H_6), 5.76 (d, 1H, J_{H5-H6} = 8.0 Hz, H_5), 5.50 (s, 1H, $H_{1'}$), 5.22 (dd, 1H, $J_{H3'-H2'}$ = 6.5 Hz, $J_{H3'-H4'}$ = 2.0 Hz, $H_{3'}$), 5.11 (d, 1H, $J_{H2'-H3'}$ = 6.5 Hz, $H_{2'}$), 4.56 (d, 1H, $J_{H4'-H3'}$ = 2.0 Hz, $H_{4'}$), 1.77-1.74 (m, 2H, $H_{8'a}$), 1.70-1.64 (m, 2H, $H_{8'b}$), 1.63-1.55 (m, 4H, $H_{7'}$), 1.44-1.41 (m, 2H, $H_{9'}$); ^{13}C NMR δ 199.3 ($C_{5'}$), 162.7 (C_4), 150.4 (C_2), 144.1 (C_6), 114.7 ($C_{6'}$), 102.9 (C_5), 100.5 ($C_{1'}$), 94.4 ($C_{4'}$), 84.6 ($C_{2'}$), 83.4 ($C_{3'}$), 36.4, 34.4 ($C_{7'}$), 25.1, 24.1 ($C_{8'}$), 23.8 ($C_{9'}$). Compound **13ab** was synthesized according to the general procedure for the 5'-alkynylation of uridine-derived aldehydes: a dropwise addition of a solution of the crude aldehyde **8a** (249 mg, 0.77 mmol, 1 equiv) in dry THF (7.5 mL), on a freshly prepared triethylsilylacetylide magnesium bromide solution (1.89 mmol, 2.45 equiv) in THF (7.5 mL). After work up, the crude mixture **13ab** was isolated as a 70/30 mixture of diasteromers. Flash chromatography of the residue (Cyclohexane/EtOAc 7/3, to 1/1) afforded alcohol (5'*R*)-**13ab** as a white solid (225 mg, 65% combined yield). The major diastereoisomer has been isolated with 18% yield as a white foam. Data for (5'*R*)-**13ab** R_f 0.34 (cyclohexane/EtOAc 1:1); mp 150-152 °C; $[\alpha]_D$ - 22 (*c* 0.5, CH_2Cl_2); IR (film) 2936*br*, 1692*s*, 1462*w*, 1275*s*, 1098*m*, 940*m*; ^{1}H NMR δ 8.59 (br s, 1H, NH), 7.50 (d, 1H, J_{H6-H5} = 8.5 Hz, H_6), 5.76 (d, 1H, $J_{H1'-H2'}$ = 3.0 Hz, $H_{1'}$), 5.73 (d, 1H, J_{H5-H6} = 8.5 Hz, H_5), 5.02 (dd, 1H, $J_{H3'-H2'}$ = 6.5 Hz, $J_{H3'-H4'}$ = 3.0 Hz, $H_{3'}$), 4.91 (dd, 1H, $J_{H2'-H3'}$ = 6.5 Hz, $J_{H2'-H1'}$ = 3.0 Hz, $H_{2'}$), 4.66 (t, 1H, $J_{H5'-H4'}$ = $J_{H5'-OH}$ = 3.0 Hz, $H_{5'}$), 4.36 (t, 1H, $J_{H4'-H5'}$ = $J_{H4'-H3'}$ = 3.0 Hz, $H_{4'}$), 3.21 (d, 1H, $J_{OH-H5'}$ = 3.0 Hz, OH), 1.80-1.77 (m, 2H, $H_{12'a}$), 1.70-1.65 (m, 2H, $H_{12'b}$), 1.65-1.52 (m, 4H, $H_{11'}$), 1.46-1.34 (m, 2H, $H_{13'}$) 1.10 (t, 9H, $J_{H9'-H8'}$ = 7.5 Hz, $H_{9'}$), 0.62 (q, 6H, $J_{H9'-H8'}$ = 7.5 Hz, $H_{8'}$); ^{13}C NMR δ 162.8 (C_4), 150.3 (C_2), 142.2 (C_6), 115.4 ($C_{10'}$), 103.1 ($C_{6'}$), 102.9 (C_5), 95.0 ($C_{1'}$), 90.4 ($C_{7'}$), 87.5 ($C_{4'}$), 82.4 ($C_{2'}$), 79.8 ($C_{3'}$), 63.4 ($C_{5'}$), 37.3, 34.7 ($C_{11'}$), 25.1, 24.1 ($C_{12'}$), 23.6 ($C_{13'}$), 7.5 ($C_{9'}$), 4.3 ($C_{8'}$); HRMS ESI$^-$ calcd for $C_{23}H_{33}N_2O_6Si^-$ (M - H)$^-$ 461.2113, found 461.2125.

5'(*S*)-(Triethylsilylethynyl)-2',3'-di-*O*-(*tert*-butyldimethylsilyl)uridine (14ab). Compound **9a** was synthesized from alcohol **4a** (370 mg, 0.78 mmol, 1 equiv) according to the general procedure for the oxidation of uridine derivatives. Complete conversion was observed in 45 min and aldehyde **9a** was directly engaged in alkynylation reaction. Spectral data were in agreement with literature.[2] Compound **14ab** was synthesized according to the general procedure for the 5'-alkynylation of uridine-derived aldehydes: a dropwise addition of a solution of the crude aldehyde **9a** (370, mg, 0.78 mmol, 1 equiv) in dry THF (7.7 mL), on a freshly prepared triethylsilylacetylide magnesium bromide solution (1.92 mmol, 2.5 equiv) in THF (7.7 mL). After work up, the crude mixture **14ab** was isolated as a 85/15 mixture of diasteromers. Flash chromatography of the residue (cyclohexane/EtOAc 8:2 to 7:3) afforded **14ab** as a white solid (308 mg, 64% combined yield). The major diastereoisomer (5'*S*)-**14ab** has been isolated with 48% yield. Data for (5'*S*)-**14ab**: R_f 0.47 (cyclohexane/EtOAc = 1:1); mp 182 °C; $[\alpha]_D$ - 42 (*c* 0.5, CH_2Cl_2); IR (film) 3201*br*, 2870*w*, 1670*s*, 1460*m*, 1362*m*, 1081*s*, 723*s*; ^{1}H NMR δ 9.83 (s, 1H, NH), 7.60 (d, 1H, J_{H6-H5} = 8.0 Hz, H_6), 5.73 (dd,

1H, $J_{H5\text{-}H6}$ = 8.0 Hz, $J_{H5\text{-}NH}$ = 1.5 Hz, H_5), 5.61 (d, 1H, $J_{H1'\text{-}H2'}$ = 6.0 Hz, $H_{1'}$), 4.62 (t, 1H, $J_{H3'\text{-}H2'}$ = 5.0 Hz, $J_{H3'\text{-}H4'}$ = 5.0 Hz, $H_{3'}$), 4.54 (dd, 1H, $J_{H2'\text{-}H3'}$ = 5.0 Hz, $J_{H2'\text{-}H1'}$ = 6.0 Hz, $H_{2'}$), 4.17 (dd, 1H, $J_{H5'\text{-}H4'}$ = 5.0 Hz, $J_{H5'\text{-}OH'}$ = 3.5 Hz, $H_{5'}$), 4.02 (t, 1H, $J_{H4'\text{-}H5'}$ = 5.0 Hz, $J_{H4'\text{-}H3'}$ = 5.0 Hz, $H_{4'}$), 3.50-3.45 (m, 1H, OH), 0.99 (t, 9H, $J_{H9'\text{-}H8'}$ = 8.0 Hz, $H_{9'}$), 0.89 (s, 9H, -C(C$\underline{H_3}$)$_3$), 0.86 (s, 9H, -C(C$\underline{H_3}$)$_3$), 0.62 (q, 6H, $J_{H8'\text{-}H9'}$ = 8.0 Hz, $H_{8'}$), 0.07 (s, 6H, C$\underline{H_3}$), 0.05 (s, 3H, C$\underline{H_3}$), 0.03 (s, 3H, C$\underline{H_3}$); ^{13}C NMR δ 162.9 (C_4), 150.4 (C_2), 143.0 (C_6), 104.8 ($C_{6'}$), 102.4 (C_5), 93.2 ($C_{1'}$), 89.3 ($C_{7'}$), 87.6 ($C_{4'}$), 73.4 ($C_{2'}$), 72.5 ($C_{3'}$), 62.5 ($C_{5'}$), 25.9, 25.9 (-C($\underline{C}H_3$)$_3$), 18.2, 18.0 (-$\underline{C}$(CH$_3$)$_3$), 7.5 ($C_{8'}$), 4.4 ($C_{9'}$), -4.4, -4.4, -4.3, -4.7 (-($\underline{C}H_3$)$_2$); HRMS HRMS ESI$^+$ calcd for $C_{29}H_{55}N_2O_6Si_3^+$ (M + H)$^+$ 611.3368, found 611.3371. **Caracteristic signals for (5'*R*)-14ab:** ^{1}HNMR δ 9.68 (s, 1H, NH), 7.63 (d, 1H, $J_{H6\text{-}H5}$ = 8.0 Hz, H_6), 4.30-4.26 (m, 1H, $H_{4'}$), 4.10-4.09 (m, 1H, $H_{2'}$).

***N*^{3}-Allyl-2',3'-di-*O*-(*tert*-butyldimethylsilyl)-5'-(triethylsilylethynyl)-uridine (14bb).** Compound **14bb** was synthesized according to the general procedure for 5'-uridine aldehydes alkynylation by a dropwise addition of a solution of the crude aldehyde **9b** (synthesized according to the general procedure for 5'-uridine aldehydes preparation from corresponding alcohol **4b**, (400 mg, 0.78 mmol, 1 equiv)) in dry THF (7.8 mL), on a freshly prepared triethylsilylacetylide magnesium bromide solution (1.95 mmol, 2.5 equiv) in THF (7.8 mL). After work up, the crude mixture **14bb** was isolated as a 91:9 mixture of diasteromers. Flash chromatography of the residue (cyclohexane/EtOAc 9:1) afforded **14bb** as a white solid 300 mg, 59% combined yield), the major diastereoisomer **14bb** has been isolated with 39% yield as a viscous oil: R$_f$ 0.38 (cyclohexane/EtOAc 8:2); $[\alpha]_D$ – 13 (*c* 0.5, CHCl$_3$); IR (film) 2953, 2929, 2858, 2357, 2342, 1716, 1663, 1457, 1388, 1253, 1102, 932; ^{1}H NMR δ 7.47 (d, $J_{H6\text{-}H5}$ = 8.1 Hz, 1H, H_6), 5.84 (ddt, $J_{H8\text{-}H9a}$ = 16.2 Hz, $J_{H8\text{-}H9b}$ = 10.2 Hz, $J_{H8\text{-}H7a}$ = $J_{H8\text{-}H7b}$ = 6.0 Hz, 1H, H_8), 5.76 (d, $J_{H5\text{-}H6}$ = 8.1 Hz, 1H, H_5), 5.61 (d, $J_{H1'\text{-}H2'}$ = 6.9 Hz, 1H, $H_{1'}$), 5.31 - 5.23 (m, 1H, H_{9a}), 5.20 - 5.13 (m, 1H, H_{9b}), 4.62 - 4.54 (m, 2H, $H_{2'}$ + $H_{5'}$), 4.54 - 4.49 (m, 2H, H_7), 4.15 (dd, 1H, $J_{H3'\text{-}H2'}$ = 4.6 Hz, $J_{H3'\text{-}H4'}$ = 1.6 Hz, $H_{3'}$), 4.04 (dd, $J_{H4'\text{-}H5'}$ =4.2 Hz, $J_{H4'\text{-}H3'}$ = 1.6 Hz, 1H, $H_{4'}$), 3.47 (br s, 1H, OH), 0.97 (t, $J_{H9'\text{-}H8'}$ = 7.9 Hz, 1H, $H_{9'}$), 0.91 (s, 9H, -C(C$\underline{H_3}$)$_3$), 0.83 (s, 9H, -C(C$\underline{H_3}$)$_3$), 0.60 (q, $J_{H8'\text{-}H9'}$ = 7.9 Hz, 6H, $H_{8'}$), 0.10 (s, 3H, C$\underline{H_3}$), 0.09 (s, 3H, C$\underline{H_3}$), 0.01 (s, 3H, C$\underline{H_3}$), -0.08 (s, 3H, C$\underline{H_3}$); δ 162.3 (C_4), 150.8 (C_2), 141.0 (C_6), 131.3 (C_8), 118.5 (C_9), 104.8 ($C_{6'}$), 102.0 (C_5), 93.5 ($C_{1'}$), 89.0 ($C_{7'}$), 88.00 ($C_{4'}$), 73.1 ($C_{2'}$), 72.7 ($C_{3'}$), 62.8 ($C_{5'}$), 43.17 (C_7), 25.9, 25.8 ((-C($\underline{C}H_3$)$_3$), 18.1, 18.3 (-$\underline{C}$(CH$_3$)$_3$), , 7.50 ($C_{9'}$), 4.33 ($C_{8'}$), -4.3, -4.4, -4.4, -4.8 ((-($\underline{C}H_3$)$_2$); HRMS ESI$^+$ calcd for $C_{32}H_{59}N_2O_6Si_3^+$ (M + H)$^+$ 651.3675, found 651.3680.

5'(S)-(Trimethylsilylethynyl)-2',3'-di-*O*-(triisopropylsilyl)-uridine (15aa). Compound **15aa** was synthesized according to the general procedure for the 5'-alkynylation of uridine-derived aldehydes: a dropwise addition of a solution of the crude aldehyde **10a** (synthesized from alcohol **5a**[3] (450 mg, 0.80 mmol, 1 equiv) according to the general procedure for the oxidation of uridine derivatives) in dry THF (8.0 mL), on a freshly prepared trimethylsilylacetylide magnesium bromide solution (4.04

mmol, 5 equiv) in THF (8.0 mL). After work up, the crude mixture **15aa** was isolated as a 90:10 mixture of diasteromers. Flash chromatography of the residue (cyclohexane/EtOAc 7:3) afforded **15aa** as a white solid (348 mg, 66% combined yield). The major diastereoisomer (5'*S*)-**15aa** has been isolated with 36% yield as a white solid. R_f 0.39 (cyclohexane/EtOAc 7:3); mp 195 - 199 °C; $[\alpha]_D$ - 40 (*c* 0.2, $CHCl_3$); IR (film) 2944, 2867, 1712, 1681, 1464, 1386, 1262, 1250, 883; ^{1}H NMR δ 9.28 (br s, 1H, NH), 7.30 (d, $J_{H6\text{-}H5}$ = 8.0 Hz, 1H, H_6), 5.56 (d, $J_{H5\text{-}H6}$ = 8.0 Hz, 1H, H_5), 5.42 (d, $J_{H1'\text{-}H2'}$ = 7.0 Hz, 1H, $H_{1'}$), 4.73 (dd, $J_{H2'\text{-}H1'}$ = 7.0 Hz, $J_{H2'\text{-}H3'}$ = 4.0 Hz, 1H, $H_{2'}$), 4.51 (t, $J_{H5'\text{-}H4'}$ = 5.4 Hz, $J_{H5'\text{-}OH'}$ = 5.4 Hz, 1H, $H_{5'}$), 4.26 (dd, $J_{H3'\text{-}H2'}$ = 4.0 Hz, $J_{H3'\text{-}H4'}$ = 1.0 Hz, 1H, $H_{3'}$), 3.92 (dd, $J_{H4'\text{-}H5'}$ = 5.4 Hz, $J_{H4'\text{-}H3'}$ = 1.0 Hz, 1H, $H_{4'}$), 3.46 (d, $J_{H5'\text{-}OH}$ = 5.4 Hz, 1H, OH), 0.84 - 0.96 (m, 42H, -C$\underline{H}$(C$\underline{H}_3$)$_2$), -0.00 (s, 9H, Si(C$\underline{H}_3$)$_3$); ^{13}C NMR δ 163.5 (C_4), 150.8 (C_2), 144.1 (C_6), 103.5 ($C_{6'}$), 102.7 (C_5), 94.1 ($C_{1'}$), 91.9 ($C_{7'}$), 87.6 ($C_{4'}$), 74.2 ($C_{3'}$), 73.4 ($C_{2'}$), 62.8 ($C_{5'}$), 18.46 (-CH($\underline{C}H_3$)$_2$), 18.42 (-CH($\underline{C}H_3$)$_2$), 18.22 (-CH($\underline{C}H_3$)$_2$), 18.08 (-CH($\underline{C}H_3$)$_2$), 13.15 (-$\underline{C}$H(CH_3)$_2$), 12.84 (-$\underline{C}$H(CH_3)$_2$), 0.00 (-Si($\underline{C}H_3$)$_3$); HRMS ESI^+ calcd for $C_{32}H_{61}N_2O_6Si_3^+$ $(M + H)^+$ 653.3837, found 653.3832.

5'(*S*)-(Triethylsilylethynyl)-2',3'-di-*O*-(triisopropylsilyl)-uridine (15ab). Compound **15ab** was synthesized according to the general procedure for the 5'-alkynylation of uridine-derived aldehydes: a dropwise addition of a solution of the crude aldehyde **10a** (synthesized from corresponding alcohol **5a**[3] (500 mg, 0.90 mmol, 1 equiv) according to the general the oxidation of uridine derivatives) in dry THF (9.0 mL), on a freshly prepared triethylsilylacetylide magnesium bromide solution (4.50 mmol, 5 equiv) in THF (9.0 mL). After work up, the crude mixture **15ab** was isolated as a 95:5 mixture of diastereoisomers. Flash chromatography of the residue (cyclohexane/EtOAc 7:3) afforded **15ab** as a white solid 406 mg, 65% combined yield). The major diastereoisomer (5'*S*)-**15ab** has been isolated with 32% yield as a white solid. R_f 0.40 (cyclohexane/EtOAc 7:3); mp 216 - 220 °C; $[\alpha]_D$ -19 (*c* 0.2, $CHCl_3$); IR (film) 3675, 2971, 2900, 1704, 1689, 1463, 1393, 1074, 883; ^{1}H NMR δ 9.34 (br s, 1H, NH), 7.53 (d, $J_{H6\text{-}H5}$ = 8.0 Hz, 1H, H_6), 5.75 (dd, $J_{H5\text{-}H6}$ = 8.0 Hz, $J_{H5\text{-}NH}$ = 2.2 Hz, 1H, H_5), 5.62 (d, $J_{H1'\text{-}H2'}$ = 6.8 Hz, 1H, $H_{1'}$), 4.90 (dd, $J_{H2'\text{-}H1'}$ = 6.8 Hz, $J_{H2'\text{-}H3'}$ = 4.0 Hz, 1H, $H_{2'}$), 4.66 (t, $J_{H5'\text{-}H4'}$ = 5.5 Hz, $J_{H5'\text{-}OH'}$ = 5.5 Hz, 1H, $H_{5'}$), 4.42 (dd, $J_{H3'\text{-}H2'}$ = 4.0 Hz, $J_{H3'\text{-}H4'}$ = 1.5 Hz, 1H, $H_{3'}$), 4.11 (dd, $J_{H4'\text{-}H5'}$ = 4.8 Hz, $J_{H4'\text{-}H3'}$ = 1.5 Hz, 1H, $H_{4'}$), 3.54 (d, $J_{H5'\text{-}OH}$ = 5.5 Hz, 1H, OH), 1.15 - 1.01 (m, 42H, -C$\underline{H}$(C$\underline{H}_3$)$_2$), 0.98 (t, $J_{H9'\text{-}H8'}$ = 7.9 Hz, 9H, $H_{9'}$), 0.61 (q, $J_{H8'\text{-}H9'}$ = 7.9 Hz, 6H, $H_{8'}$); ^{13}C NMR δ 163.3 (C_4), 150.4 (C_2), 143.7 (C_6), 104.4 ($C_{6'}$), 102.3 (C_5), 93.6 ($C_{1'}$), 89.0 ($C_{7'}$), 87.4 ($C_{4'}$), 73.9 ($C_{3'}$), 73.4 ($C_{2'}$), 62.6 ($C_{5'}$), 18.2 (-CH($\underline{C}H_3$)$_2$), 18.1 (-CH($\underline{C}H_3$)$_2$), 17.9 (-CH($\underline{C}H_3$)$_2$), 17.8 (-CH($\underline{C}H_3$)$_2$), 12.8 (-$\underline{C}$H(CH_3)$_2$), 12.6 (-$\underline{C}$H(CH_3)$_2$), 7.3 ($C_{9'}$), 4.1 ($C_{8'}$); HRMS ESI^+ calcd for $C_{35}H_{67}N_2O_6Si_3^+$ $(M + H)^+$ 695.4307, found 695.4311.

5'(*S*)-(Trisopropylsilylethynyl)-2',3'-Di-*O*-(triisopropylsilyl)uridine (15ac). Compound **15ac** was synthesized according to the general procedure for the 5'-alkynylation of uridine-derived aldehydes: a dropwise addition of a solution of the crude aldehyde **10a (**synthesized according to the general

procedure for 5'-uridine aldehydes preparation from corresponding alcohol **5a**,[3] (500 mg, 0.90 mmol, 1 equiv)) in dry THF (9 mL), on a previously prepared triethylsilylacetylide magnesium bromide solution (4.50 mmol, 5 equiv) in THF (9 mL). After work up, the crude mixture **15ac** was isolated as a 99/1 mixture of diasteromers. Flash chromatography of the residue (cyclohexane/EtOAc 8:2) afforded **15ac** as a white solid 404 mg, 61% combined yield), the major diastereoisomer (5'*S*)-**15ac** has been isolated with 61% yield as a white solid: R_f 0.39 (cyclohexane/EtOAc 7:3); mp > 260 °C; $[\alpha]_D$ – 2 (*c* 0.5, $CHCl_3$); IR (film) 3852, 3661, 2971, 2900, 1684, 1405, 1394, 1382, 1241, 1229, 882, 647; ^{1}H NMR δ 8.26 (br s, 1H, NH), 7.54 (d, $J_{H6\text{-}H5}$ = 8.0 Hz, 1H, H_6), 5.72 (d, $J_{H5\text{-}H6}$ = 8.0 Hz, $J_{H5\text{-}NH}$ = 2.4 Hz, 1H, H_5), 5.59 (d, $J_{H1'\text{-}H2'}$ = 6.8 Hz, 1H, $H_{1'}$), 4.89 (dd, $J_{H2'\text{-}H1'}$ = 6.8 Hz, $J_{H2'\text{-}H3'}$ = 4.0 Hz, 1H, $H_{2'}$), 4.57 (dd, $J_{H5'\text{-}OH'}$ = 5.8 Hz, $J_{H5'\text{-}H4'}$ = 3.6 Hz, 1H, $H_{5'}$), 4.37 (dd, $J_{H3'\text{-}H2'}$ = 4.0 Hz, $J_{H3'\text{-}H4'}$ = 1.5 Hz, 1H, $H_{3'}$), 4.11 (dd, $J_{H4'\text{-}H5'}$ = 3.6 Hz, $J_{H4'\text{-}H3'}$ = 1.5 Hz, 1H, $H_{4'}$), 3.35 (d, $J_{H5'\text{-}OH}$ = 5.8 Hz, 1H, OH), 1.11 - 1.01 (m, 63H, (-C$\underline{H}$(C$\underline{H_3}$)$_2$)); ^{13}C NMR δ 162.7 (C_4), 150.3 (C_2), 143.8 (C_6), 105.2 ($C_{6'}$), 102.4 (C_5), 93.7 ($C_{1'}$), 88.0 ($C_{7'}$), 87.7 ($C_{4'}$), 74.2 ($C_{3'}$), 73.6 ($C_{2'}$), 63.0 ($C_{5'}$), 18.6 (-CH($\underline{C}H_3$)$_2$, 18.3 (-CH($\underline{C}H_3$)$_2$), 18.2 (-CH($\underline{C}H_3$)$_2$) , 18.1 (-CH($\underline{C}H_3$)$_2$), 18.0 (-$\underline{C}$H(CH_3)$_2$), 13.0 (-$\underline{C}$H(CH_3)$_2$), 12.8 (-$\underline{C}$H(CH_3)$_2$), 11.2 (-CH($\underline{C}H_3$)$_2$); HRMS ESI$^+$ calcd for $C_{38}H_{73}N_2O_6Si_3^+$ (M + H)$^+$ 737.4771, found 737.4775.

***N*^{3}-Allyl-2',3'-di-*O*-(triisopropylsilyl)-5'-(trisopropylsilylethynyl)-uridine (15bc)**. Compound **15bc** was synthesized according to the general procedure for the 5'-alkynylation of uridine-derived aldehydes: a dropwise addition of a solution of the crude aldehyde **10b** (synthesized according to the general procedure for 5'-uridine aldehydes preparation from corresponding alcohol **5b**, (500 mg, 0.83 mmol, 1 equiv)) in dry THF (8.3 mL), on a freshly prepared triethylsilylacetylide magnesium bromide solution (4.18 mmol, 5 equiv) in THF (9.0 mL). After work up, the crude mixture **15bc** was isolated as a 90:10 mixture of diasteromers. Flash chromatography of the residue (cyclohexane/EtOAc 9:1) afforded **15bc** as a white solid 280 mg, 43% combined yield). The major diastereoisomer **15bc** has been isolated with 27% yield as a yellow solid: R_f 0.54 (cyclohexane/EtOAc 8:2); mp 217-219 °C; $[\alpha]_D$ – 5 (*c* 0.5, $CHCl_3$); IR (film) 3403, 2943, 2865, 1712, 1664, 1459, 1389, 1338, 1140, 938; ^{1}H NMR δ 7.42 (d, $J_{H6\text{-}H5}$ = 8.1 Hz, 1H, H_6), 5.91 - 5.80 (m, 1H, 1H, H_8), 5.77 (d, $J_{H5\text{-}H6}$ = 8.1 Hz, 1H, H_5), 5.56 (d, $J_{H1'\text{-}H2'}$ = 7.3 Hz, 1H, $H_{1'}$), 5.31 - 5.23 (m, 1H, H_{9a}), 5.19 - 5.13 (m, 1H, H_{9b}), 4.97 (dd, $J_{H1'\text{-}H2'}$ = 7.3 Hz, $J_{H2'\text{-}H3'}$ = 4.0 Hz, 1H, $H_{2'}$), 4.58 - 4.48 (m, 3H, H_7 + $H_{5'}$), 4.37 (dd, $J_{H3'\text{-}H2'}$ = 4.0 Hz, $J_{H3'\text{-}H4'}$ = 1.5 Hz, 1H, $H_{3'}$), 4.09 (dd, $J_{H4'\text{-}H5'}$ = 3.4 Hz, $J_{H4'\text{-}H3'}$ = 1.5 Hz, 1H, $H_{4'}$), 3.75 (d, $J_{H5'\text{-}OH}$ = 6.6 Hz, 1H, OH), 0.63 - 1.13 (m, 63H, (-C$\underline{H}$(C$\underline{H_3}$)$_2$)); ^{13}C NMR δ 162.37 (C_4), 150.97 (C_2), 142.23 (C_6), 131.33 (C_8), 118.56 (C_9), 105.43 ($C_{6'}$), 102.19 (C_5), 94.87 ($C_{1'}$), 88.05 ($C_{4'}$), 87.67 ($C_{7'}$), 74.56 ($C_{3'}$), 73.11 ($C_{2'}$), 63.24 ($C_{5'}$), 43.15 (C_7), 18.63, 18.33, 18.27, 18.11, 18.0, 13.0, 12.7, 11.2 ((-$\underline{C}$($\underline{C}H_3$)$_3$); HRMS ESI$^+$ calcd for $C_{41}H_{77}N_2O_6Si_3^+$ (M + H)$^+$ 777.5089, found 777.5104.

5’(*R*)-(Triethylsilylethynyl)uridine (16).

At 0 °C, to a suspension of alcohol (5’*R*)-**11ab1** (113 mg, 0.27 mmol, 1 equiv.) in water (1 mL), TFA was added dropwise (4 mL). The mixture was stirred at 0 °C for 10 min and then at r.t. for 1 h 30. The mixture was then concentrated to dryness and the residue was purified by flash chromatography (EtOAc) to furnish the triol (5’*R*)-**16** as a white foam (103 mg, 99% yield): R_f 0.12 (EtOAc); mp 167-169 °C; $[\alpha]_D$ - 34 (*c* 0.8, MeOH); IR (film) 3199*br*, 2830*w*, 1665*s*, 1461*m*, 1352*m*, 1080*s*, 756*s*; ^{1}H NMR (CD_3OD) δ 7.98 (d, 1H, $J_{H6\text{-}H5}$ = 8.0 Hz, H_6), 6.02 (d, 1H, $J_{H1'\text{-}H2'}$ = 7.0 Hz, $H_{1'}$), 5.68 (d, 1H, $J_{H5\text{-}H6}$ = 8.0 Hz, H_5), 4.57-4.55 (m, 1H, $H_{5'}$), 4.29 (d, 1H, $J_{H3'\text{-}H2'}$ = 6.0 Hz, $H_{3'}$), 4.23 (dd, 1H, $J_{H2'\text{-}H3'}$ = 6.0 Hz, $J_{H2'\text{-}H1'}$ = 7.0 Hz, $H_{2'}$), 4.11-4.09 (m, 1H, $H_{4'}$), 1.01 (t, 9H, $J_{H9'\text{-}H8'}$ = 8.0 Hz, $H_{9'}$), 0.60 (q, 6H, $J_{H8'\text{-}H9'}$ = 8.0 Hz, $H_{8'}$); ^{13}C NMR δ 165.9 (C_4), 152.6 (C_2), 142.5 (C_6), 103.1 ($C_{6'}$), 102.8 (C_5), 89.3 ($C_{1'}$), 89.5 ($C_{7'}$), 88.9 ($C_{4'}$), 75.6 ($C_{2'}$), 71.9 ($C_{3'}$), 63.9 ($C_{5'}$), 7.8 ($C_{9'}$), 5.1 ($C_{8'}$); HRMS ESI$^+$ calcd for $C_{17}H_{27}N_2O_6Si^+$ $(M + H)^+$ 383.1633, found 383.1632.

5’(*S*)-(Triethylsilylethynyl)uridine (16).

At 0 °C, to a suspension of alcohol (5’*S*)-**11ab**[1] (68 mg, 0.11 mmol, 1 equiv.) in water (650 µL), TFA was added dropwise (1.65 mL). The mixture was stirred at 0 °C for 10 min and then at r.t. for 18 h. The mixture was then concentrated to dryness and the residue was purified by flash chromatography (EtOAc) to furnish the triol (5’*S*)-**16** as a white foam (38 mg, 91% yield): R_f 0.12 (EtOAc); mp 132-135 °C; $[\alpha]_D$ + 12 (*c* 0.9, MeOH); IR (film) 3201*br*, 2842*w*, 1667*s*, 1452*m*, 1356*m*, 1080*s*, 820*s*; ^{1}H NMR (CD_3OD) δ 7.98 (d, 1H, $J_{H6\text{-}H5}$ = 8.0 Hz, H_6), 5.98 (d, 1H, $J_{H1'\text{-}H2'}$ = 6.0 Hz, $H_{1'}$), 5.68 (d, 1H, $J_{H5\text{-}H6}$ = 8.0 Hz, H_5), 4.55 (d, 1H, $J_{H5'\text{-}H4'}$ = 3.0 Hz $H_{5'}$), 4.25 (t, 1H, $J_{H2'\text{-}H3'}$ = 6.0 Hz, $J_{H2'\text{-}H1'}$ = 6.0 Hz, $H_{2'}$), 4.20 (dd, 1H, $J_{H3'\text{-}H2'}$ = 6.0 Hz, $J_{H3'\text{-}H4'}$ = 3.0 Hz, $H_{3'}$), 4.05 (t, 1H, $J_{H4'\text{-}H3'}$ = 3.0 Hz, $J_{H4'\text{-}H5'}$ = 3.0 Hz, $H_{4'}$), 1.01 (t, 9H, $J_{H9'\text{-}H8'}$ = 8.0 Hz, $H_{9'}$), 0.63 (q, 6H, $J_{H8'\text{-}H9'}$ = 8.0 Hz, $H_{8'}$); ^{13}C NMR δ 166.2 (C_4), 152.7 (C_2), 142.6 (C_6), 107.5 ($C_{6'}$), 103.0 (C_5), 89.8 ($C_{1'}$), 88.6 ($C_{7'}$), 88.4 ($C_{4'}$), 75.7 ($C_{2'}$), 71.9 ($C_{3'}$), 63.4 ($C_{5'}$), 7.8 ($C_{9'}$), 5.3 ($C_{8'}$); HRMS ESI$^+$ Calcd for $C_{17}H_{27}N_2O_6Si^+$ $(M + H)^+$ 383.1633, found 383.1634.

5’(*R*)-(ethynyl)-uridine (17).

Compound **17** has been synthesized from alkyne (5’*R*)-**11ab**[1] (40 mg, 0.13 mmol, 1 equiv) according to the procedure used for the preparation of (5’*S*)-**17**. Crystallization of the residue (DCM/MeOH 9/1) furnished (5’*R*)-**17** as white crystals (32 mg, 92% yield); R_f 0.23 (DCM/MeOH 80/20); mp 224 °C; $[\alpha]_D$ – 27 (*c* 0.9, H_2O); IR (film) 3270*s*, 2852m, 1682*s*, 1456*m*, 1383*m*; ^{1}H NMR (CD_3OD) δ 8.00 (d, 1H, $J_{H6\text{-}H5}$ = 8.5 Hz, H_6), 6.04 (d, 1H, $J_{H1'\text{-}H2'}$ = 7.0 Hz, $H_{1'}$), 5.71 (d, 1H, $J_{H5\text{-}H6}$ = 8.5 Hz, H_5), 4.53 (t, 1H, $J_{H5'\text{-}H4'}$ = $J_{H5'\text{-}H7'}$ = 2.5 Hz, $H_{5'}$), 4.30 (dd, 1H, $J_{H3'\text{-}H2'}$ = 5.5 Hz, $J_{H3'\text{-}H4'}$ = 2.5 Hz, $H_{3'}$) 4.22 (dd, 1H, $J_{H2'\text{-}H1'}$ = 7.0 Hz, $J_{H2'\text{-}H3'}$ = 5.5 Hz, $H_{2'}$), 4.07 (t, 1H, $J_{H4'\text{-}H3'}$ = $J_{H4'\text{-}H5'}$ = 2.5 Hz, $H_{4'}$), 2.98 (d, 1H, $J_{H7'\text{-}H5'}$ = 2.5 Hz, $H_{7'}$); ^{13}C NMR (CD_3OD) δ 166.2 (C_4), 152.8 (C_2), 142.7 (C_6), 103.2 (C_5), 89.5

($C_{1'}$), 89.2 ($C_{4'}$), 82.8 ($C_{6'}$), 76.2 ($C_{2'}$), 75.6 ($C_{7'}$), 71.9 ($C_{2'}$), 63.3 ($C_{5'}$); HRMS ESI$^-$ calcd for $C_{11}H_{11}N_2O_6^+$ (M + H)$^-$ 267.0626, found 267.0623.

5'(*S*)-(ethynyl)-uridine (17).

On a solution of alcohol (5'*S*)-**11ab**[1] (56 mg, 0.132 mmol, 1 equiv.) in MeOH (1,5 mL) was added dropwise at 0 °C a solution 1.5 M of sodium hydroxide. After 5 h, a saturated aqueous solution of NH_4Cl was added dropwise and MeOH was removed in vacuo. The aqueous phase was then extracted with EtOAc and the combined organic layers were dried over Na_2SO_4, filtered and concentrated in vacuo. The crude terminal alkyne (40 mg, 0.130 mmol) was crystallised and immediately deprotected. To a suspension of terminal alkyne in pure H_2O (0.7 mL), TFA (2.8 mL) was added dropwise at 0 °C. The pale yellow resulting solution was stirred at r.t. (TLC monitoring) and then concentrated to dryness. Flash chromatography of the residue (DCM/MeOH 95/5 to 90/10) afforded (5'*S*)-**17** as a white solid (18.6 mg, 72% yield); R_f 0.23 (DCM/MeOH 80/20); mp 220-222 °C; $[\alpha]_D$ + 40 (*c* 0.1, MeOH); IR (film) 3270*br*, 2852m, 1682*s*, 1456*m*, 1383*m*; ^{1}H NMR (CD_3OD) δ 8.00 (d, 1H, $J_{H6\text{-}H5}$ = 8.0 Hz, H_6), 5.98 (d, 1H, $J_{H1'\text{-}H2'}$ = 6.0 Hz, $H_{1'}$), 5.72 (d, 1H, $J_{H5\text{-}H6}$ = 8.0 Hz, H_5), 4.54 (dd, 1H, $J_{H5'\text{-}H4'}$ = 3.5 Hz, $J_{H5'\text{-}H7'}$ = 2.5 Hz, $H_{5'}$), 4.23 (t, 1H, $J_{H2'\text{-}H1'}$ = $J_{H2'\text{-}H3'}$ = 6.0 Hz, $H_{2'}$), 4.20 (dd, 1H, $J_{H3'\text{-}H2'}$ = 6.0 Hz, $J_{H3'\text{-}H4'}$ = 3.5 Hz, $H_{3'}$), 4.03 (t, 1H, $J_{H4'\text{-}H3'}$ = $J_{H4'\text{-}H5'}$ = 3.5 Hz, $H_{4'}$), 2.91 (d, 1H, $J_{H7'\text{-}H5'}$ = 2.5 Hz, $H_{7'}$); ^{13}C NMR (CD_3OD) δ 166.2 (C_4), 152.7 (C_2), 142.7 (C_6), 103.1 (C_5), 90.0 ($C_{1'}$), 88.4 ($C_{4'}$), 83.7 ($C_{6'}$), 75.4 ($C_{3'}$), 75.2 ($C_{7'}$), 71.9 ($C_{2'}$), 62.7 ($C_{5'}$); HRMS ESI$^-$ calcd for $C_{11}H_{11}N_2O_6^+$ (M + H)$^-$ 267.0626, found 267.0623.
