# Supplementary material for: Effect of uridine protecting groups on the diastereoselectivity of uridine-derived aldehyde 5’-alkynylation
Source: Beilstein J Org Chem. 2017 Aug 4;13:1533–41. doi: 10.3762/bjoc.13.153 (PMC5550804; doi:10.3762/bjoc.13.153)

Supporting Information

for

Effect of uridine protecting groups on the diastereoselectivity of uridine-derived aldehyde 5’-alkynylation

Raja Ben Othman[‡], Mickaël J. Fer[‡], Laurent Le Corre, Sandrine Calvet-Vitale* and Christine Gravier-Pelletier*

Address: Laboratoire de Chimie et Biochimie Pharmacologiques et Toxicologiques, UMR 8601 CNRS, Université Paris Descartes, Sorbonne Paris Cité (USPC), Centre Interdisciplinaire Chimie Biologie-Paris (CICB-Paris), 45 rue des Saints Pères, 75270 Paris 06, France.

Email: Sandrine Calvet-Vitale - sandrine.calvet-vitale@parisdescartes.fr; Christine Gravier-Pelletier - christine.gravier-pelletier@parisdescartes.fr

*Corresponding author

[‡]Equal contributors

Spectral data for new compounds

^{1}H NMR, compound **1b**

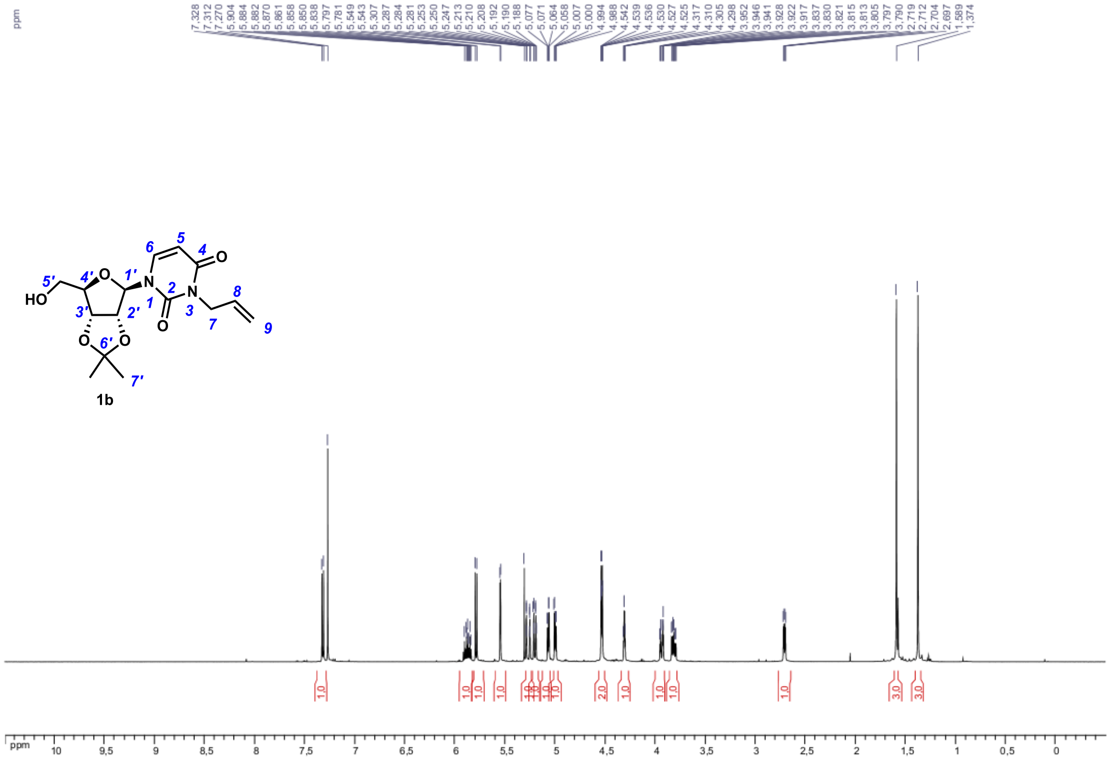

^{13}C NMR

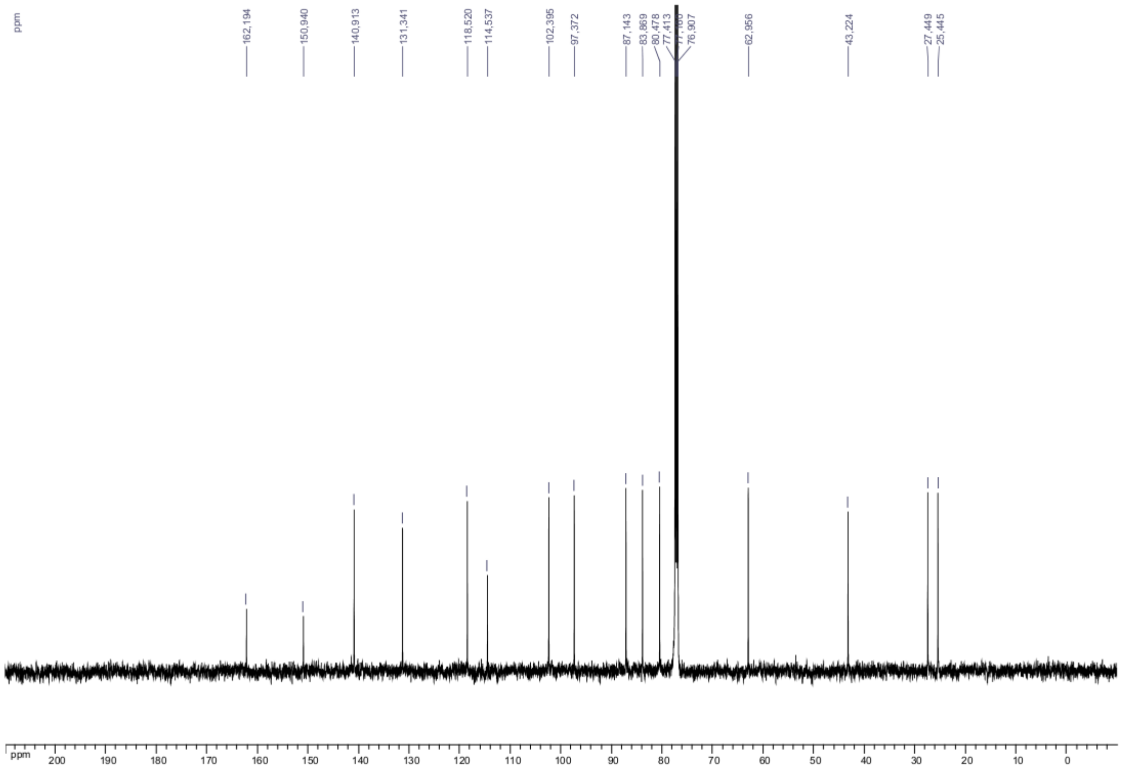

^{1}H NMR, compound **2a**

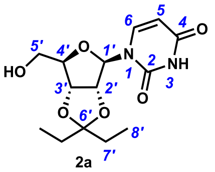

2a

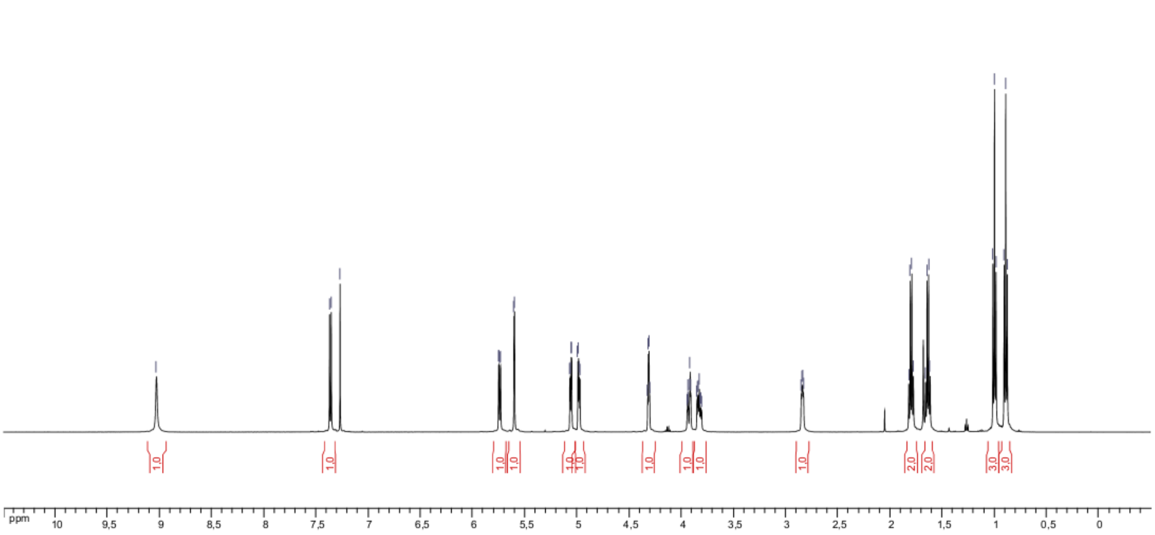

^{13}C NMR

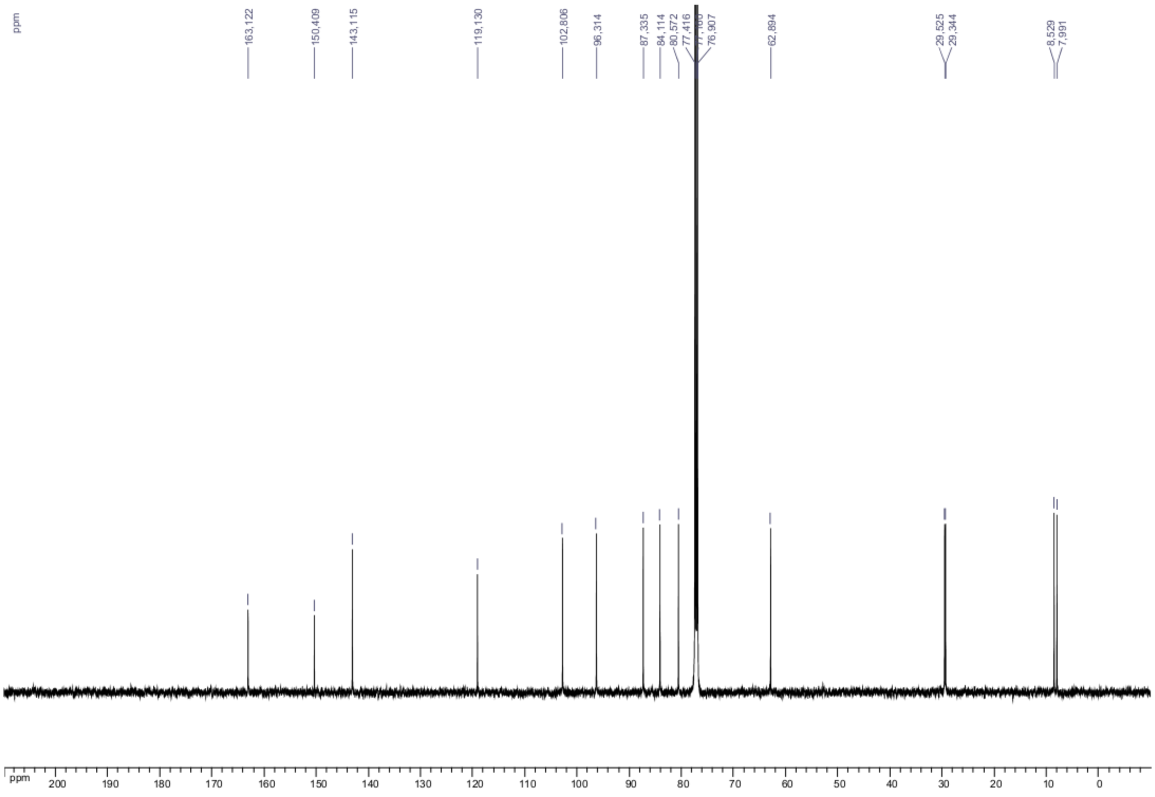

^{1}H NMR, compound **3a**

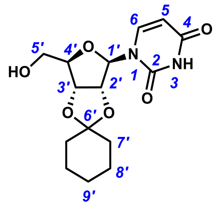

3a

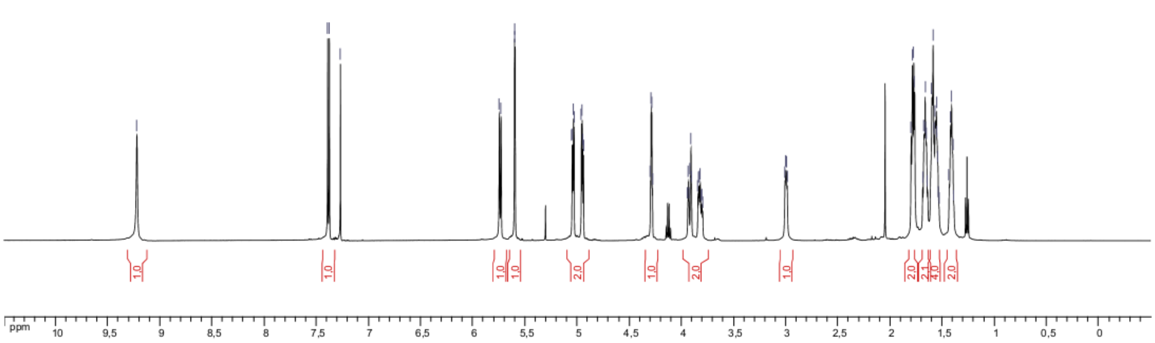

^{13}C NMR

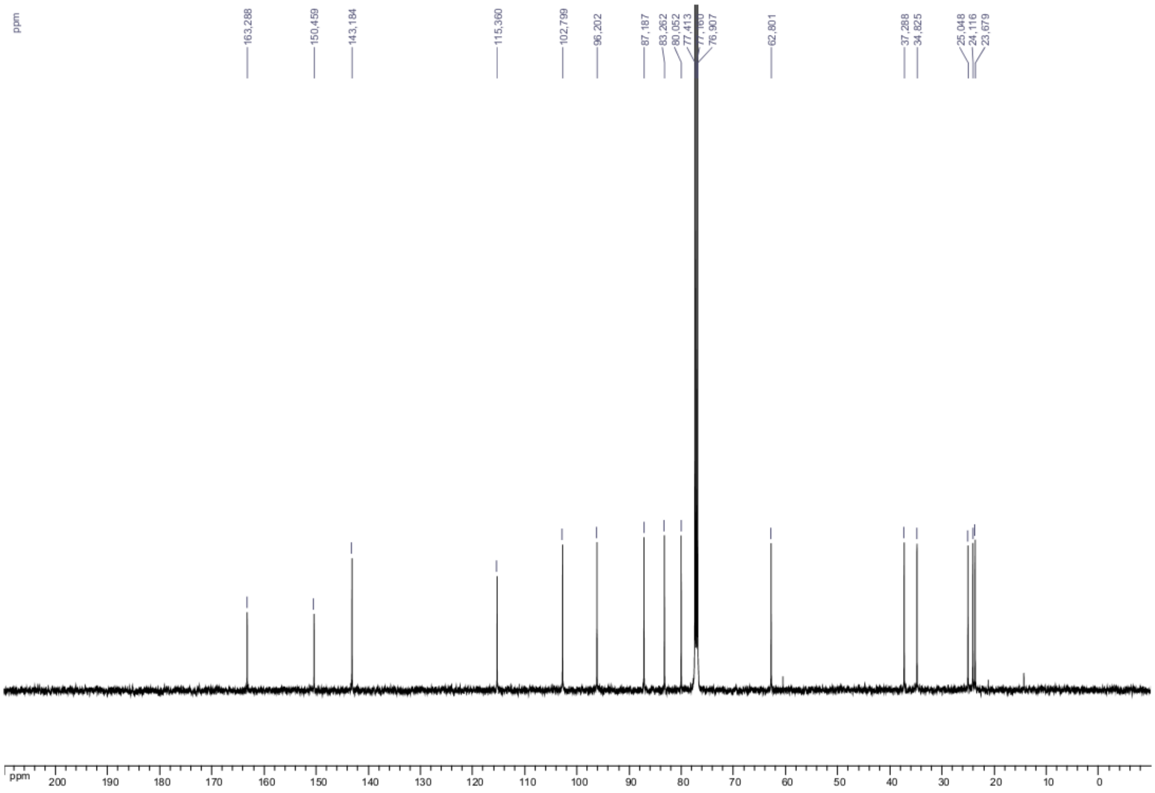

^{1}H NMR, compound **4b**

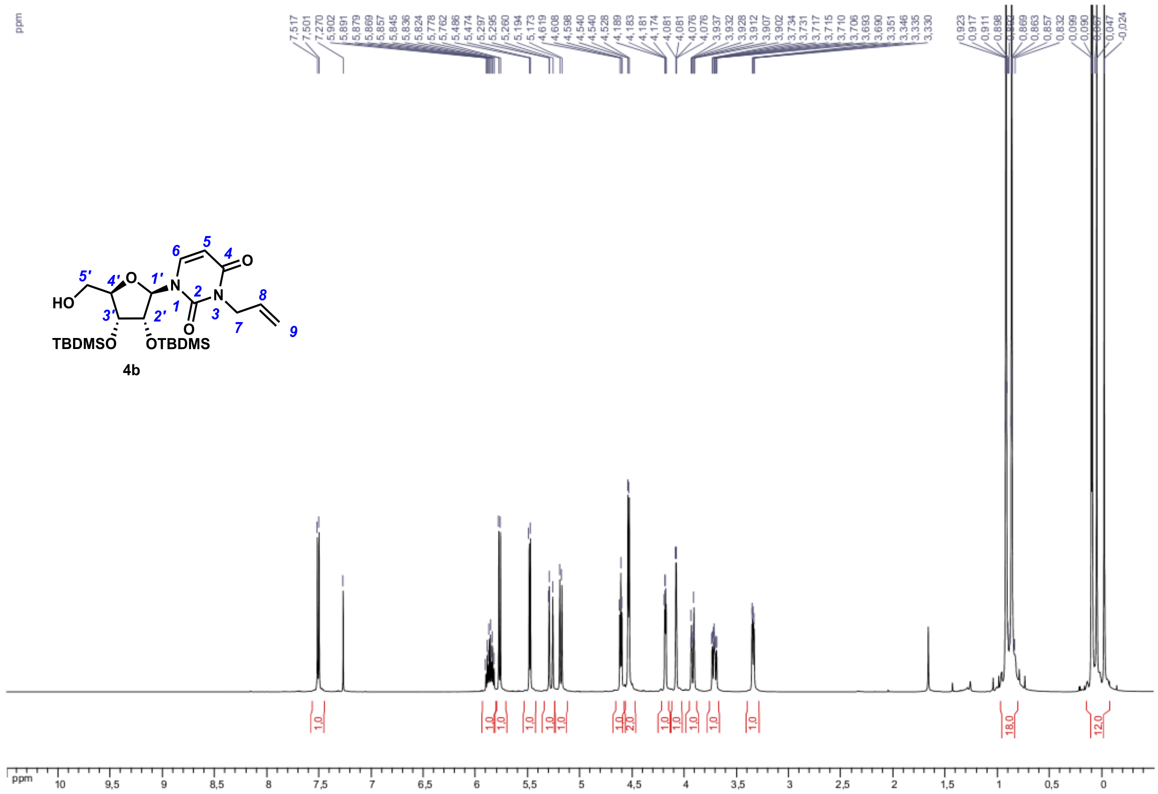

^{13}C NMR

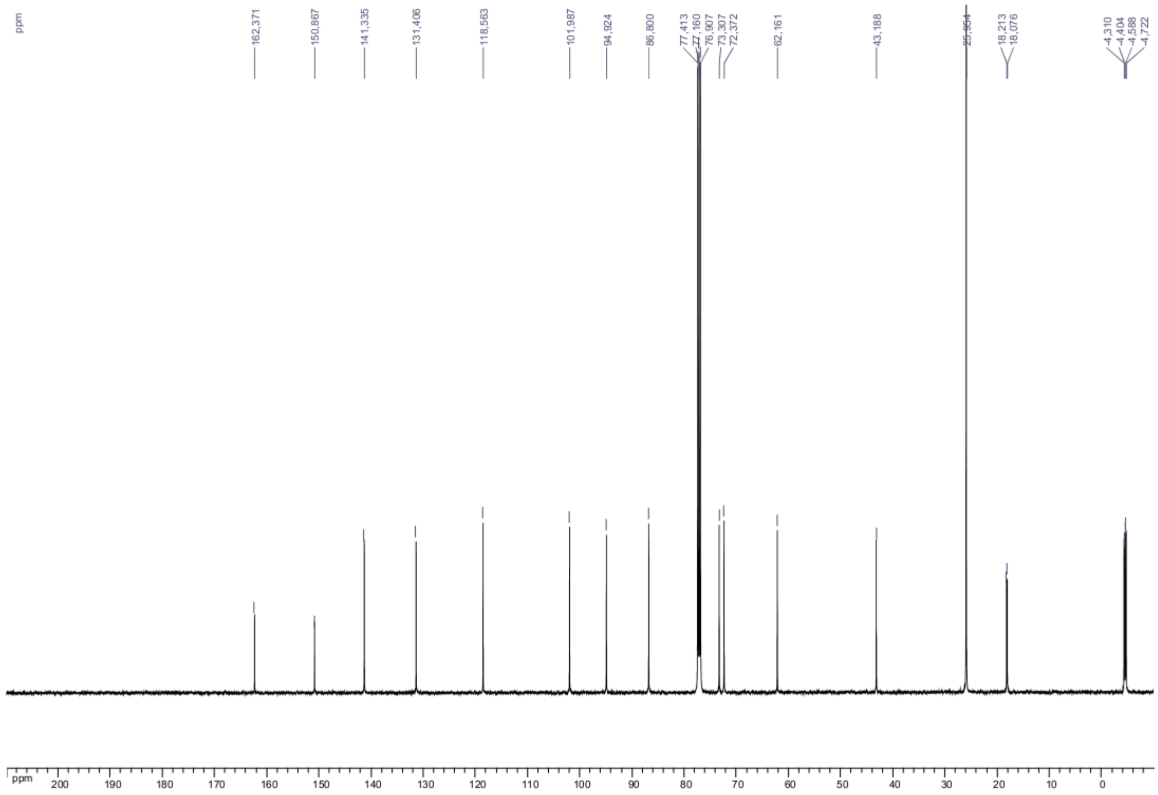

^{1}H NMR, compound **5b**

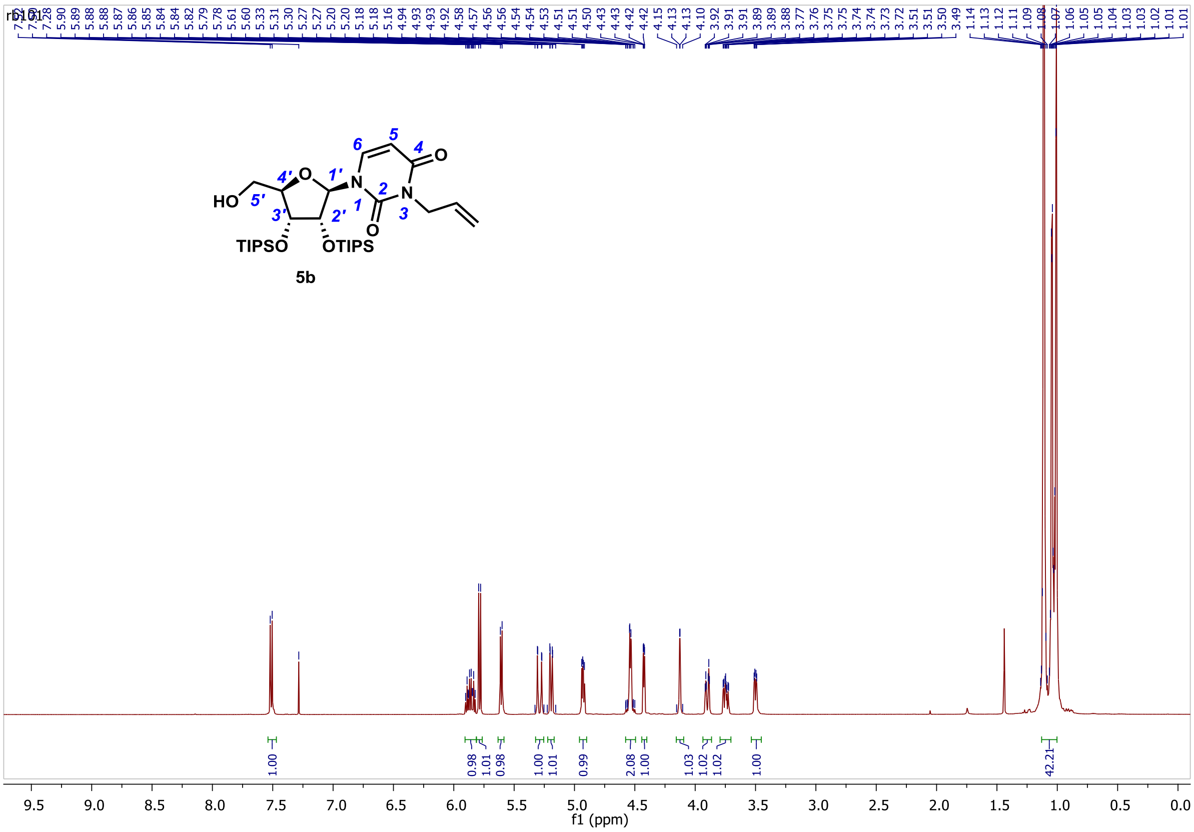

^{13}C NMR

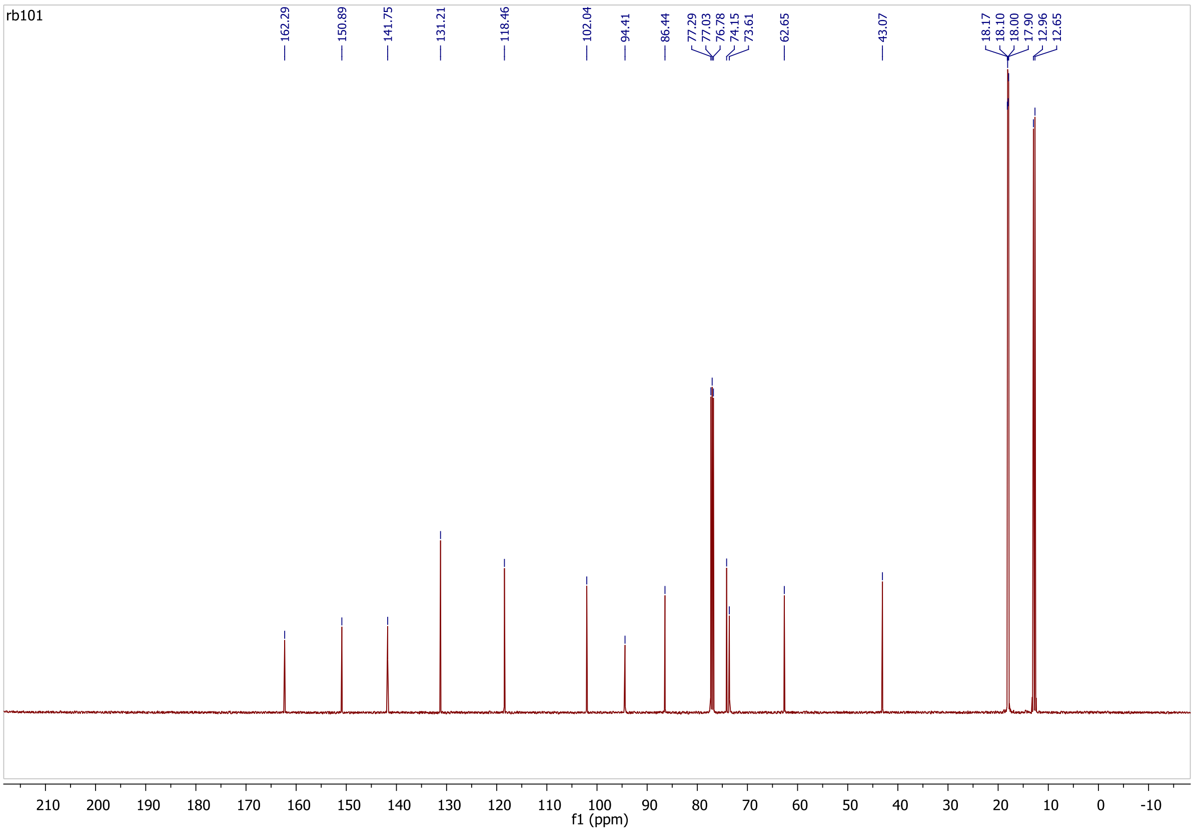

^{1}H NMR, compound **6b**

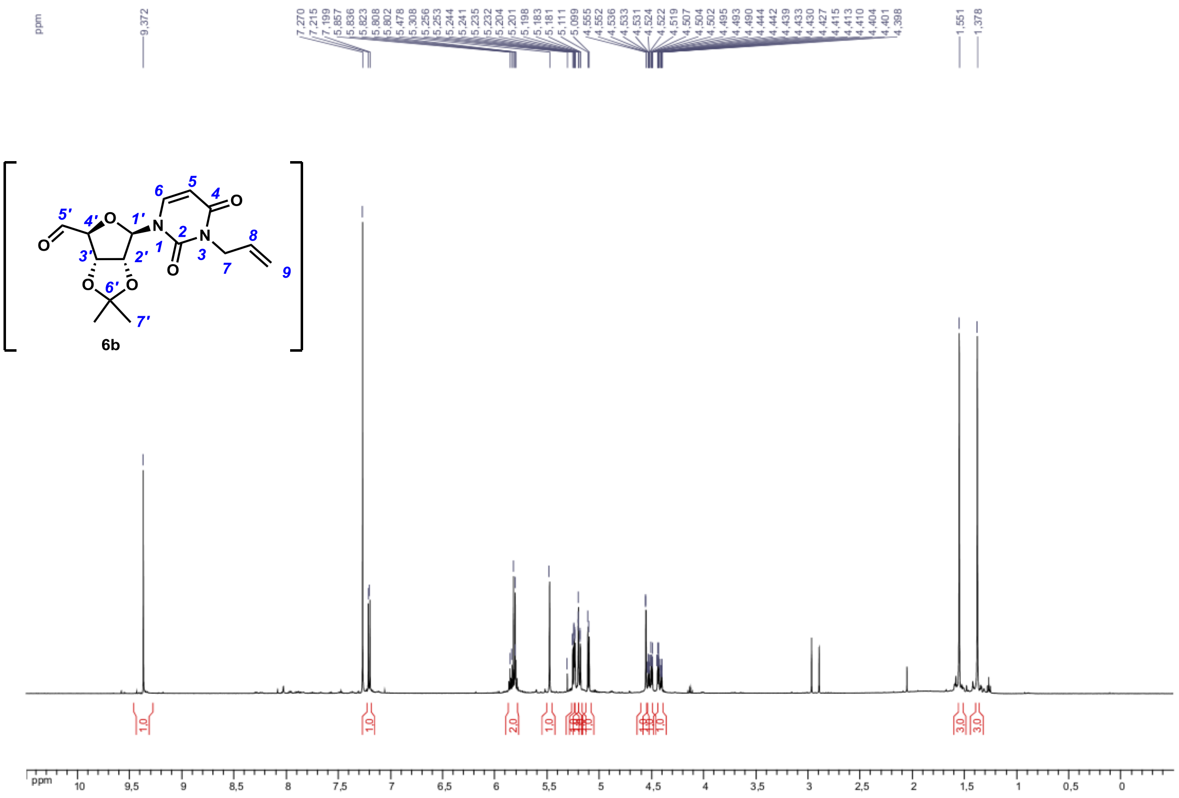

^{13}C NMR

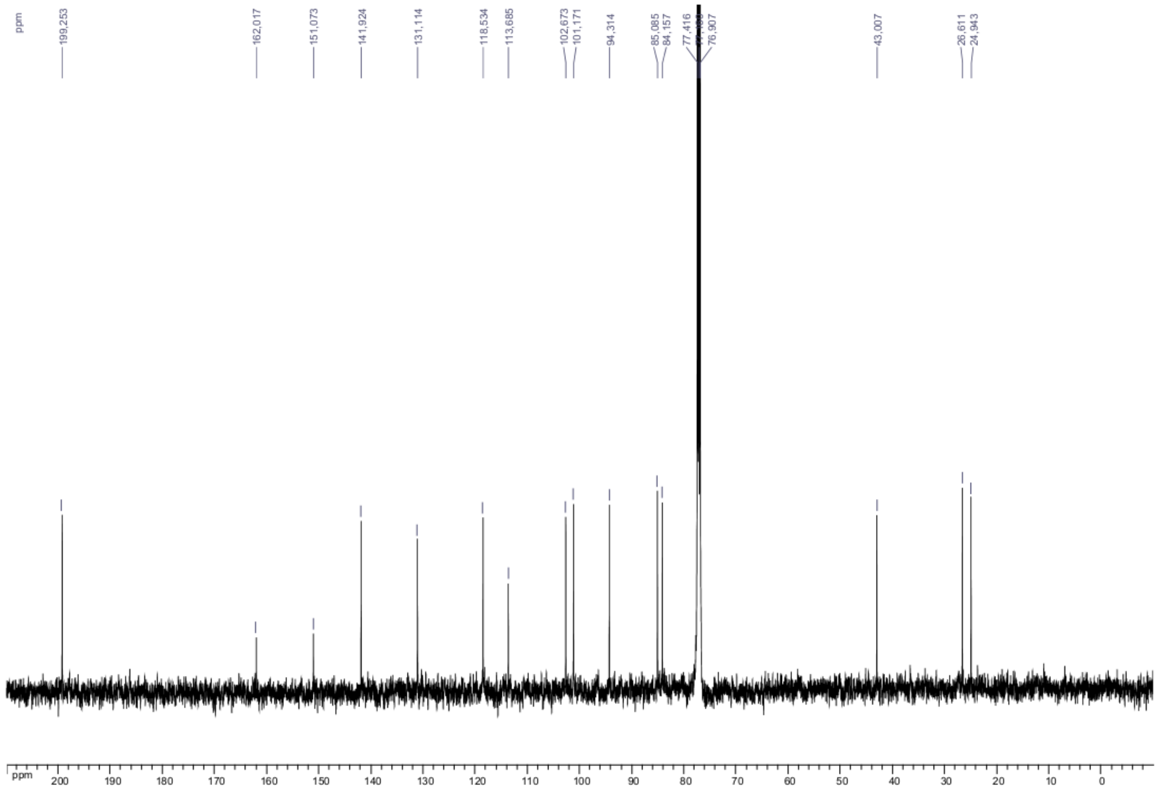

^{1}H NMR, compound **(5’*R*)-11bb**

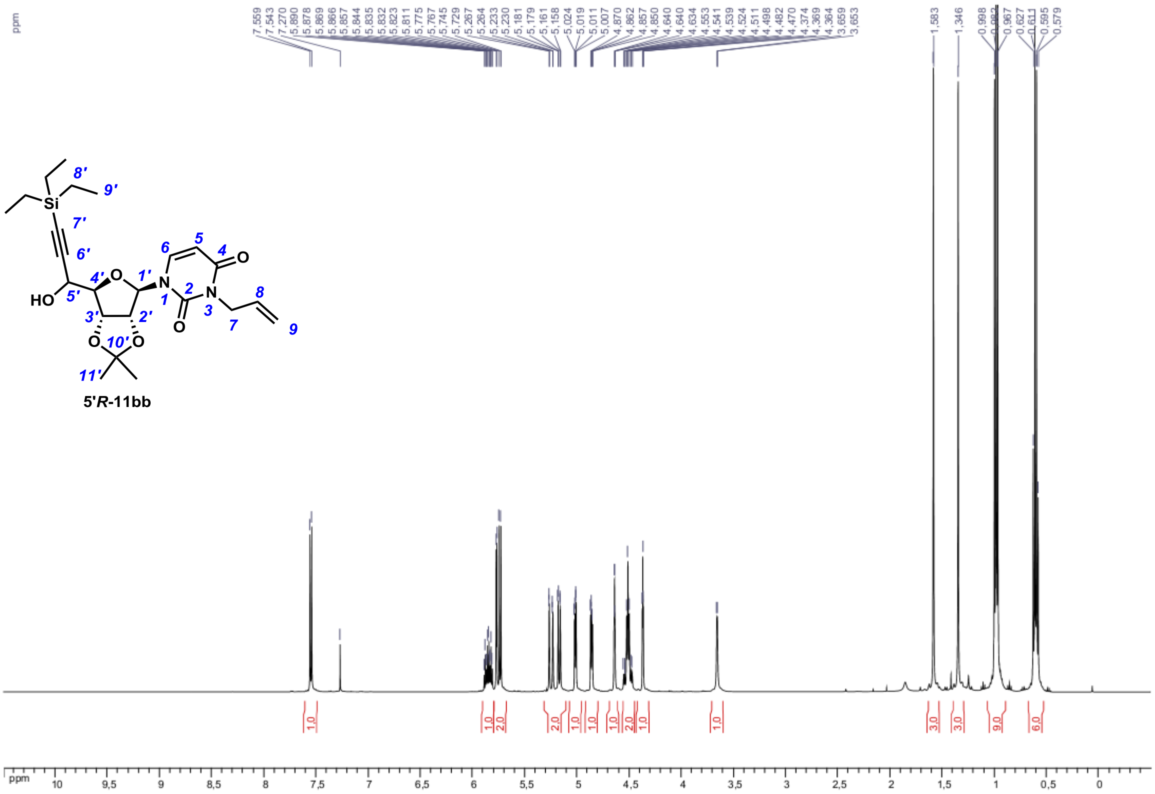

5'*R*-11bb

^{13}C NMR

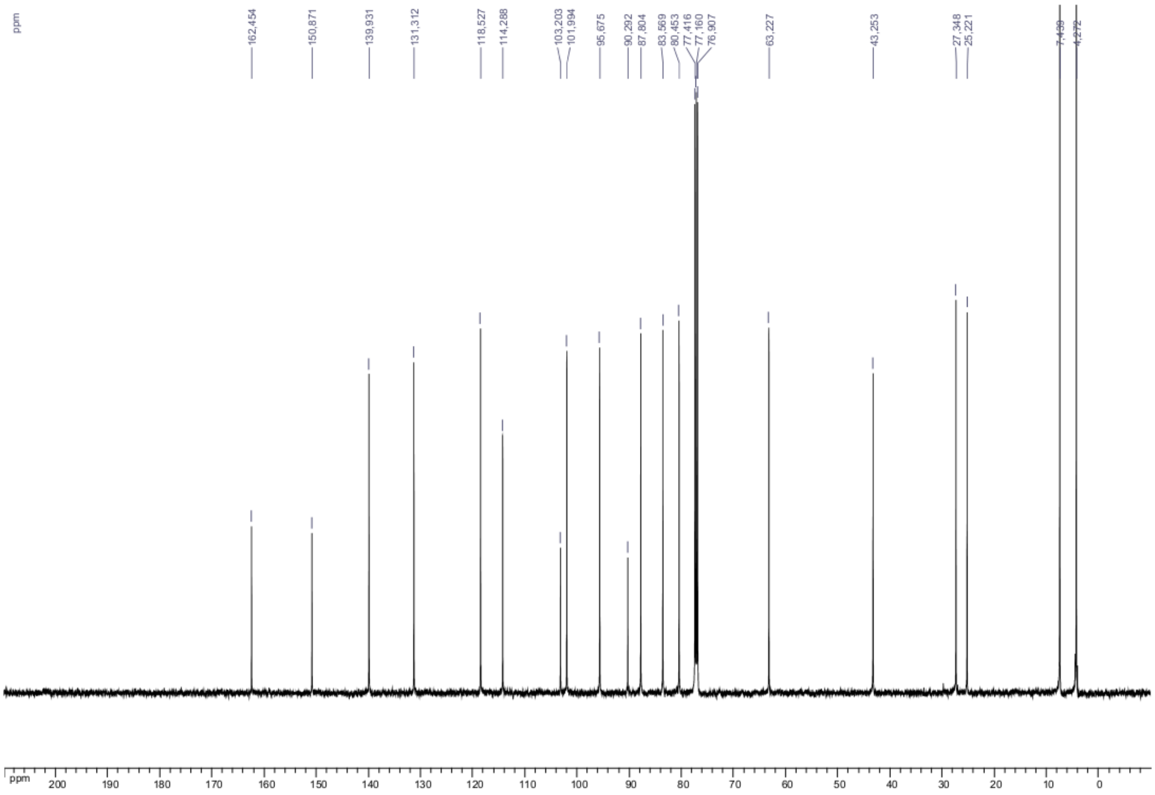

^{1}H NMR, compound 7**a**

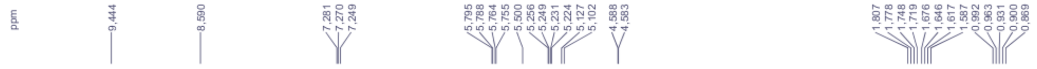

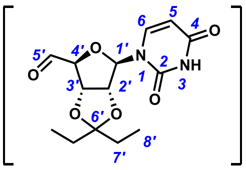

7a

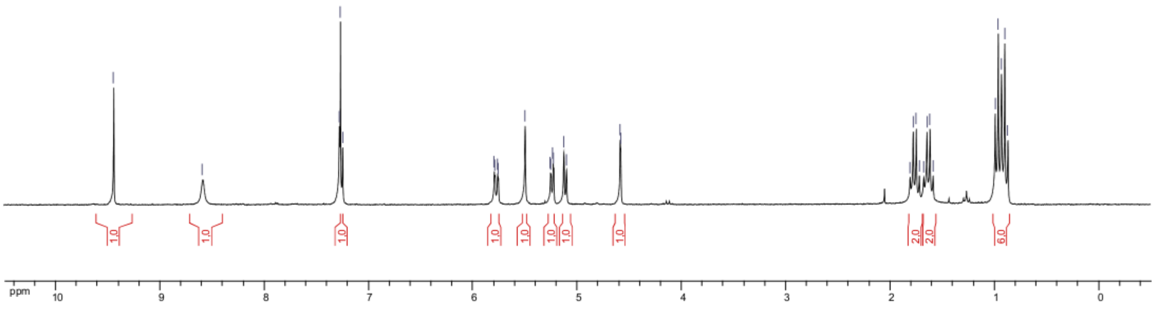

^{13}C NMR

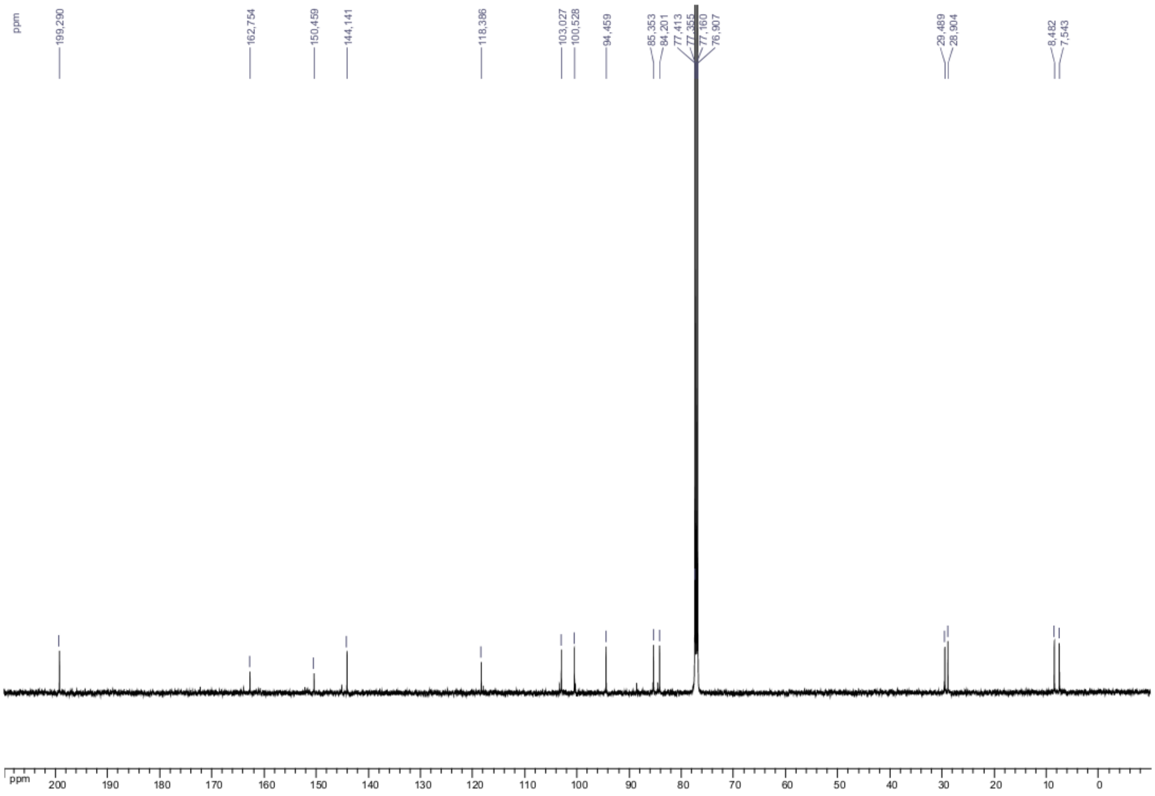

^{1}H NMR, compound **5’*R*-12ab**

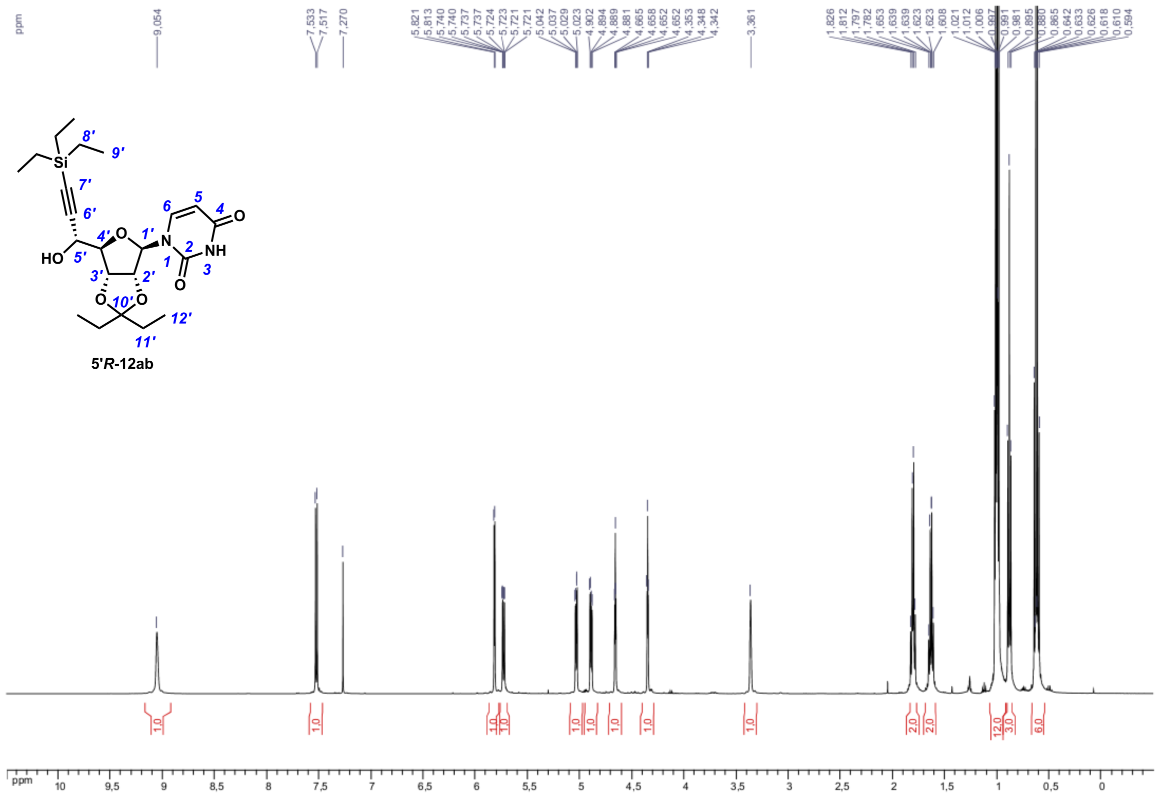

^{13}C NMR

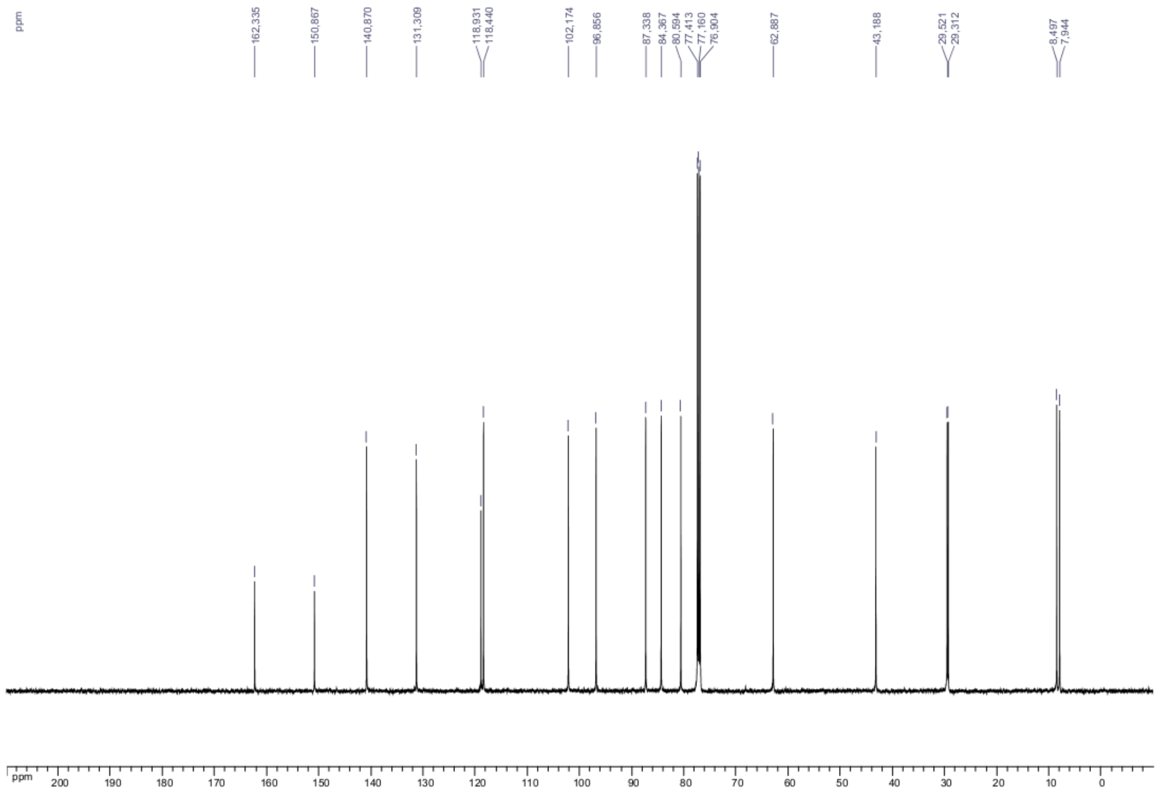

^{1}H NMR, compound **8a**

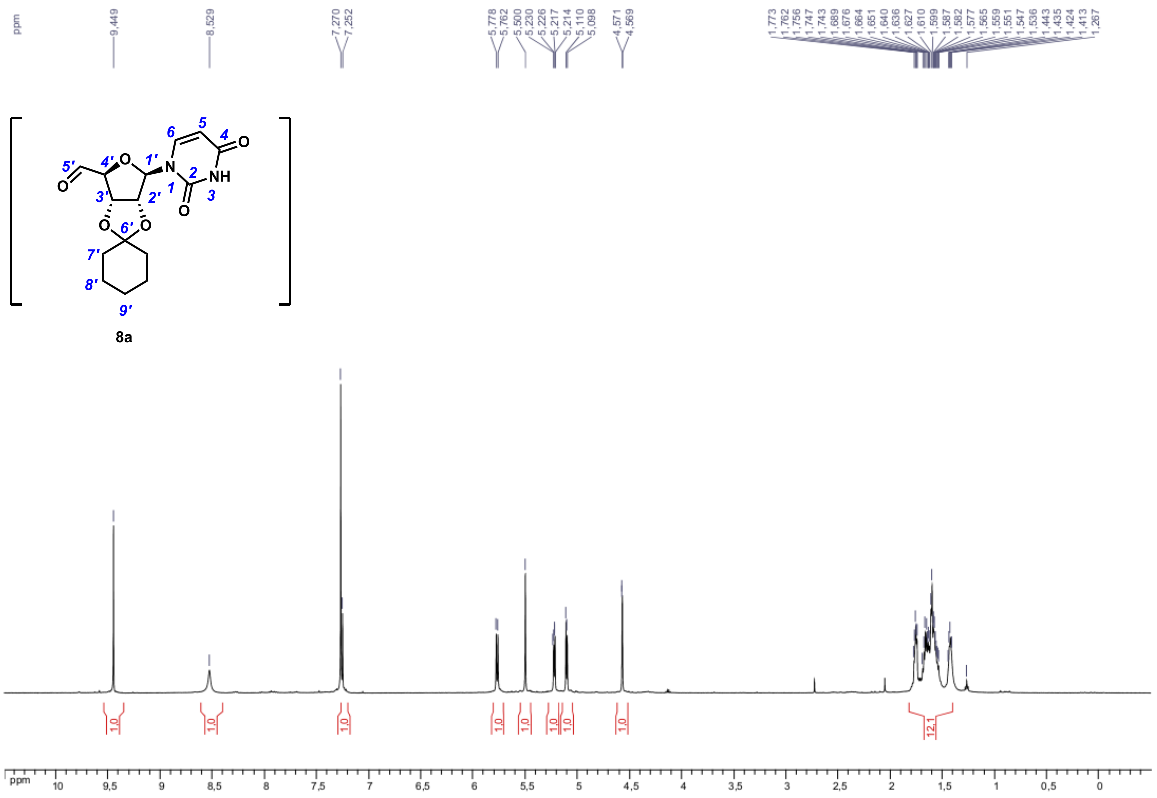

^{13}C NMR

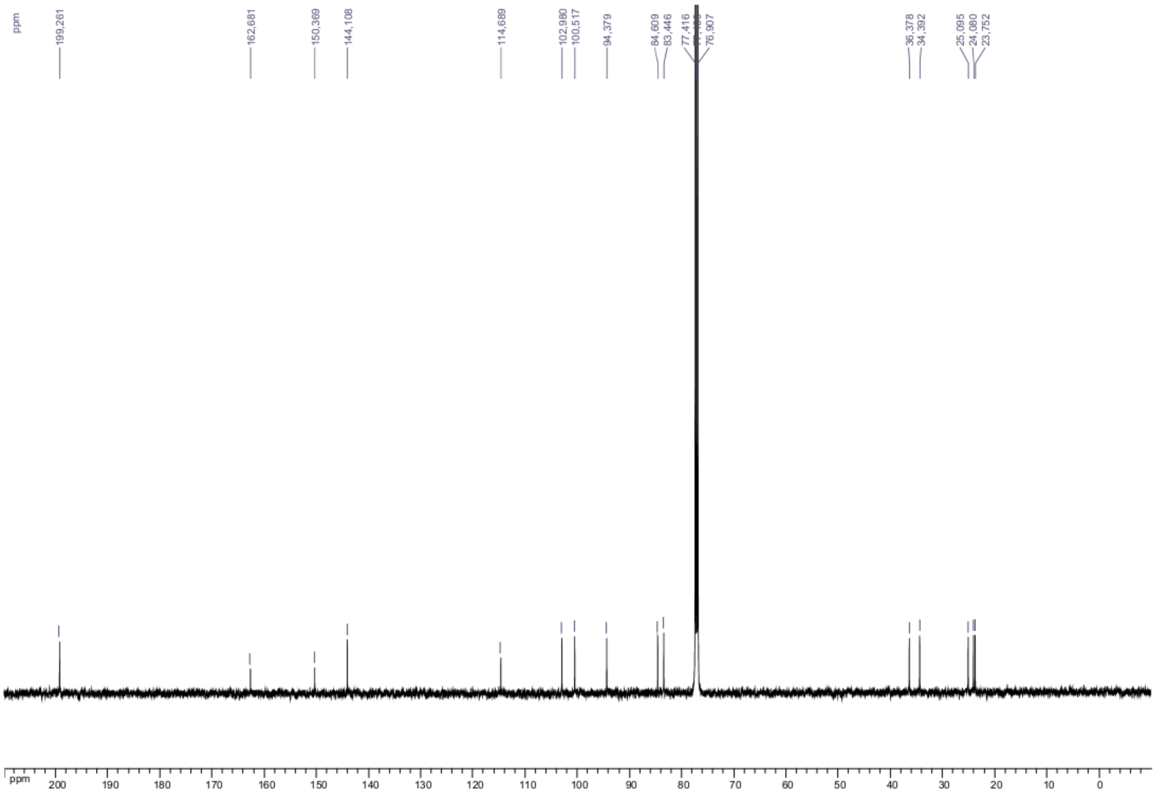

^{1}H NMR, compound **5’*R*-13ab**

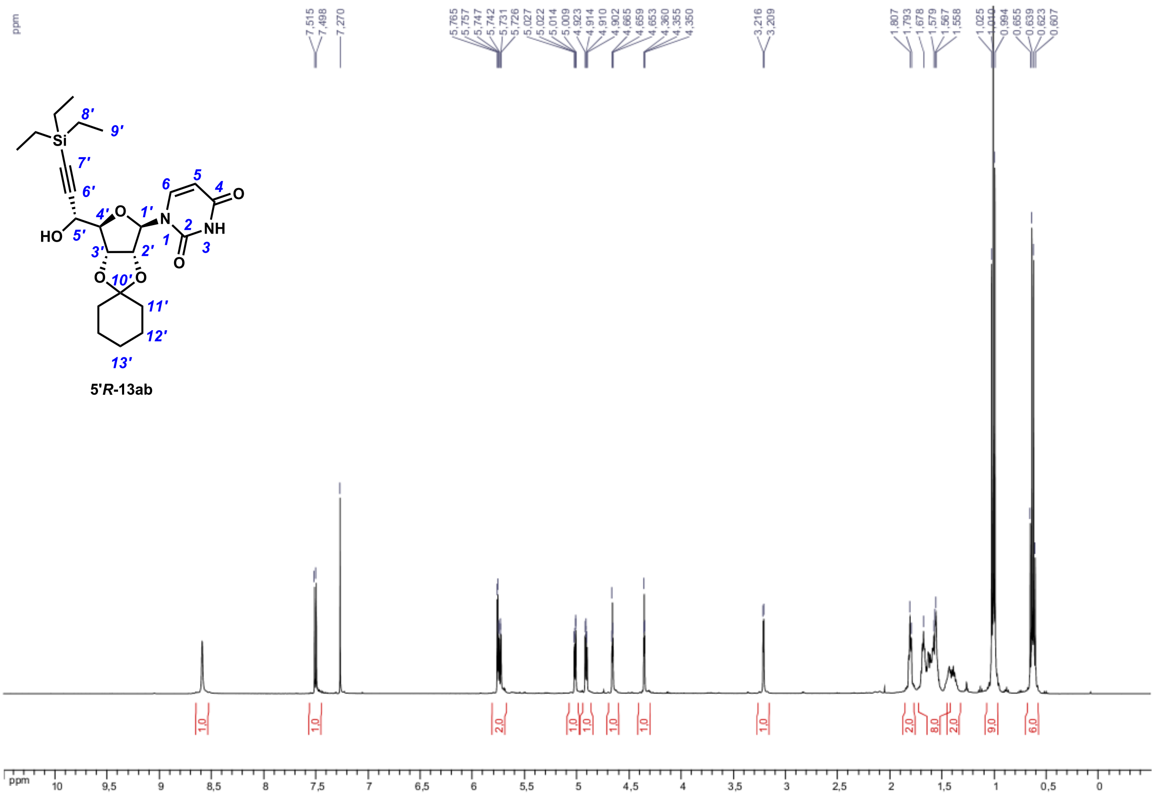

^{13}C NMR

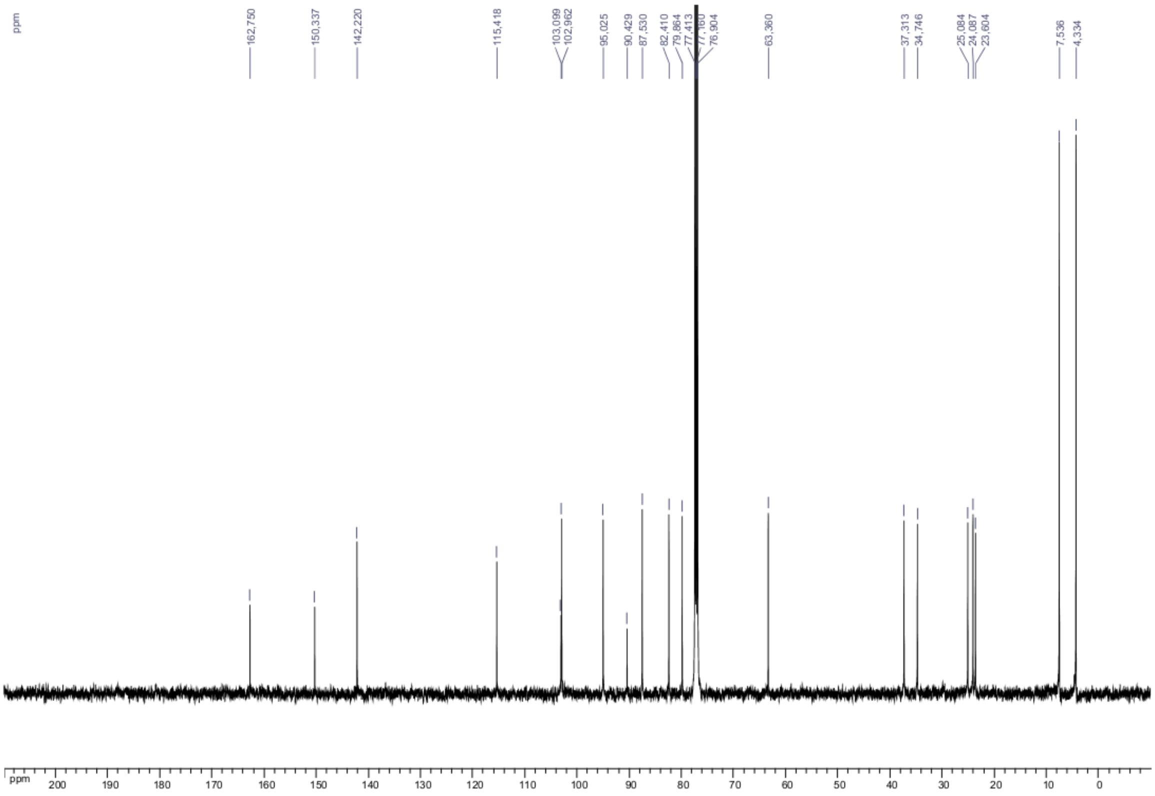

^{1}H NMR, compound **5’*S*-14ab**

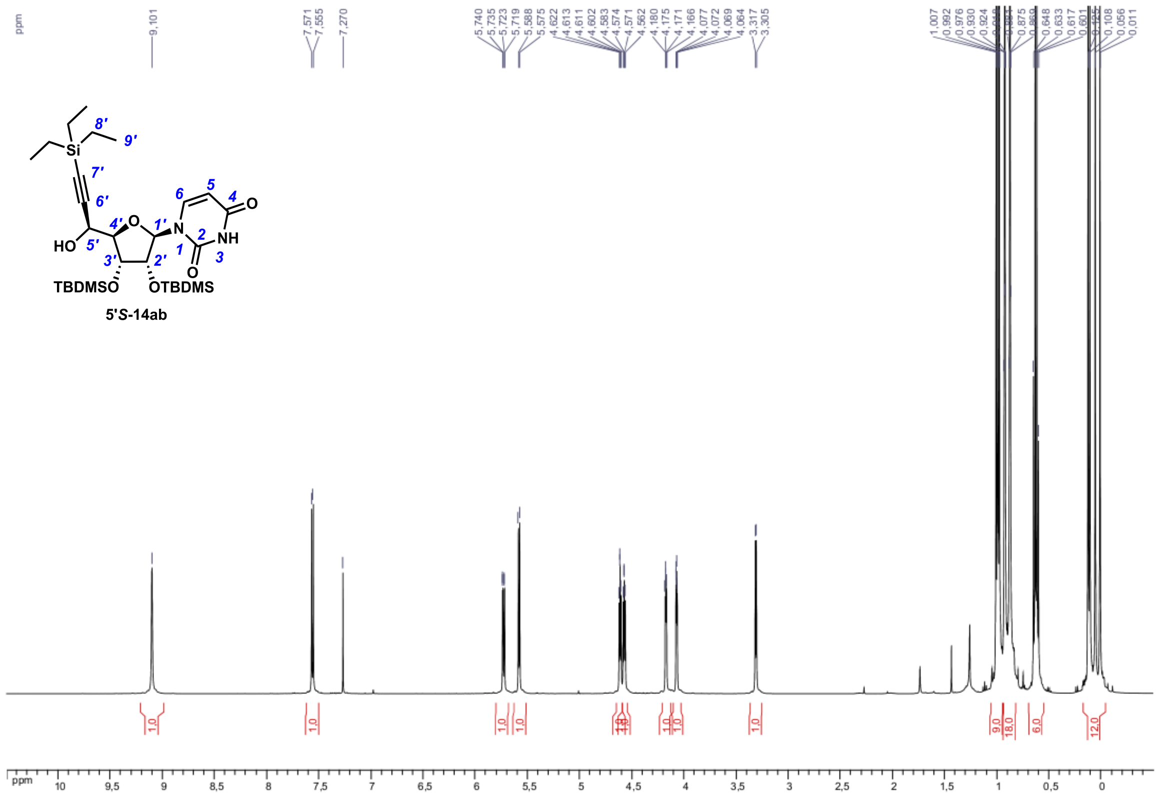

^{13}C NMR

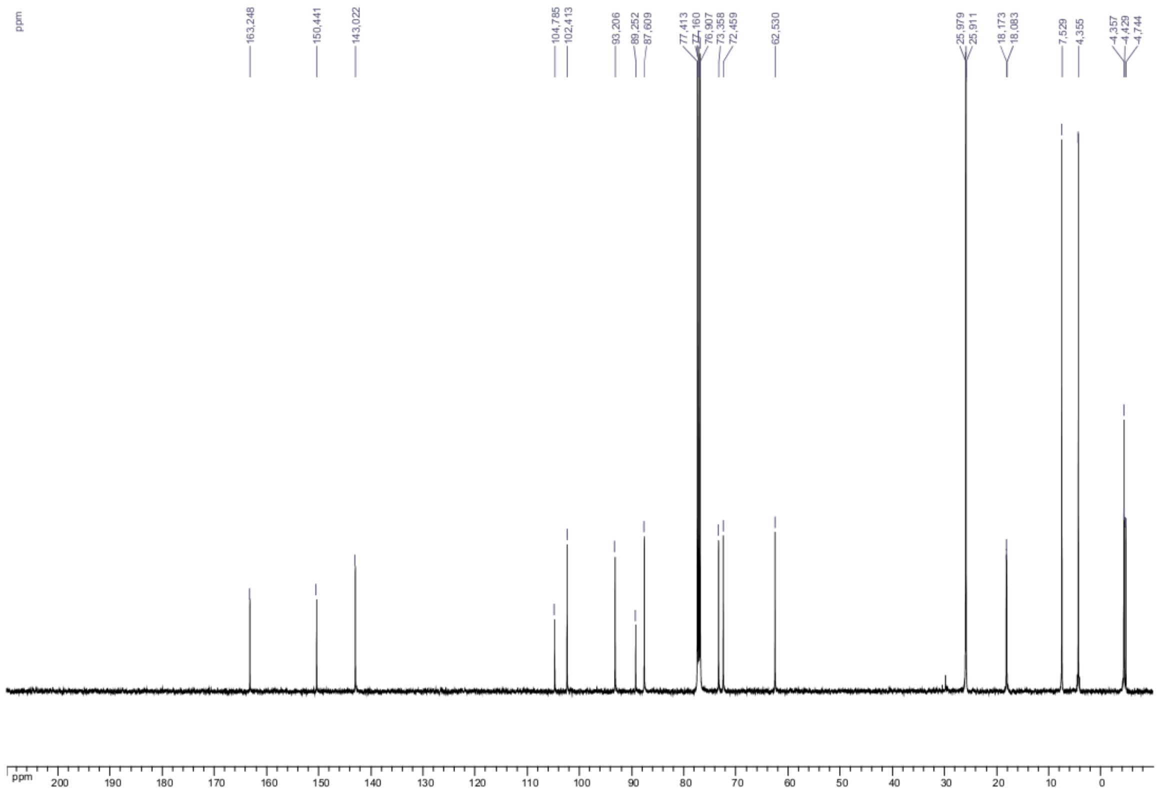

^{1}H NMR, compound **9b**

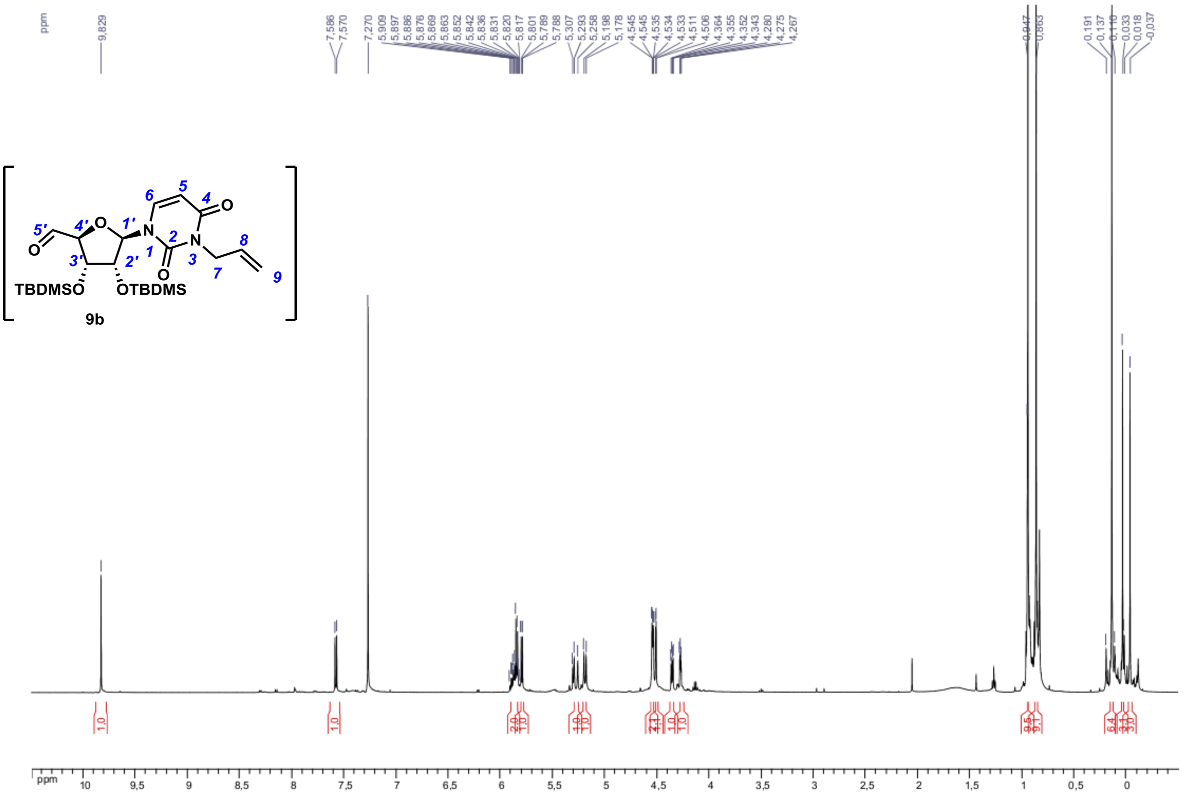

^{13}C NMR

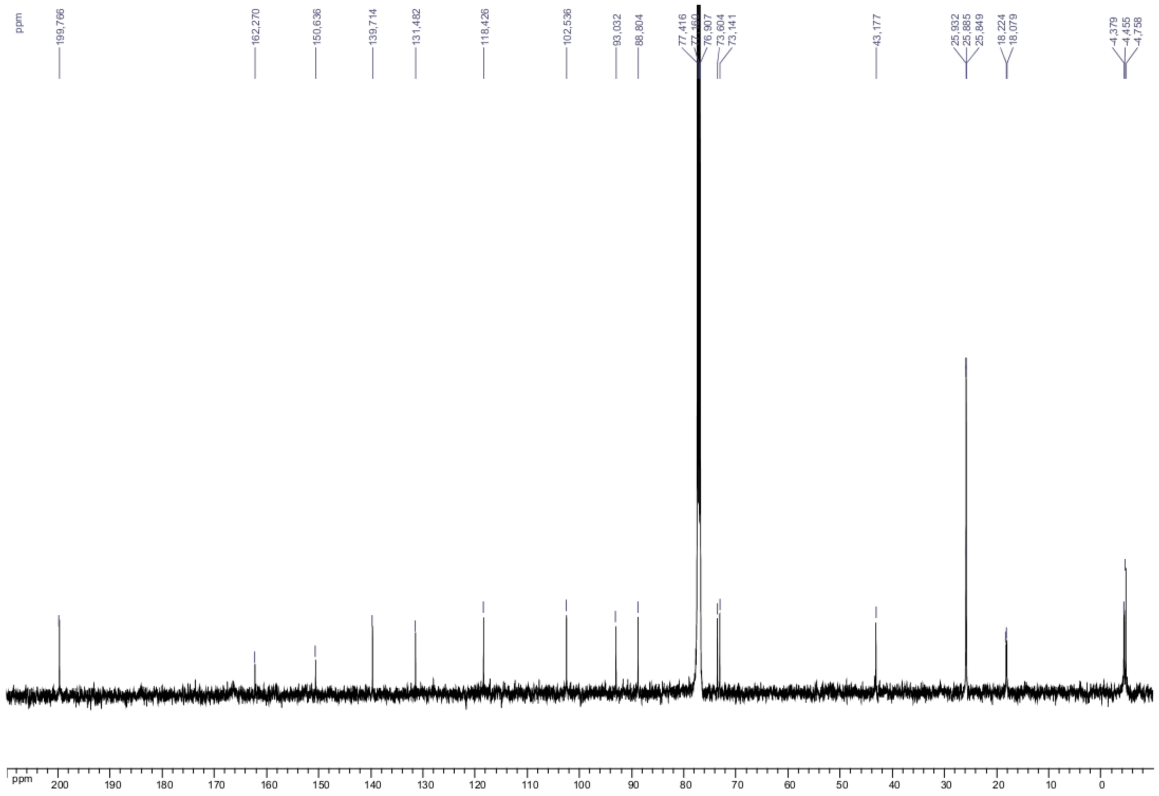

^{1}H NMR, compound **5’*S*-14bb**

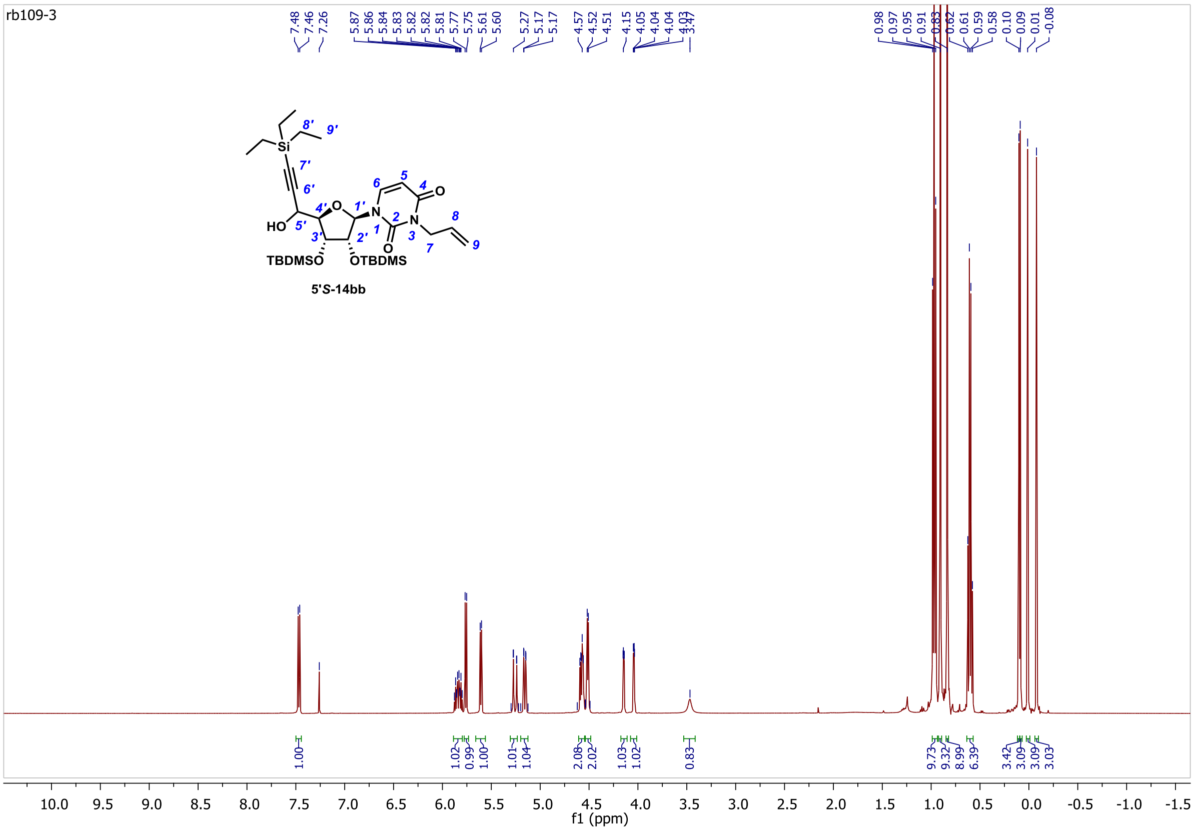

^{13}C NMR

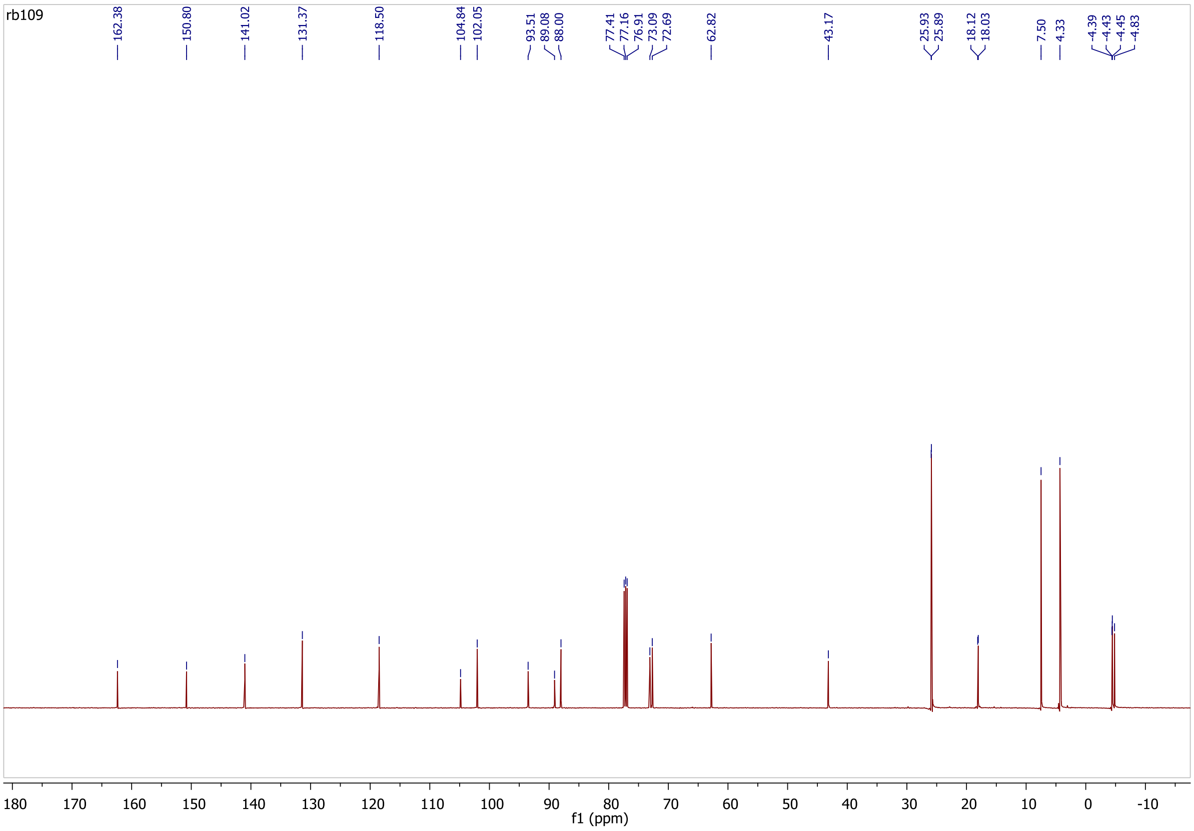

^{1}H NMR, compound **5'*S*-15aa**

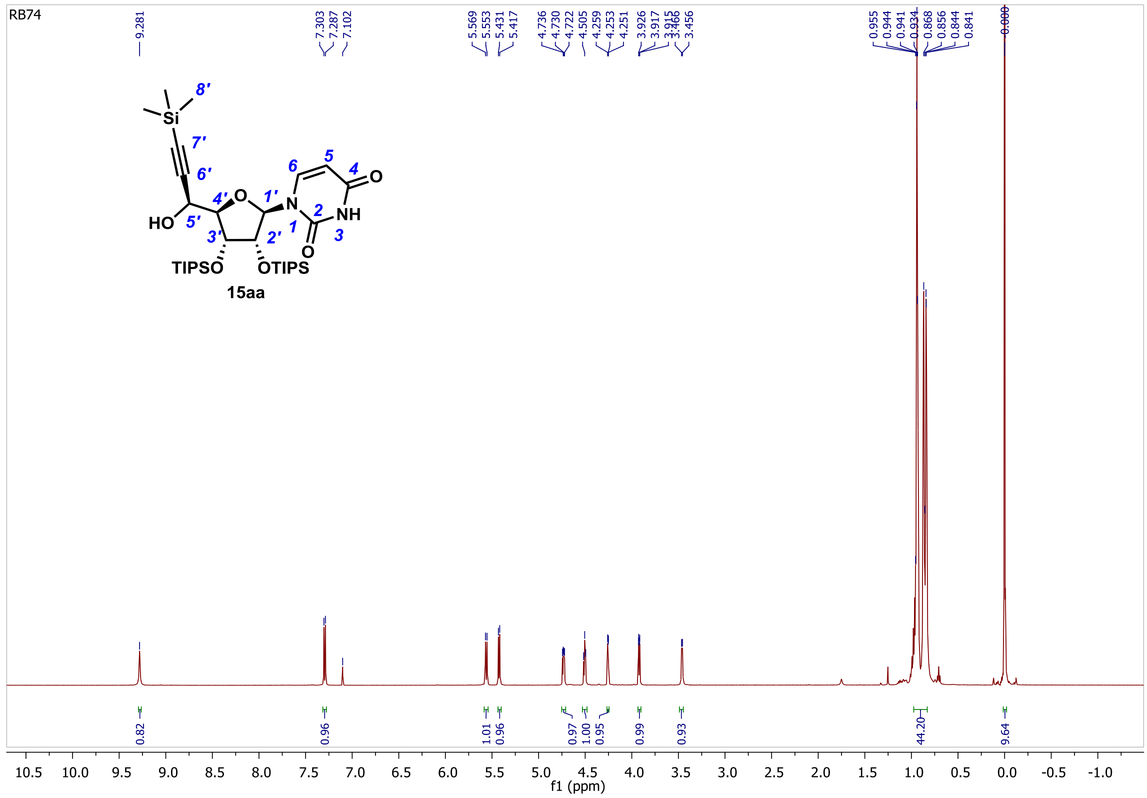

^{13}C NMR

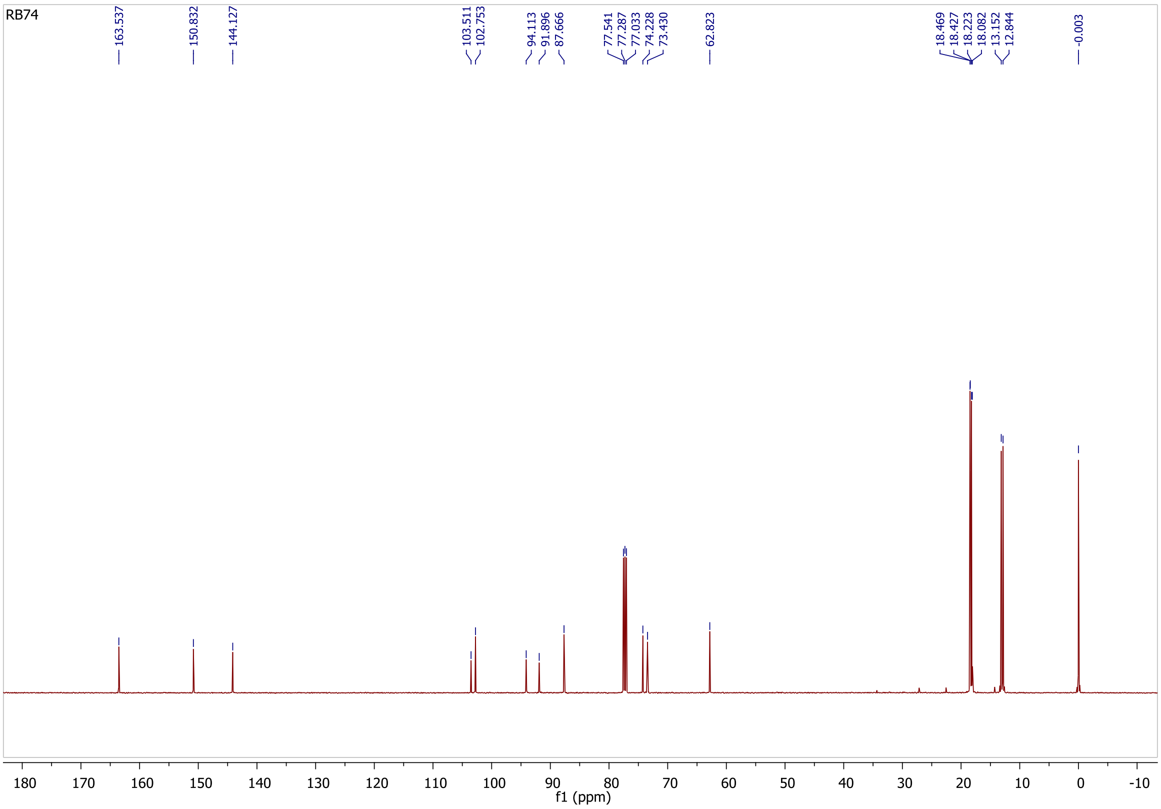

^{1}H NMR, compound **5’*S*-15ab**

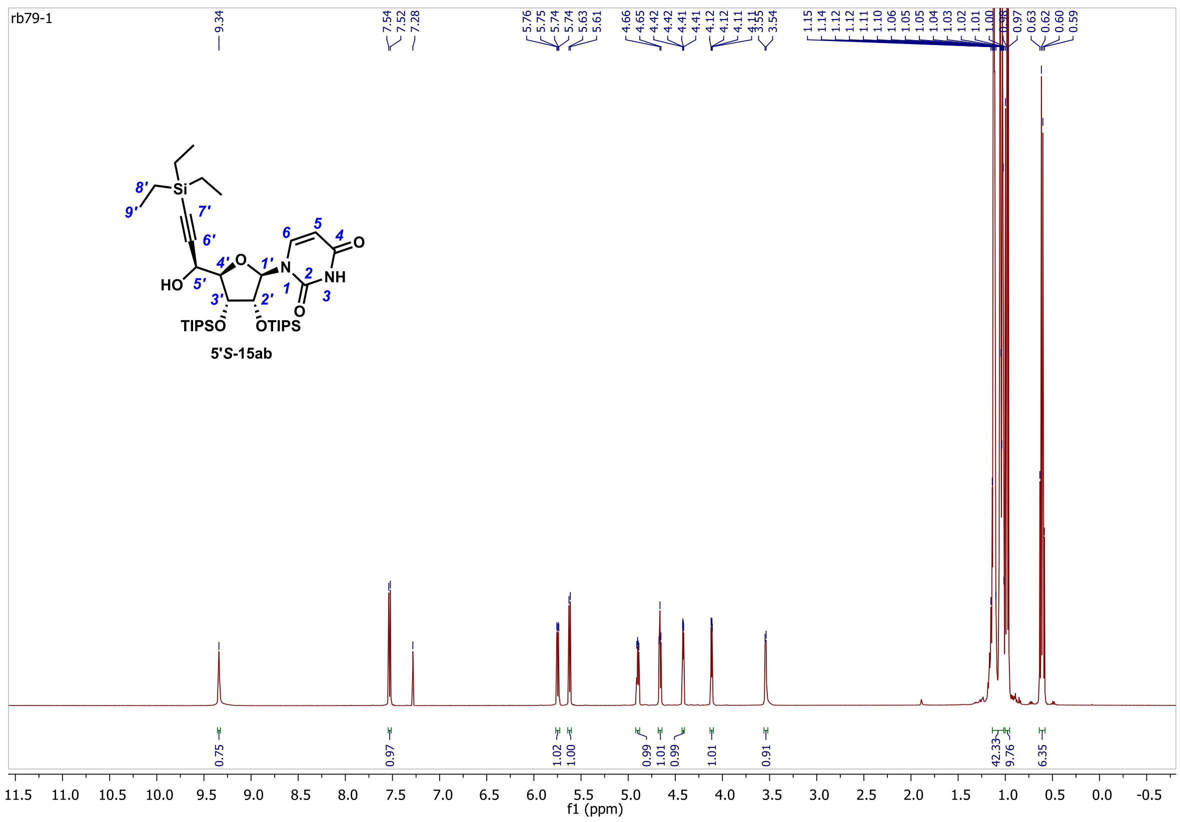

^{13}C NMR

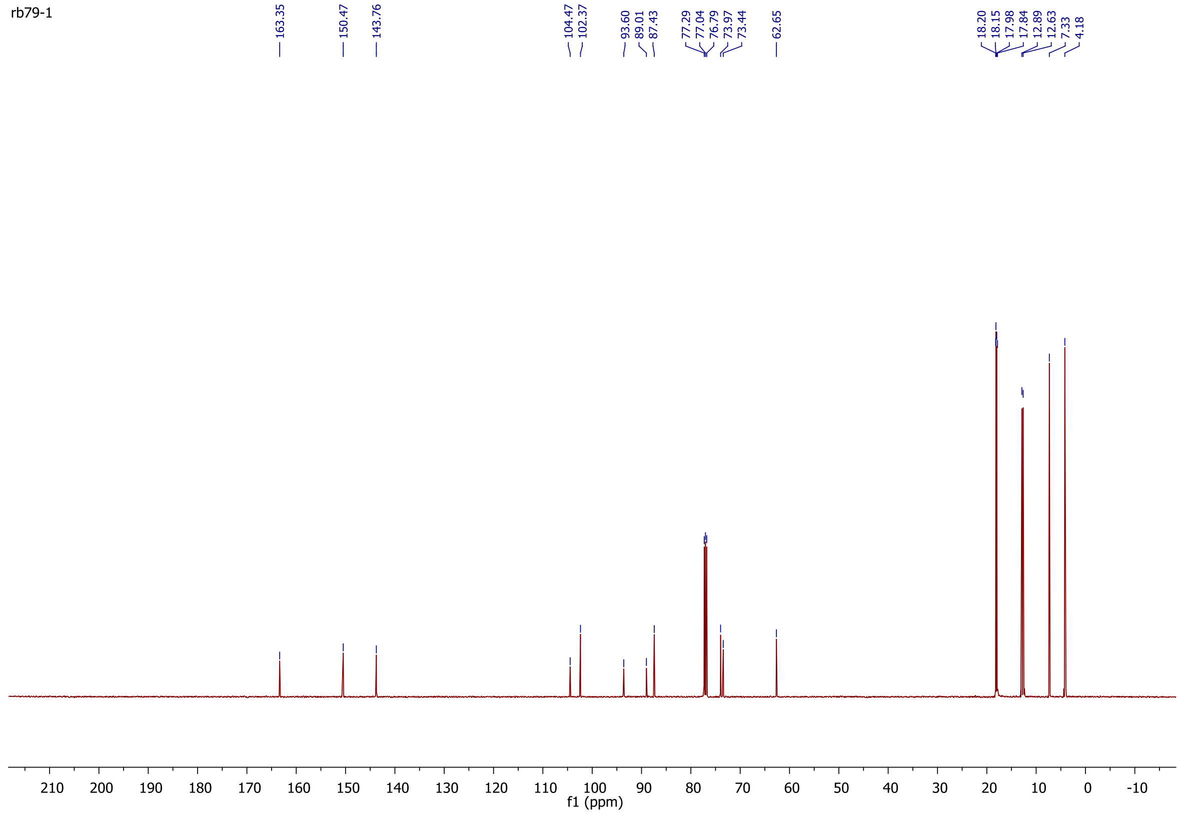

^{1}H NMR, compound **5’*S*-15ac**

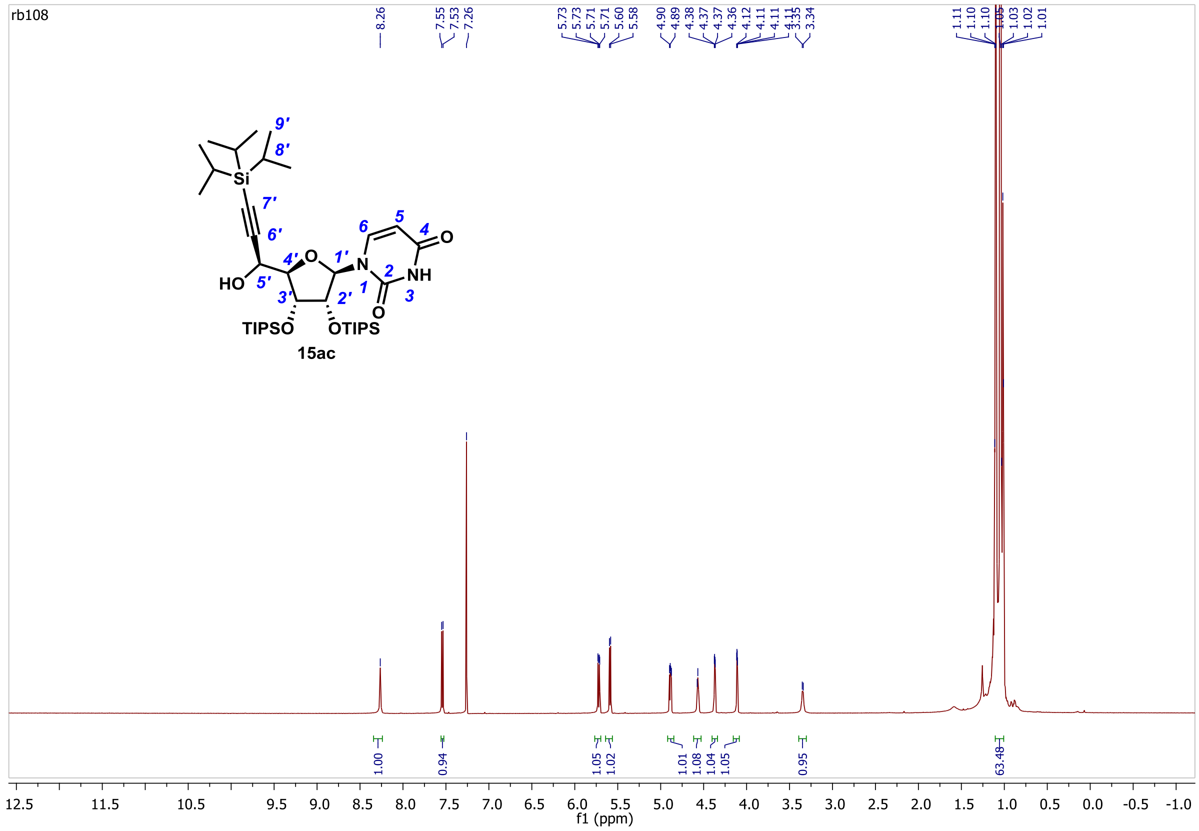

^{13}C NMR

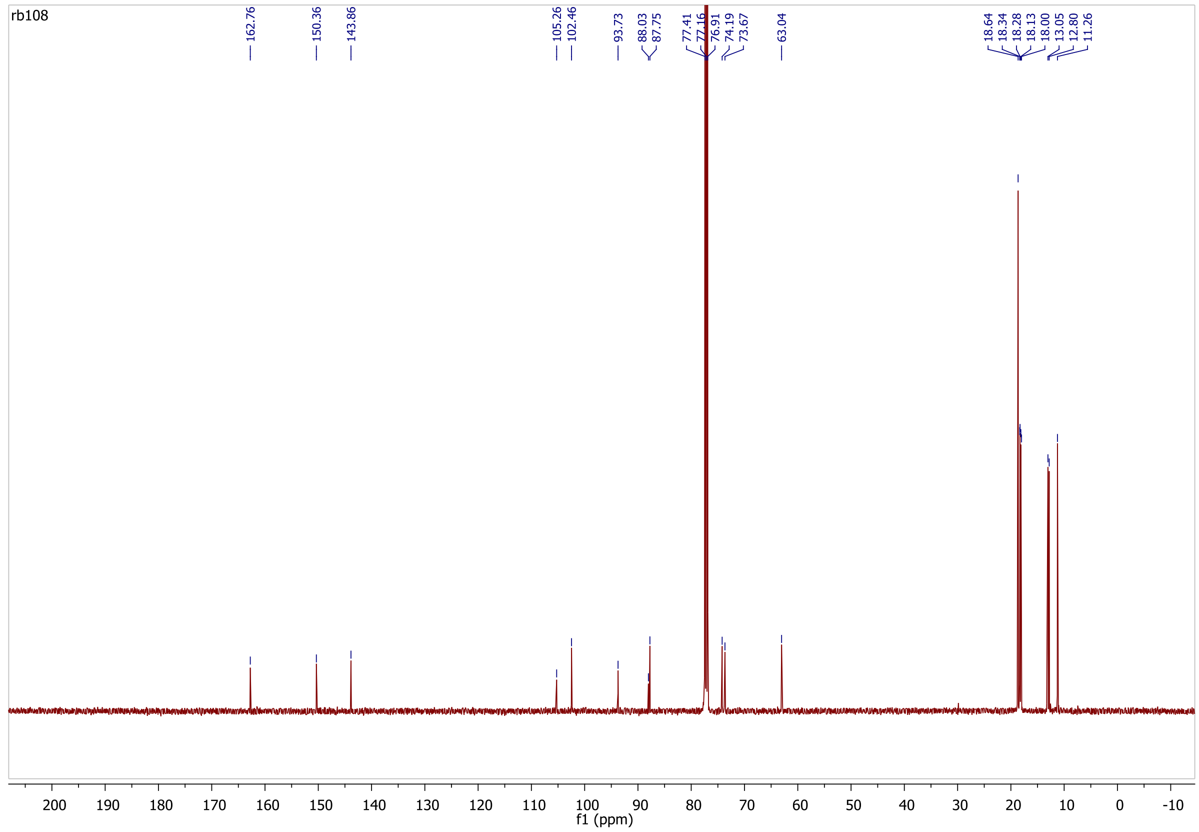

^{1}H NMR, compound **5’*S*-15bc**

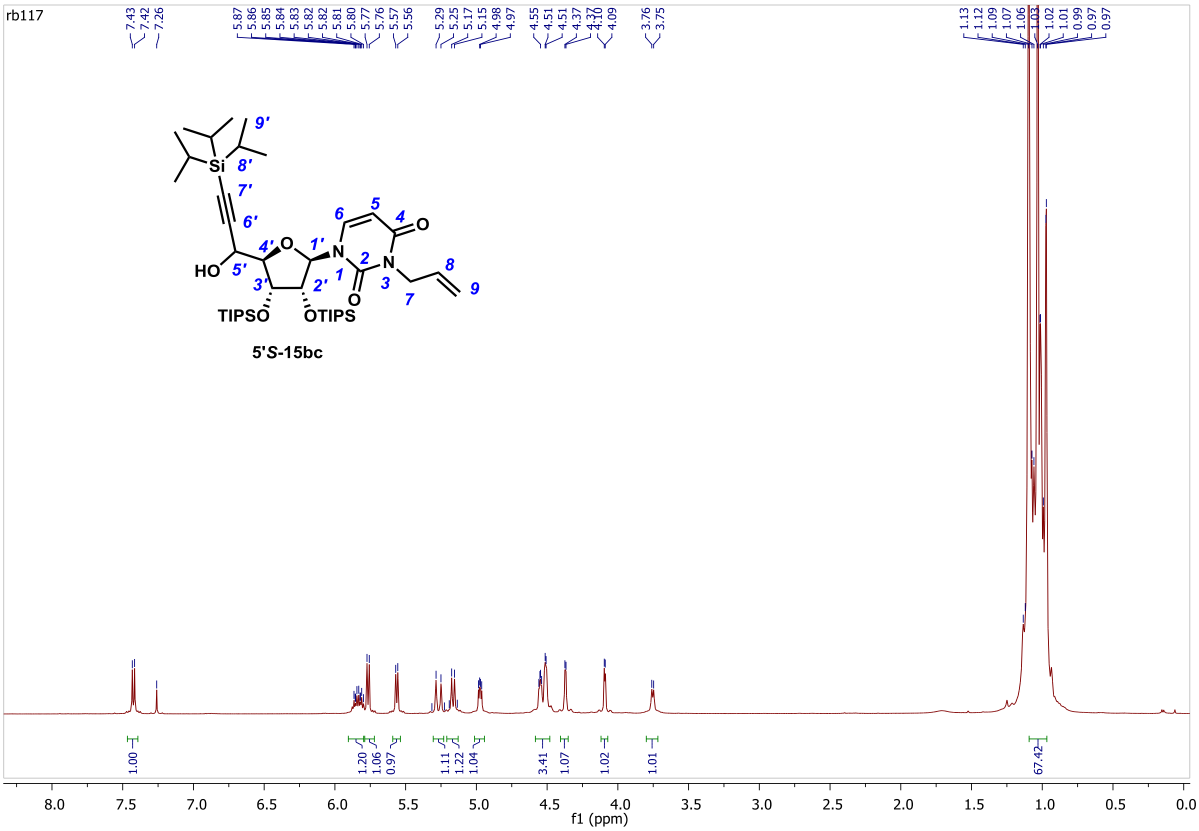

^{13}C NMR

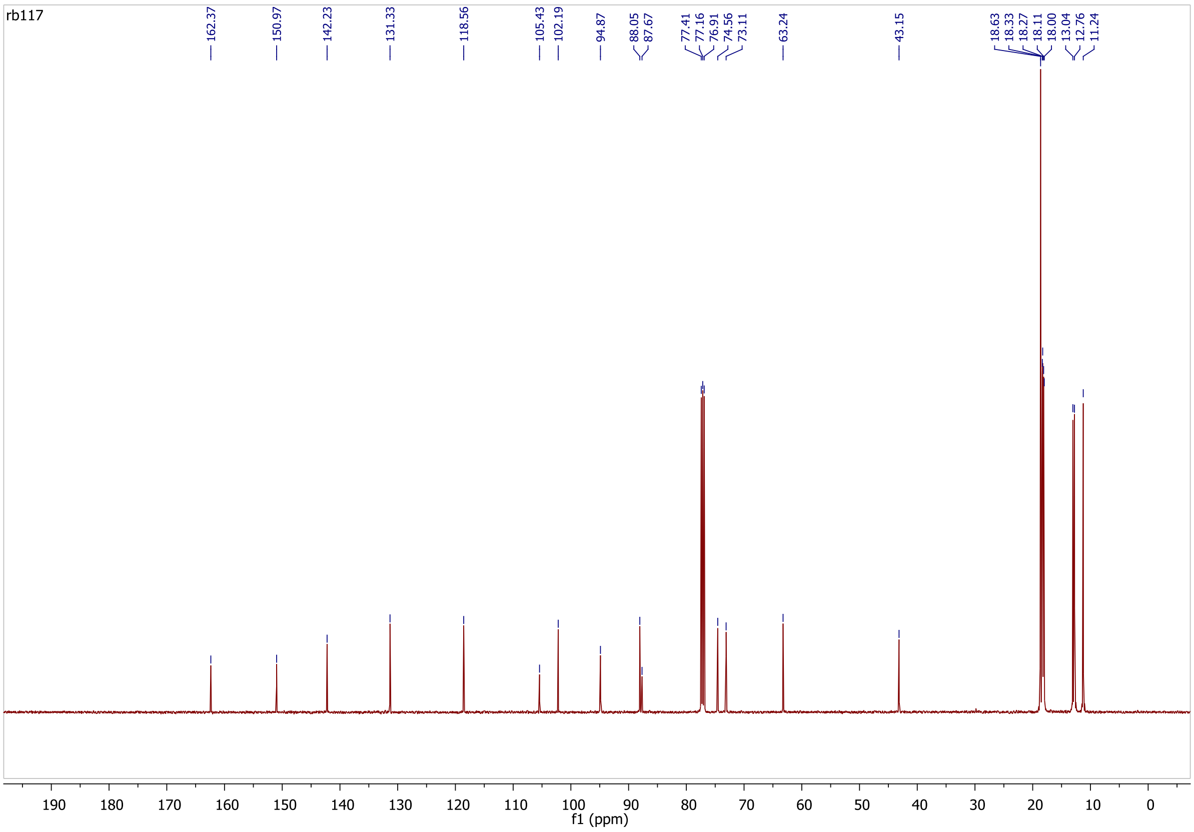

^{1}H NMR, 500 MHz (CD_3OD), compound **5’*R*-16**

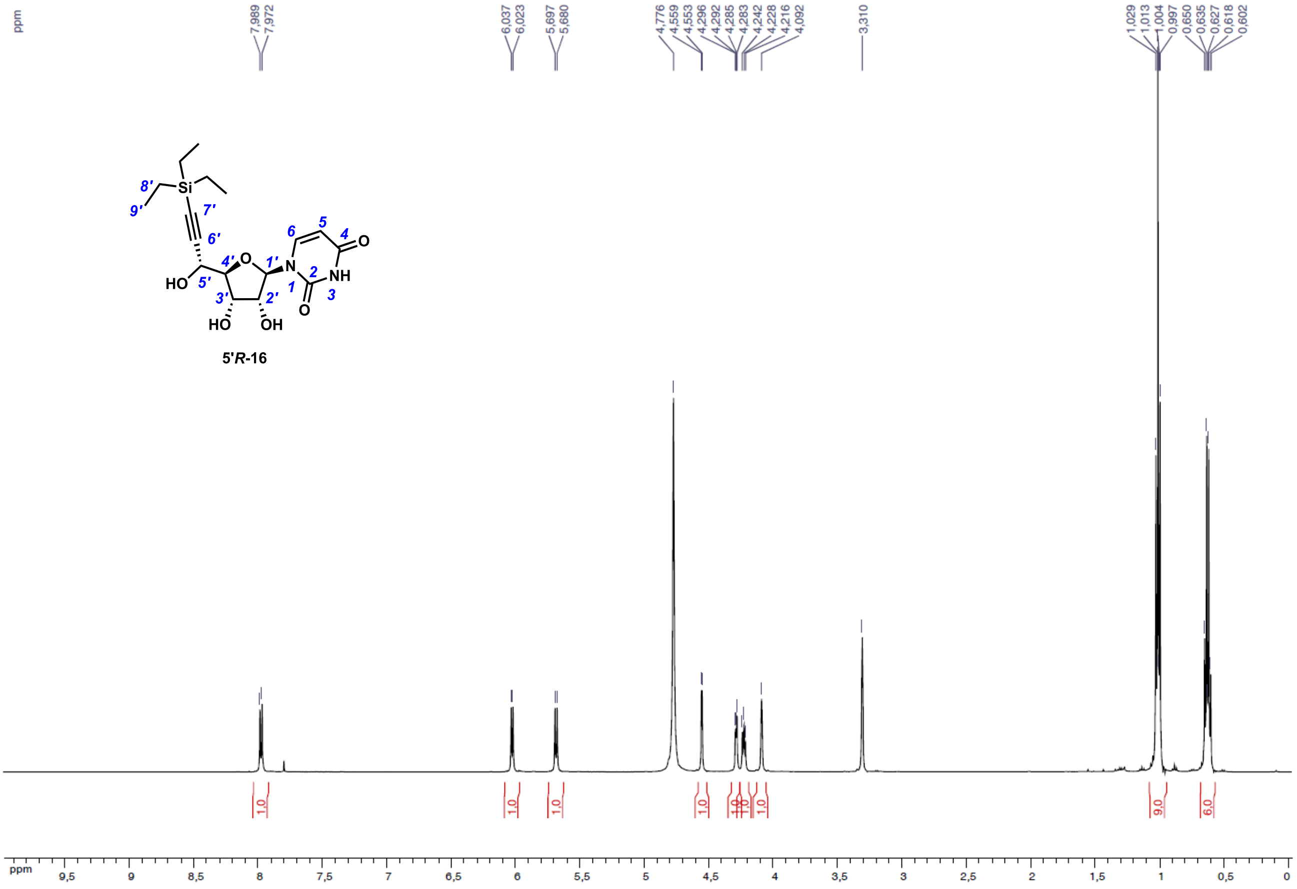

^{13}C NMR, 125 MHz (CD_3OD)

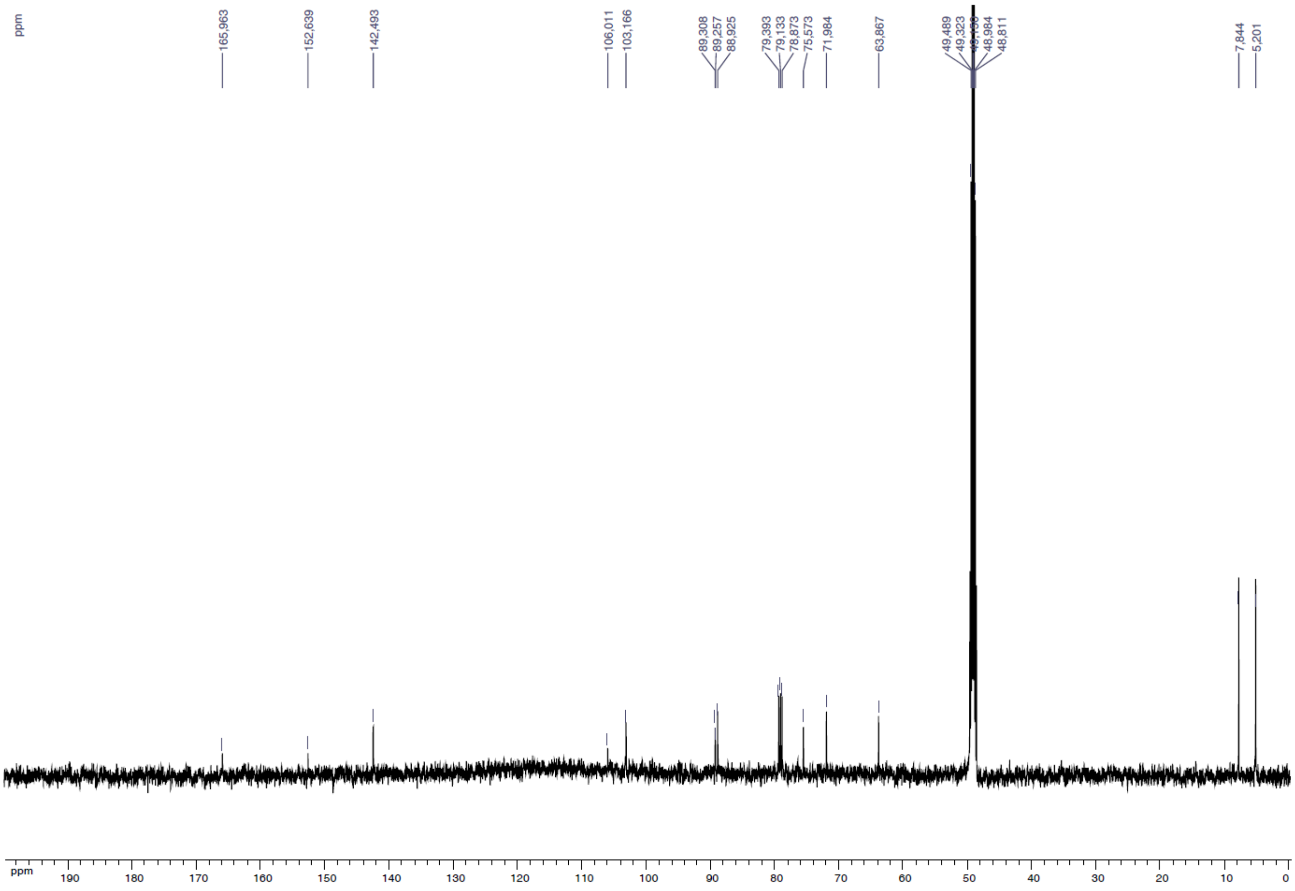

^{1}H NMR, 500 MHz (CD_3OD, compound **5’*S*-16**

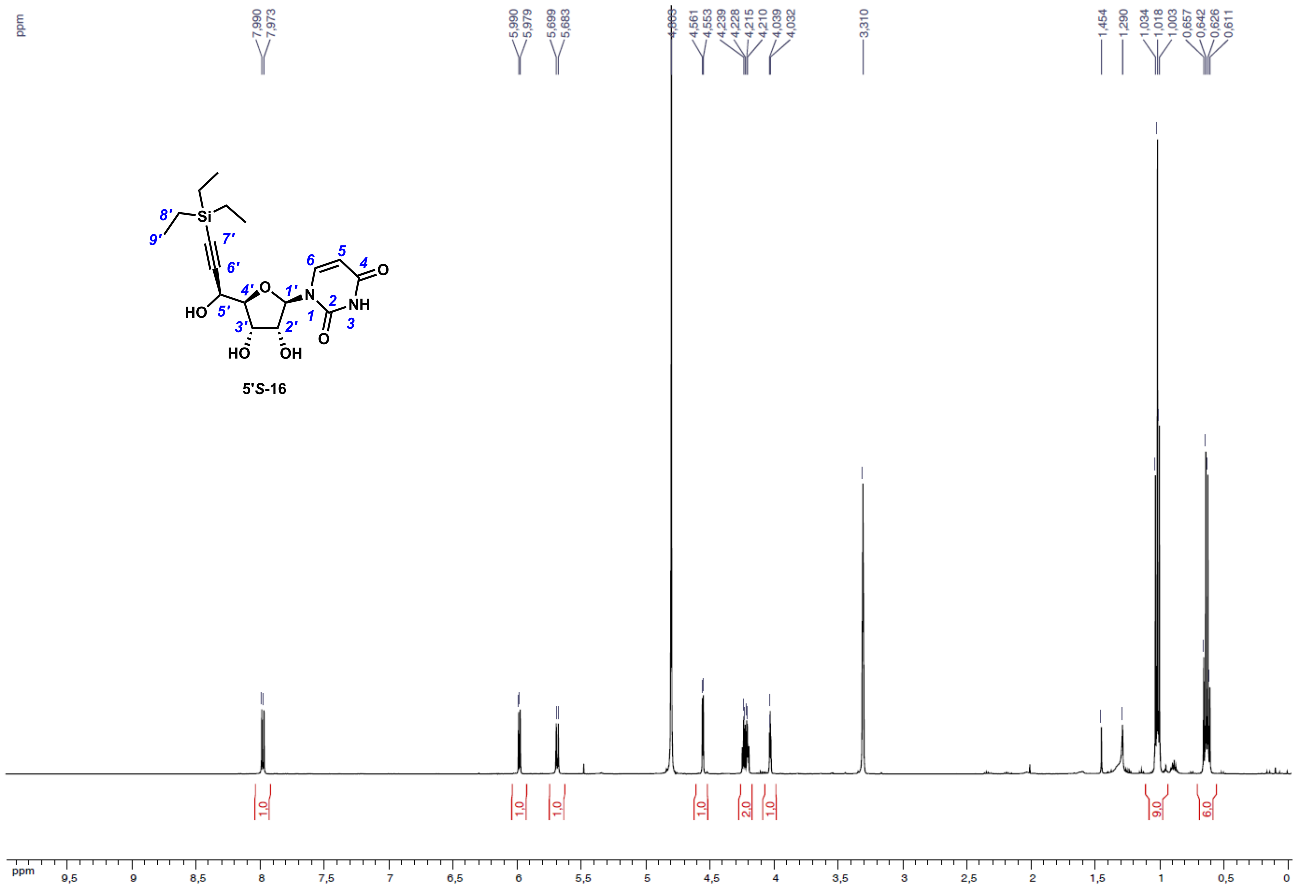

^{13}C NMR, 125 MHz (CD_3OD)

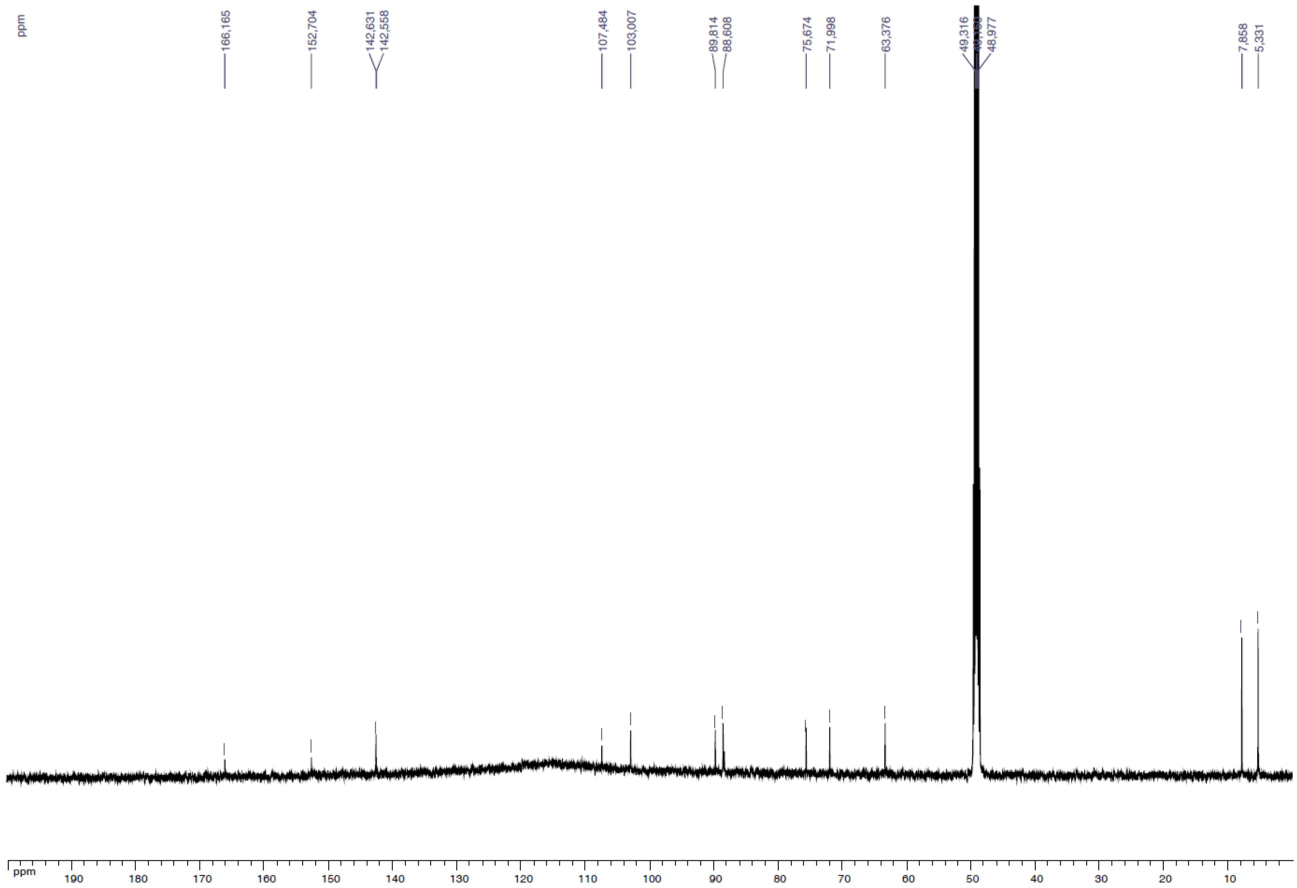

^{1}H NMR, 500 MHz (CD_3OD), compound **5’*R*-17**

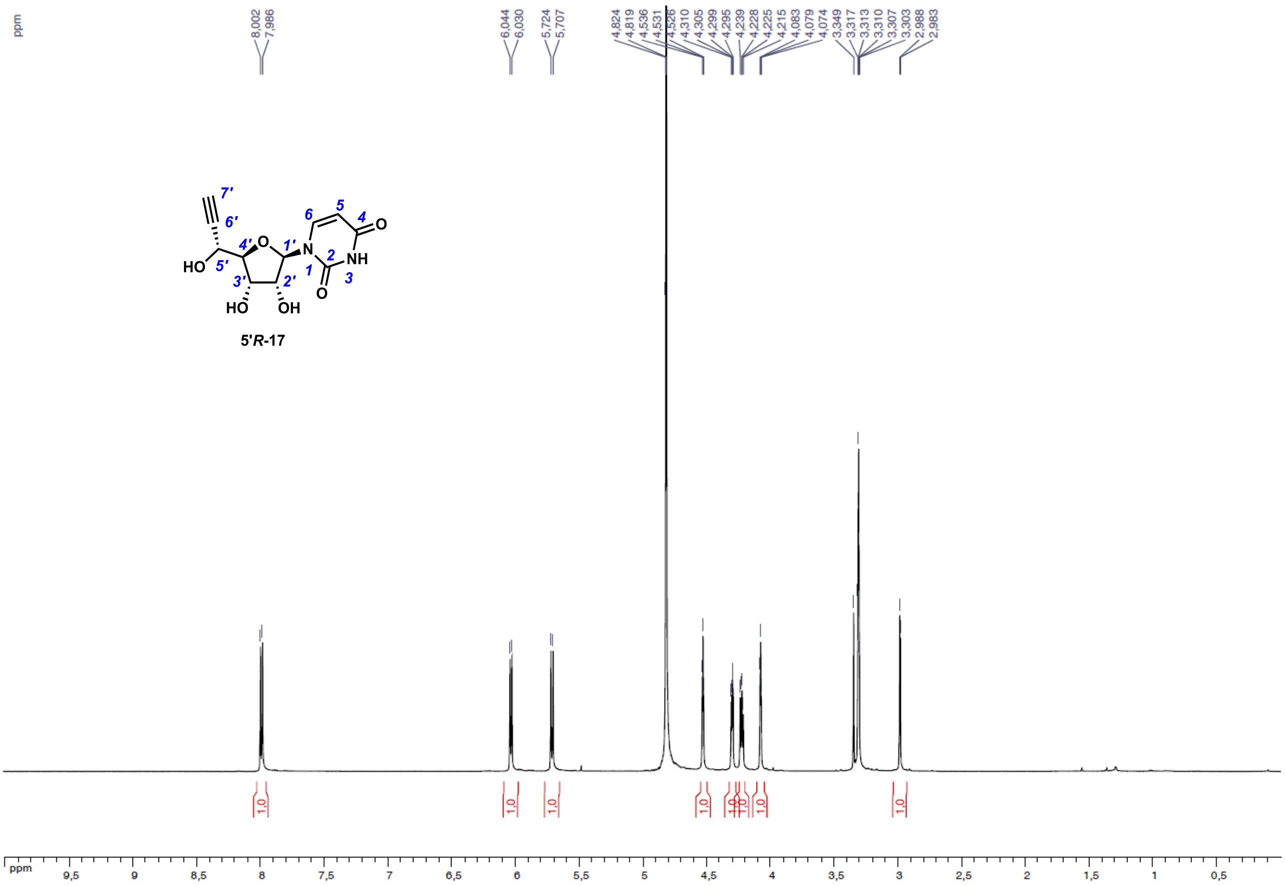

^{13}C NMR, 125 MHz (CD_3OD)

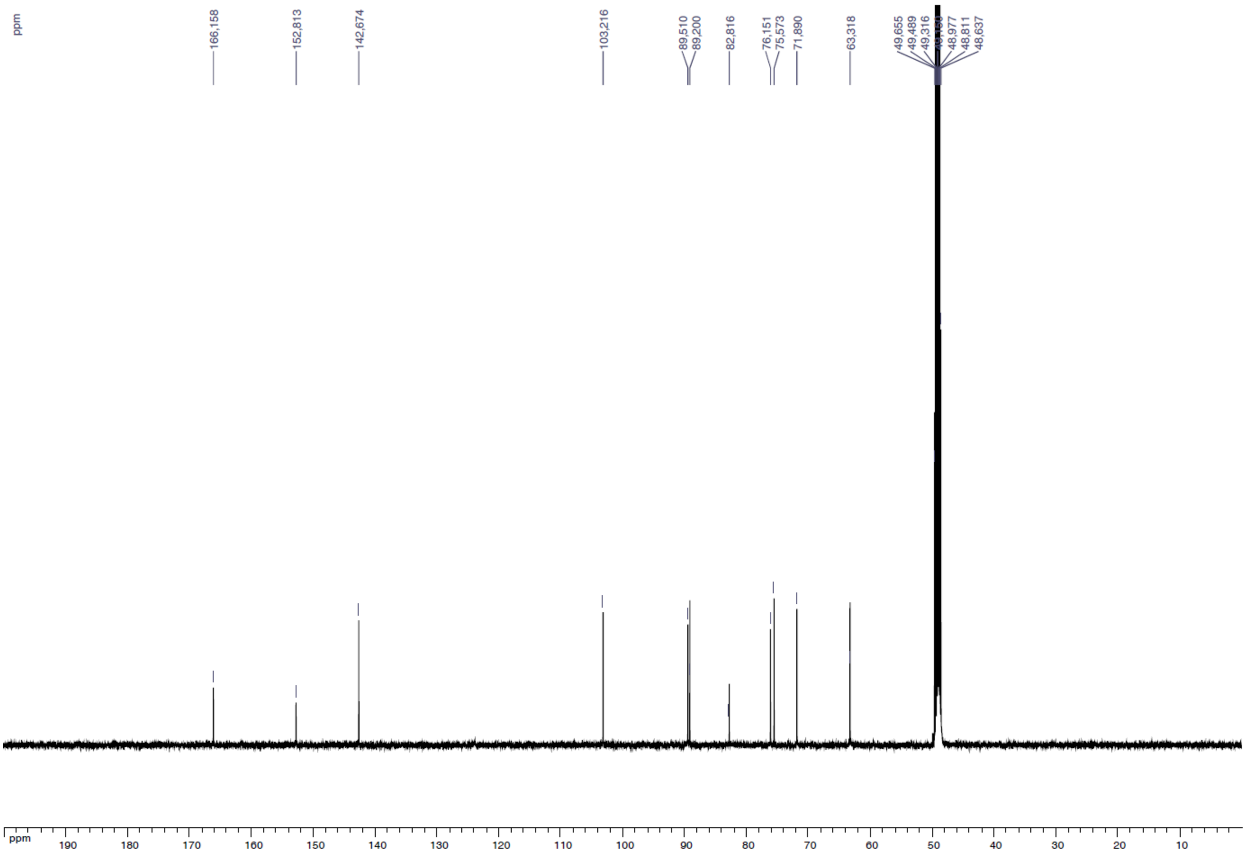

^{1}H NMR, 500 MHz (CD_3OD), compound **5’*S*-17**

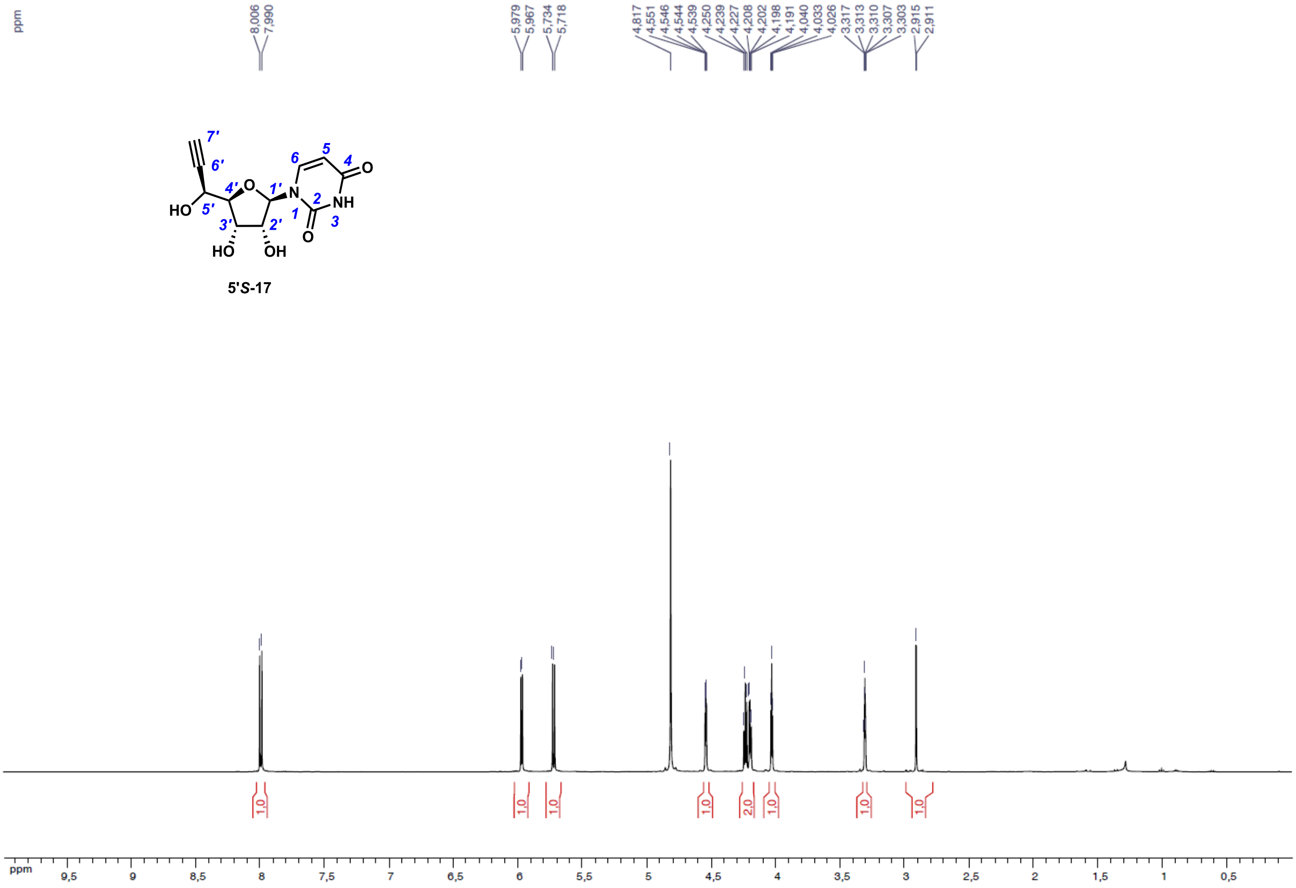

^{13}C NMR, 125 MHz (CD_3OD)

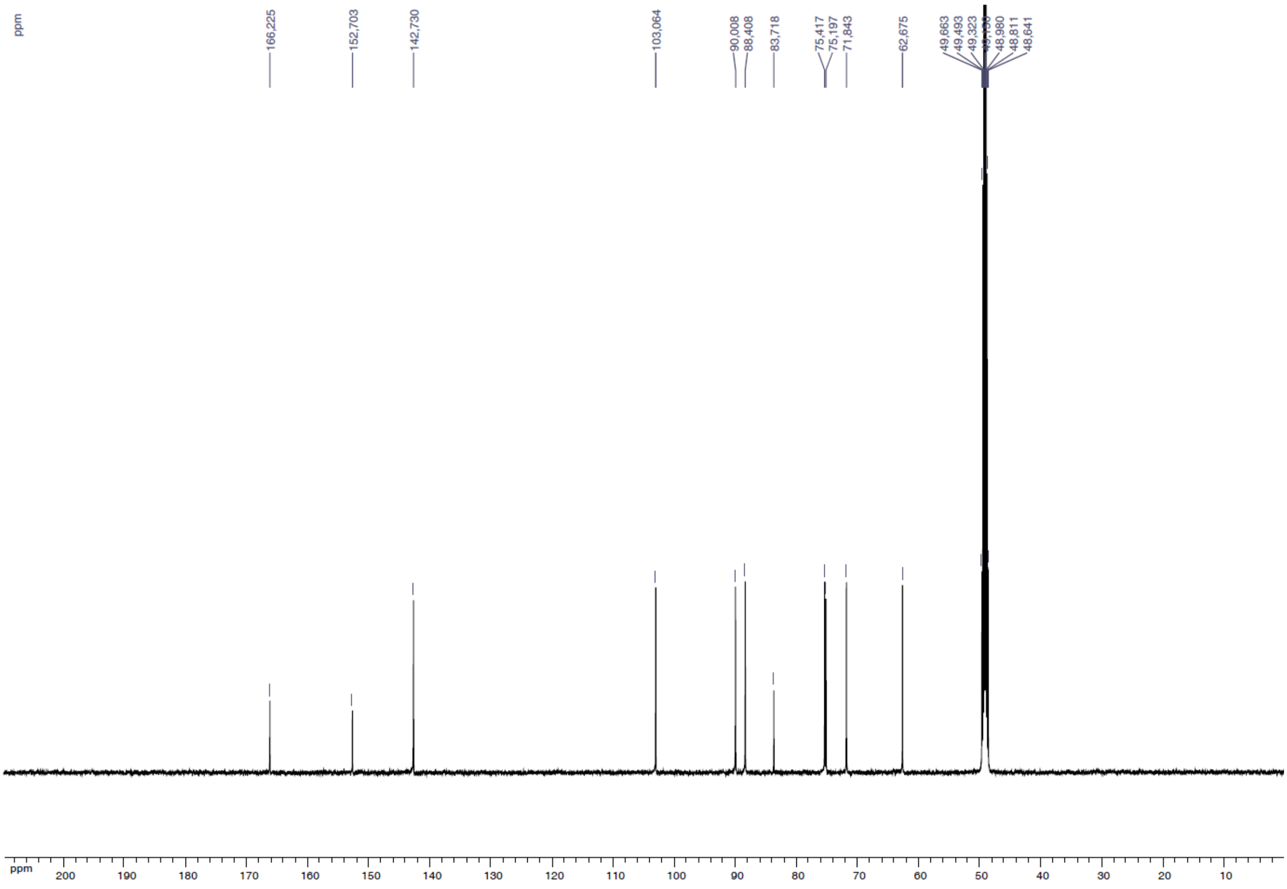

NMR spectrum of the crude for entry 1 (-50 °C)

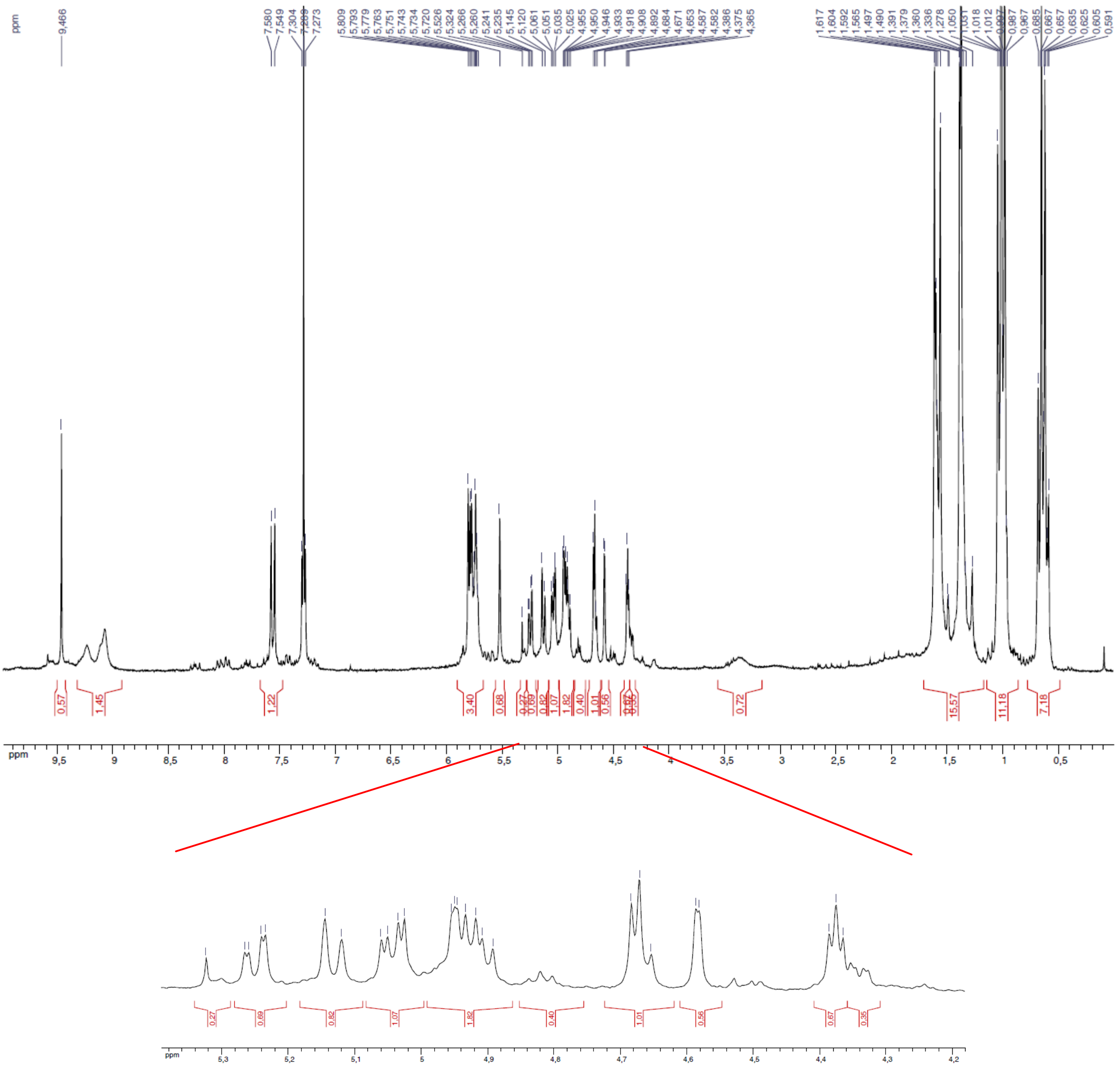

NMR spectrum of the crude for entry 2 (-78 °C)

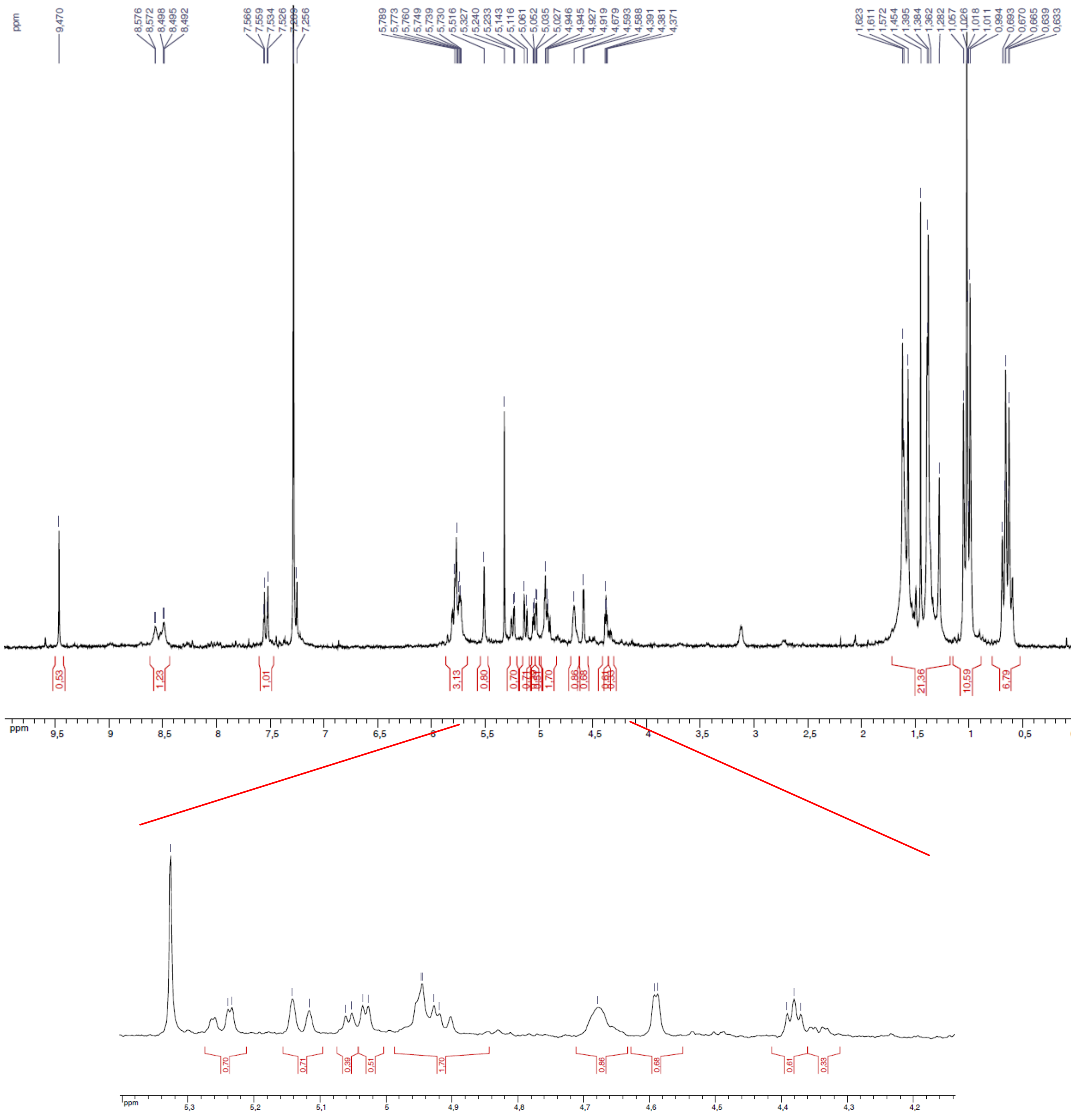

NMR spectrum of the crude for entry 3 (-15 °C)

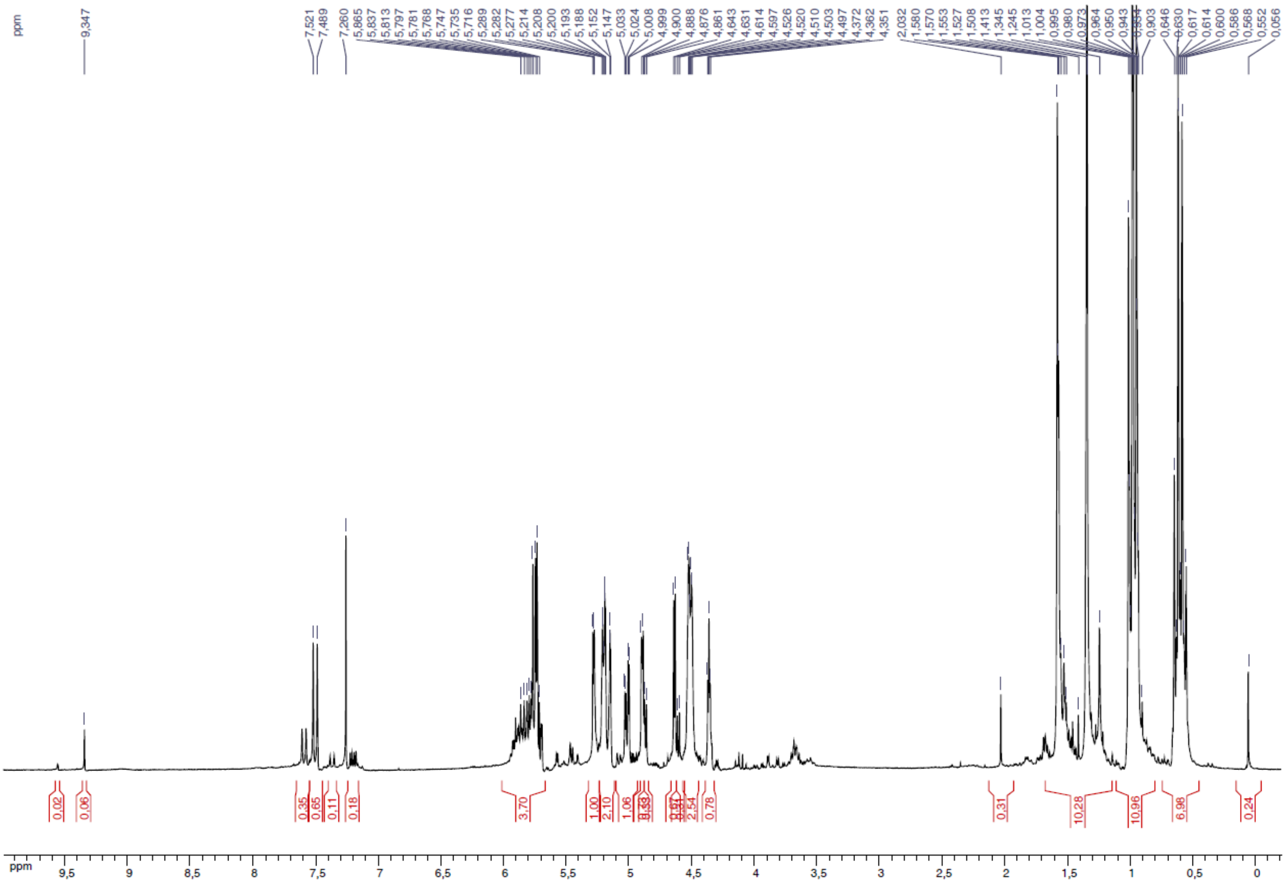

NMR spectrum of the crude for entry 4 (-15 °C)

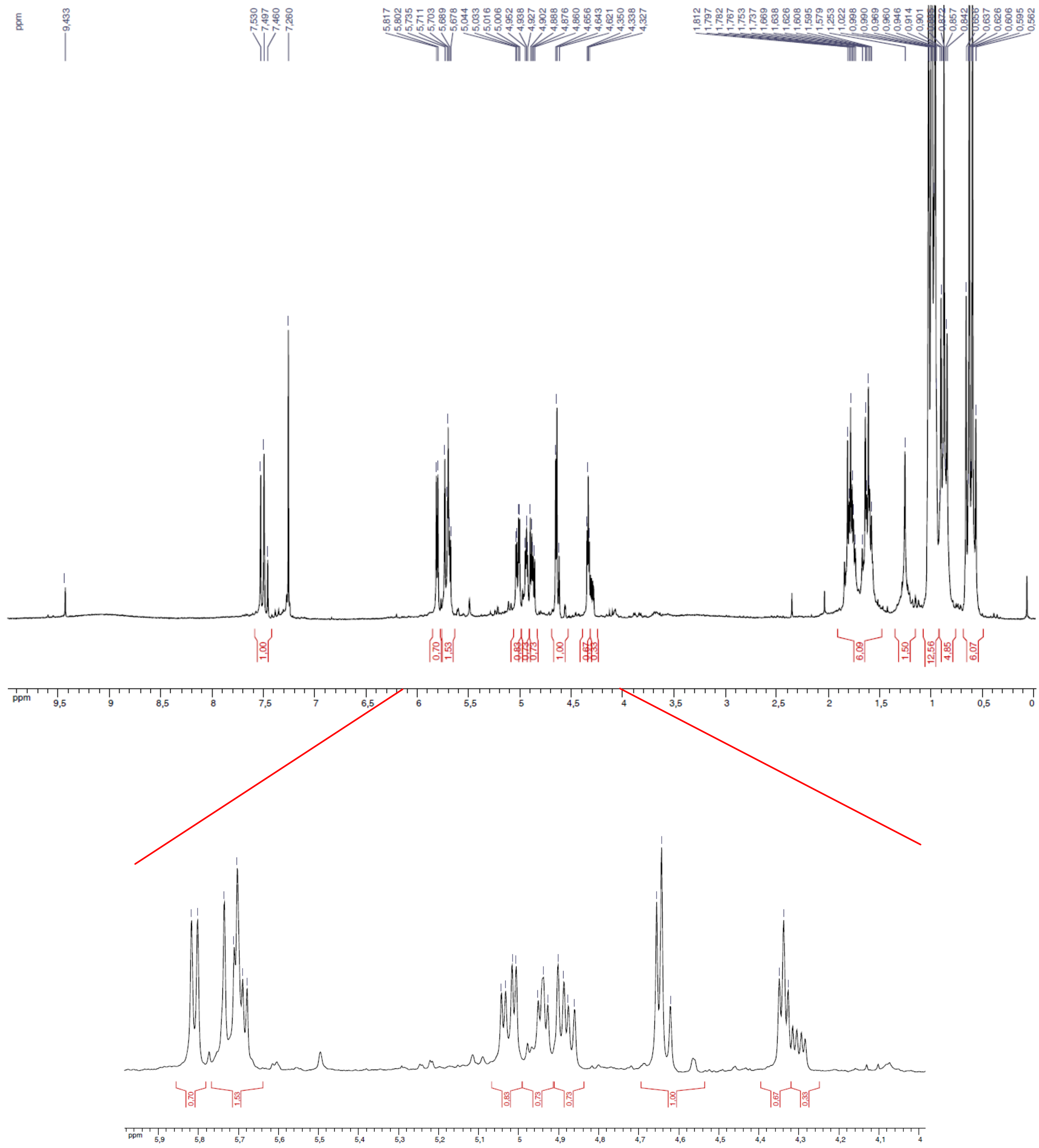

NMR spectrum of the crude for entry 5 (-15 °C)

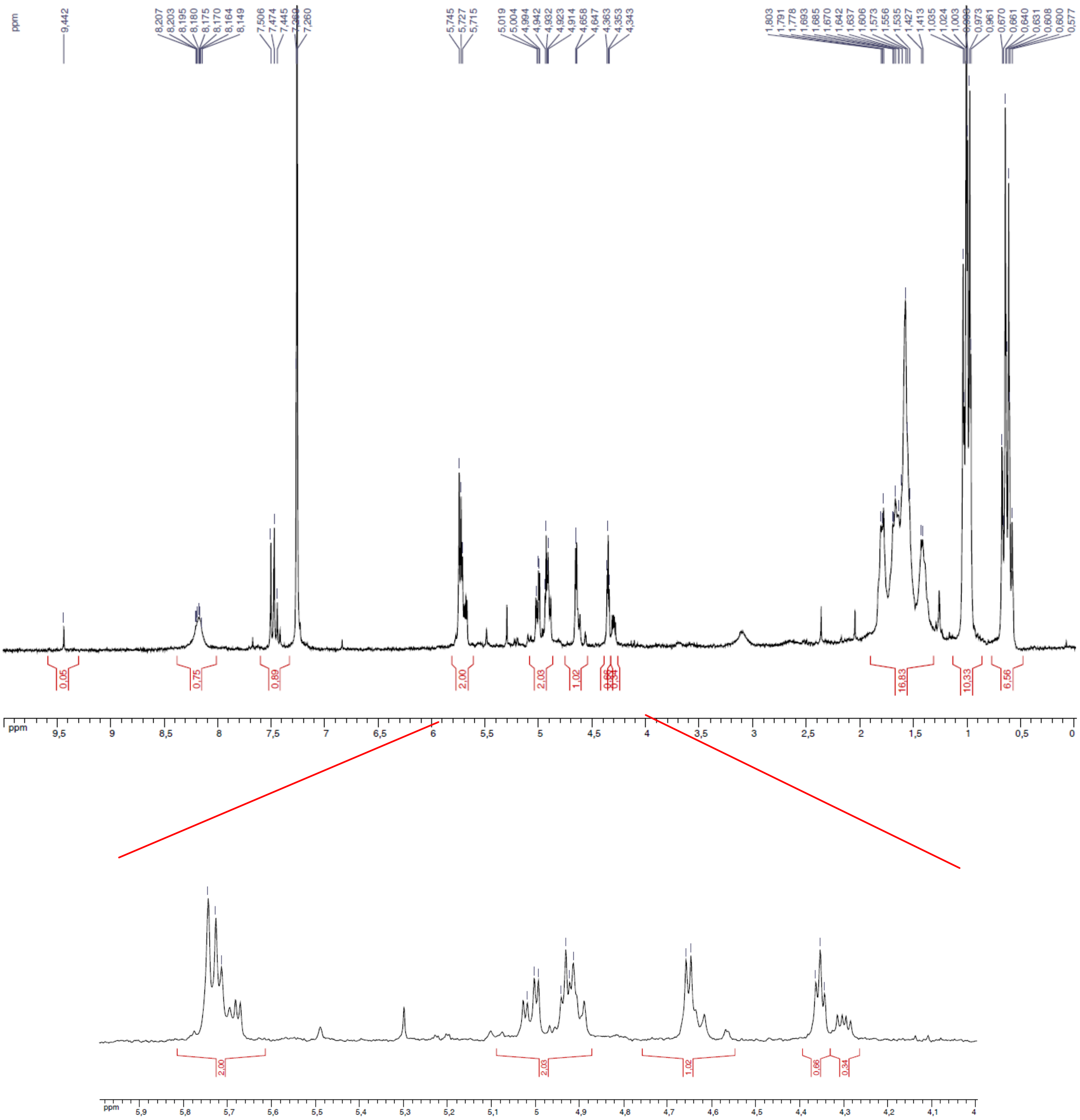

HPLC and ^{1}H NMR of the crude mixture for the 5'*R*/5'*S* determination

HPLC conditions were optimized on a 2/1 mixture 5'*S*/5'*R* of **14ab** (last fraction of a purification enriched in the minor diastereomer)

HPLC (CH_3CN / H_2O 60 / 40 to 0 / 100, 1.0 mL min^{-1}, 254 nm) ; t_R= 24.29 min, t_R= 25.56 min

Area % Report

Data File: D:\EZChrom Elite\Enterprise\Projects\Aurélie\Data\MF 652-5.dat
Method: D:\EZChrom Elite\Enterprise\Projects\Aurélie\Method\RBO.met
Acquired: 13/04/2016 14:47:37
Printed: 13/04/2016 15:24:49

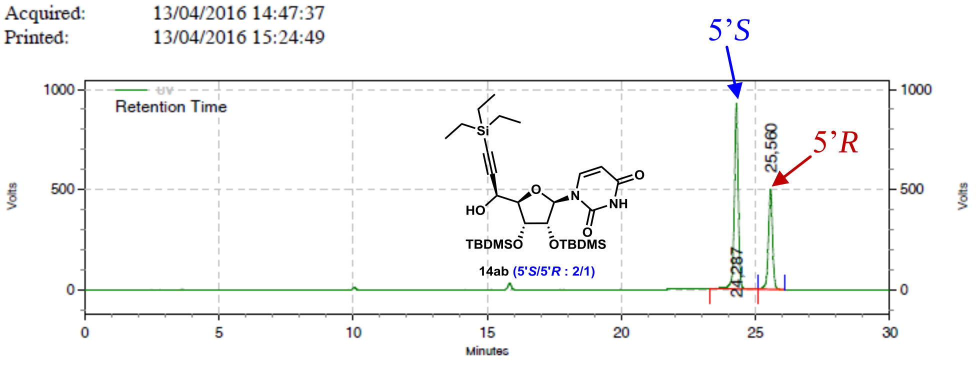

UV Results

Retention Time	Area	Area %	Height	Height %
24,287	36997826	63,44	3699455	64,98
25,560	21324477	36,56	1994042	35,02

HPLC and NMR spectra of the crude for entry 6 (- 15 °C)

HPLC (CH_3CN / H_2O 60 / 40 to 0 / 100, 1.0 mL min^{-1}, 254 nm) ; t_R= 29.60 min, t_R= 31.93 min

Area % Report

Data File: D:\EZChrom Elite\Enterprise\Projects\Aurélie\Data\rbo-tbs--15.dat
Method: D:\EZChrom Elite\Enterprise\Projects\Aurélie\Method\RBO.met
Acquired: 11/05/2016 12:52:45
Printed: 11/05/2016 16:49:52

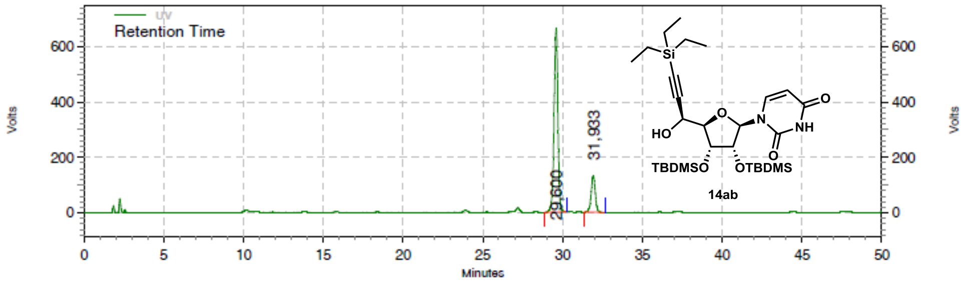

UV Results

Retention Time	Area	Area %	Height	Height %
29,600	44083635	82,38	2658218	83,52
31,933	9428089	17,62	524335	16,48

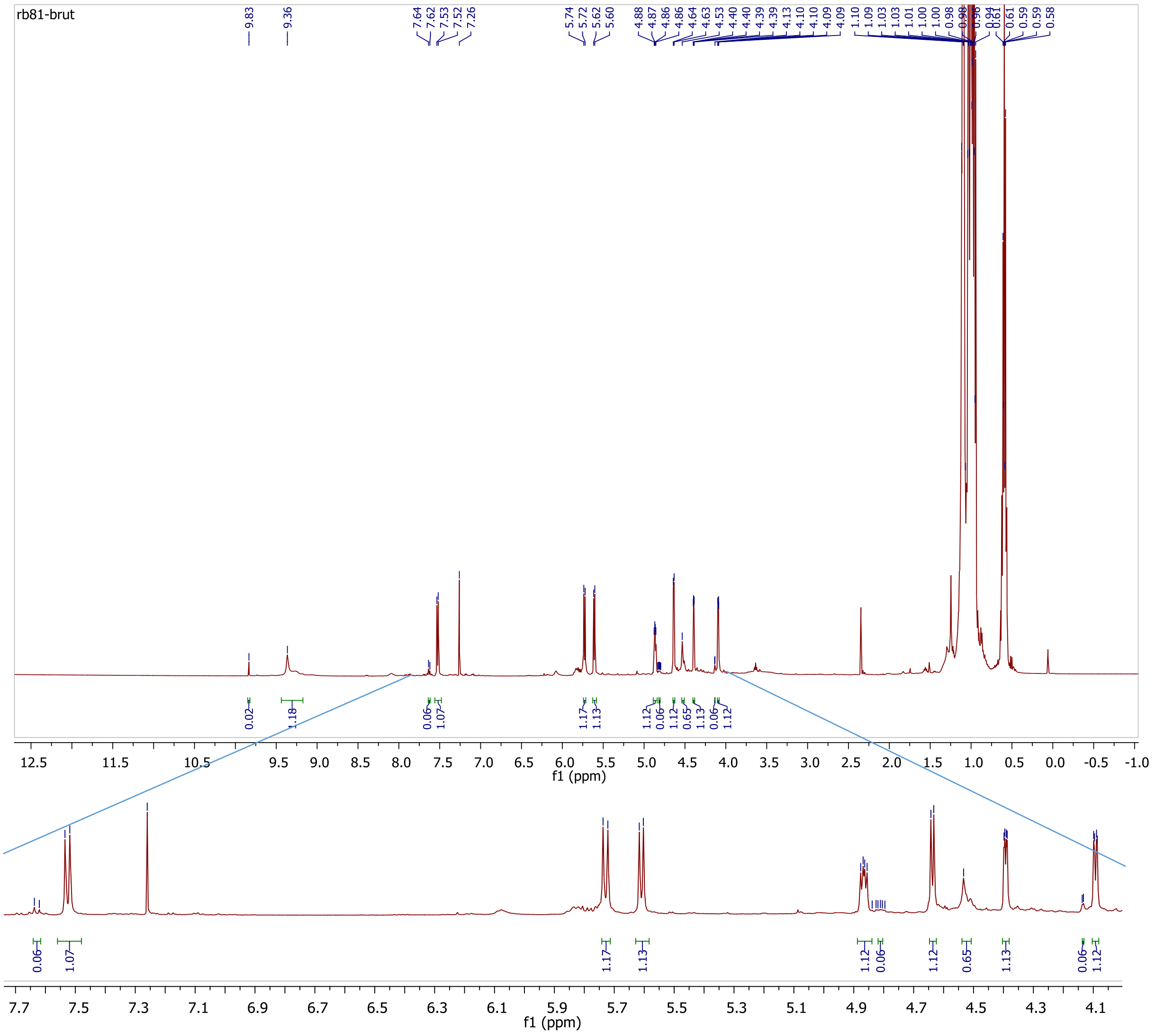

HPLC and NMR spectra of the crude for entry 7 (- 78 °C)

HPLC (CH_3CN / H_2O 80 /20 to 0 /100, 1.0 mL.min^{-1}, 254 nm) ; t_R= 20.04 min, t_R= 21.36 min

Area % Report

Data File:	D:\EZChrom Elite\Enterprise\Projects\Aurélie\Data\rbo-tbs--78.dat
Method:	D:\EZChrom Elite\Enterprise\Projects\Aurélie\Method\RBO.met
Acquired:	11/05/2016 14:23:18
Printed:	11/05/2016 16:53:08

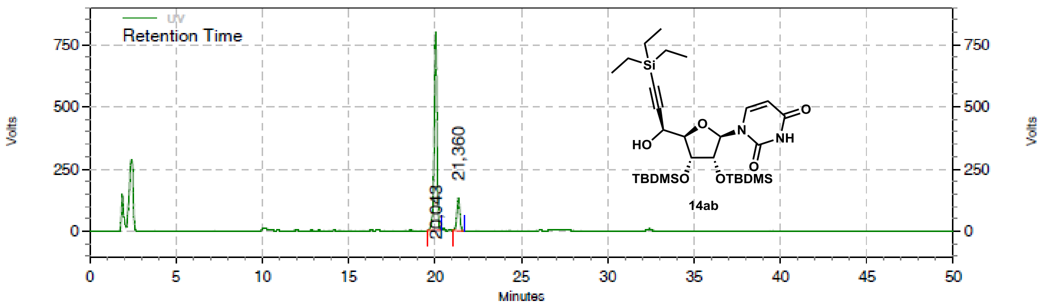

UV Results

Retention Time	Area	Area %	Height	Height %
20,043	33825746	85,09	3188798	85,85
21,360	5926668	14,91	525708	14,15

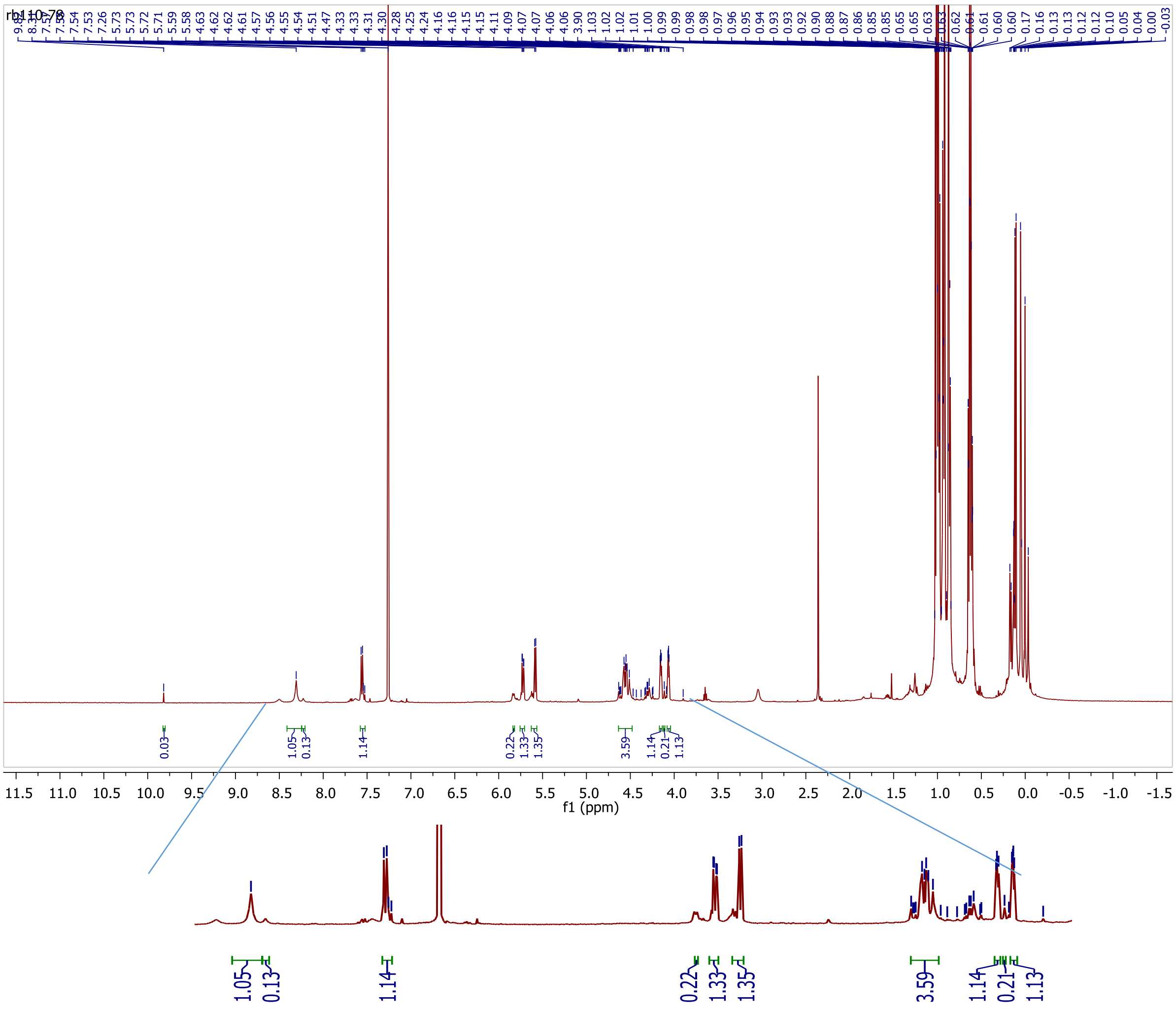

NMR spectrum of the crude for entry 8

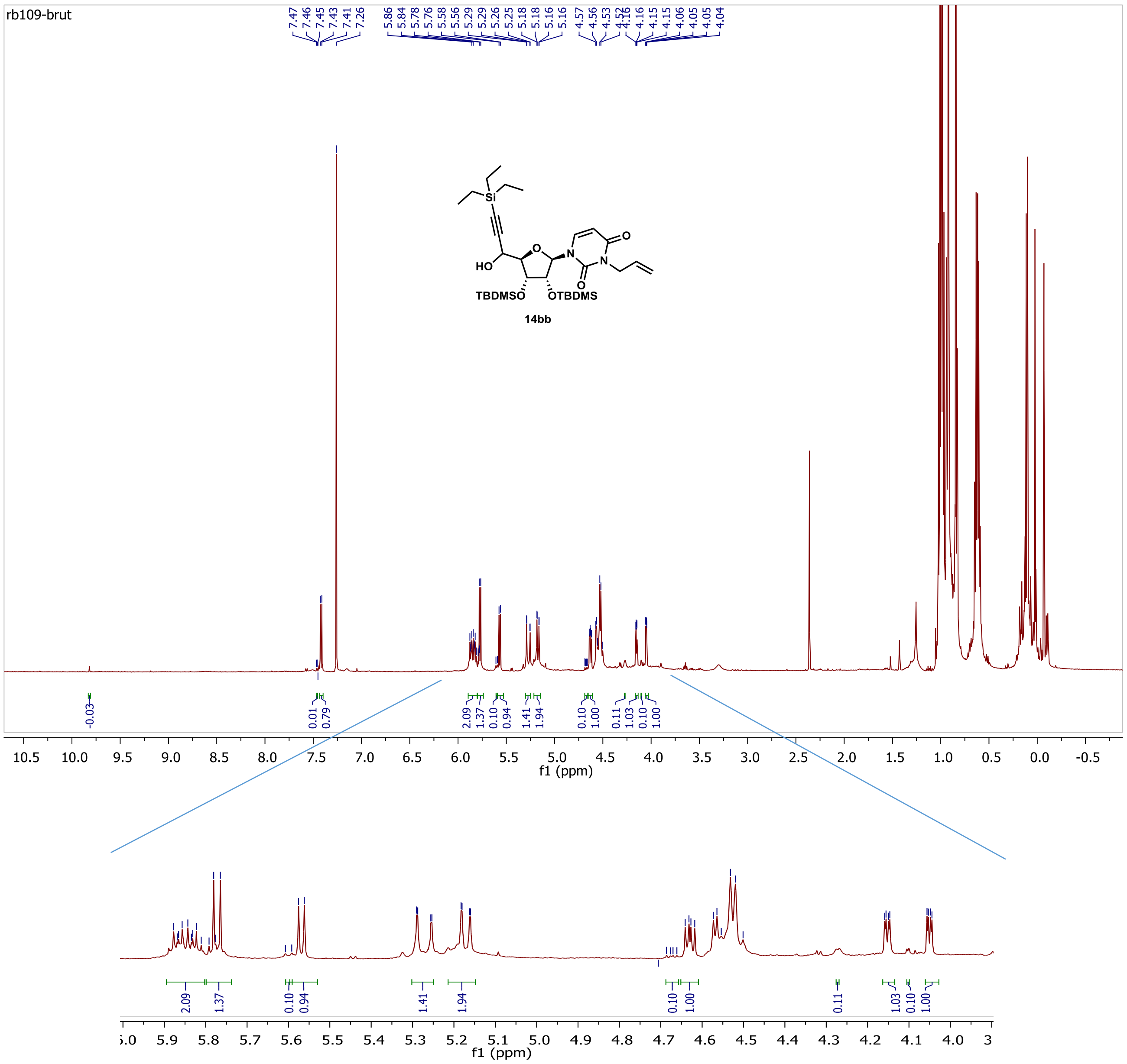

NMR spectrum of the crude for entry 9

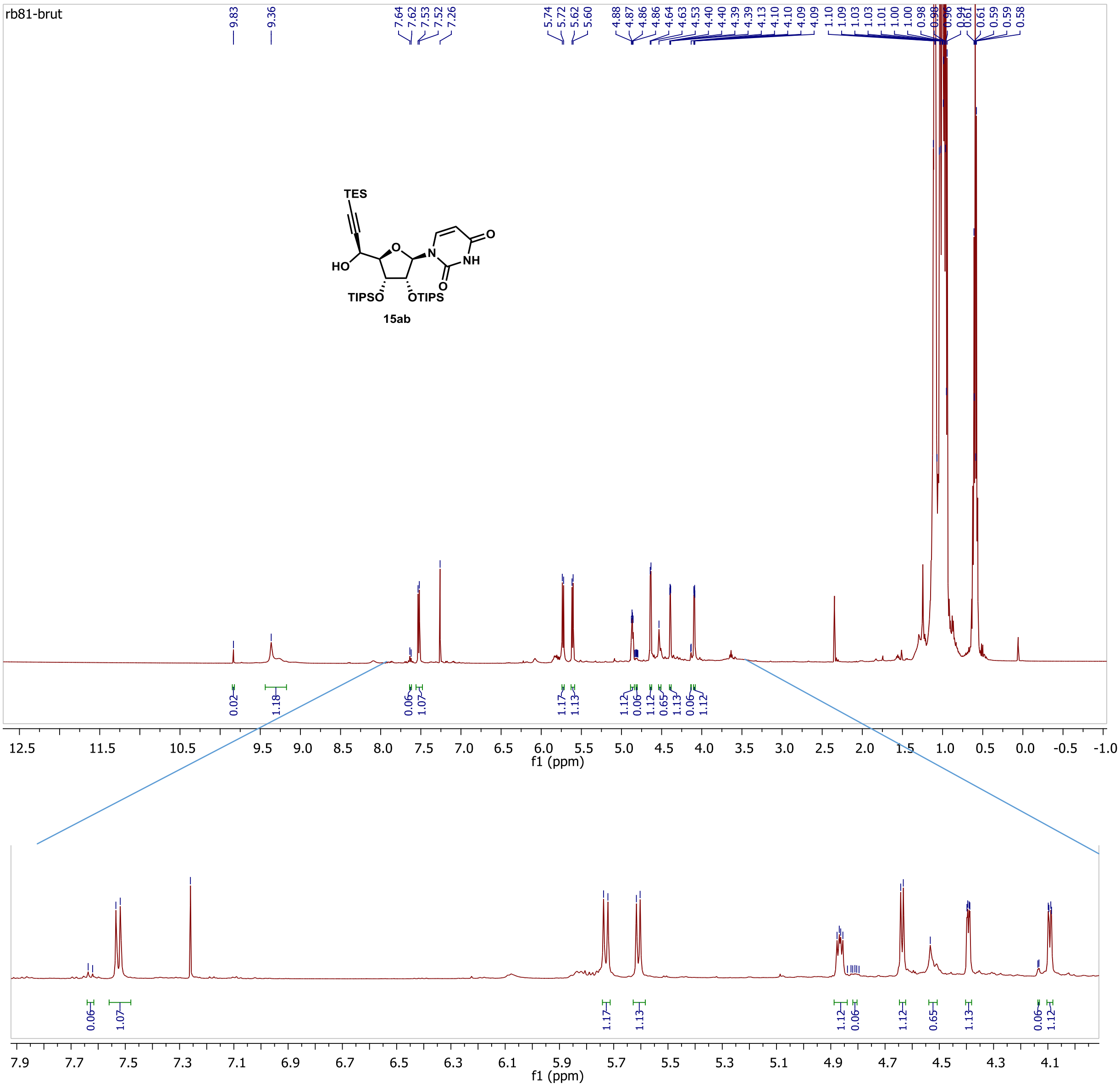

HPLC and NMR spectra of the crude for entry 10 (- 78 °C)

HPLC (CH_3CN / H_2O 60 /40 to 0 /100, 1.0 mL min^{-1}, 254 nm) ; t_R= 28.38 min, t_R= 32.94 min

Area % Report

Data File: D:\EZChrom Elite\Enterprise\Projects\Aurélie\Data\rbo-tes-3.dat
Method: D:\EZChrom Elite\Enterprise\Projects\Aurélie\Method\RBO.met
Acquired: 13/04/2016 17:22:03
Printed: 14/04/2016 09:44:51

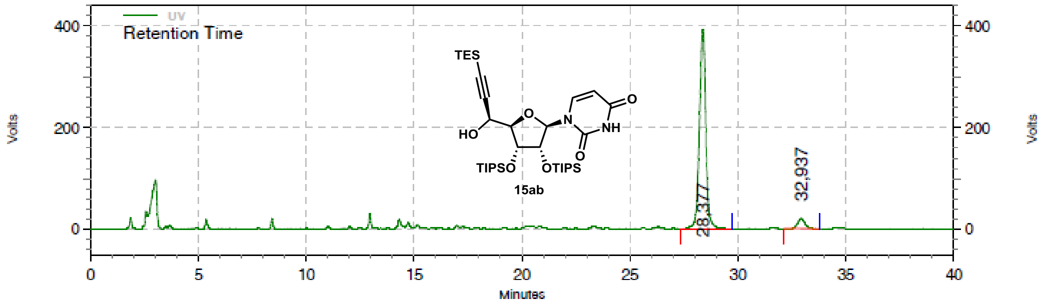

UV Results

Retention Time	Area	Area %	Height	Height %
28,377	31792512	94,33	1568029	95,20
32,937	1909706	5,67	79026	4,80

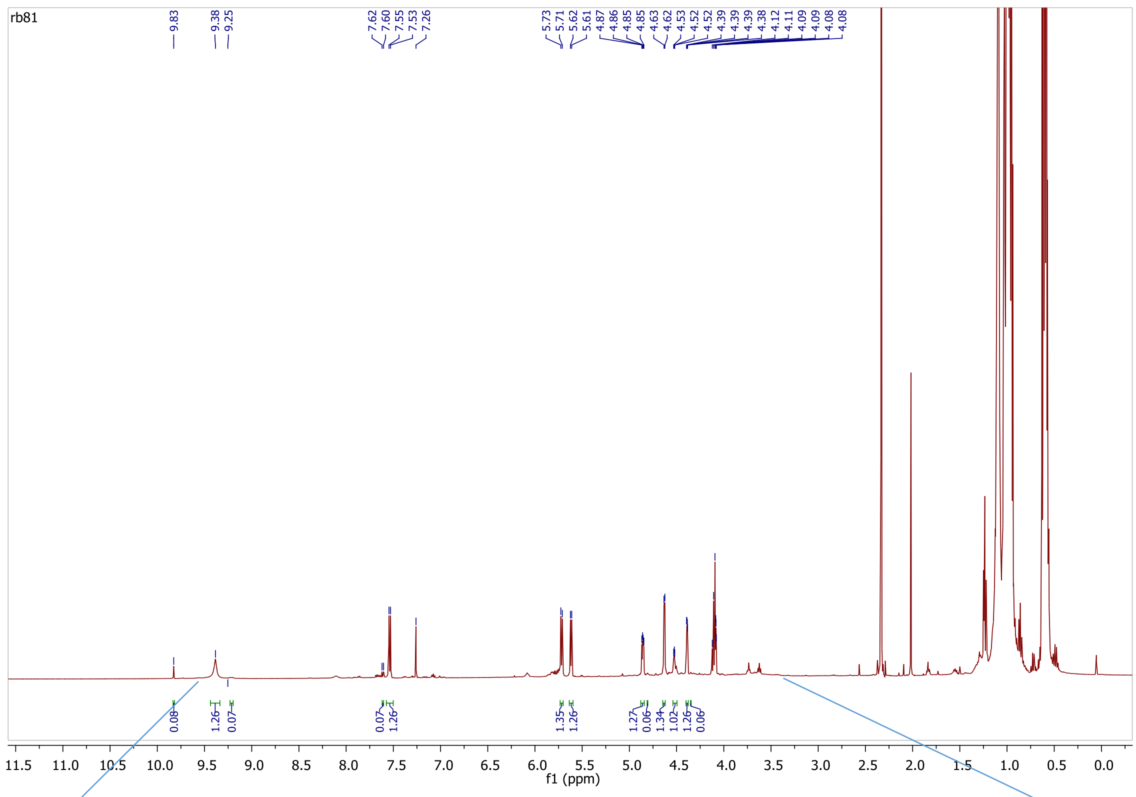

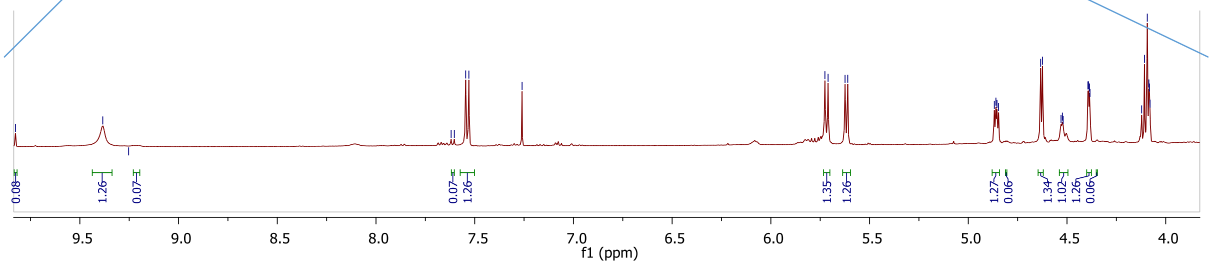

HPLC and NMR spectrum of the crude for entry 11

HPLC (CH_3CN / H_2O 60 /40 to 0 /100, 1.0 mL.min^{-1}, 254 nm) ; t_R= 20.79 min, t_R= 22.60 min

Area % Report

Data File: D:\EZChrom Elite\Enterprise\Projects\Aurélie\Data\rbo-tms-1.dat
Method: D:\EZChrom Elite\Enterprise\Projects\Aurélie\Method\RBO.met
Acquired: 14/04/2016 10:46:40
Printed: 14/04/2016 11:55:27

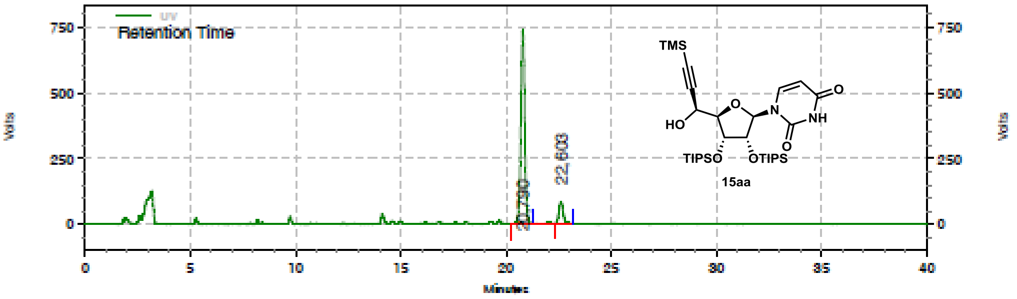

UV Results

Retention Time	Area	Area %	Height	Height %
20.790	37641651	88.15	2946012	89.75
22.603	5061014	11.85	336330	10.25

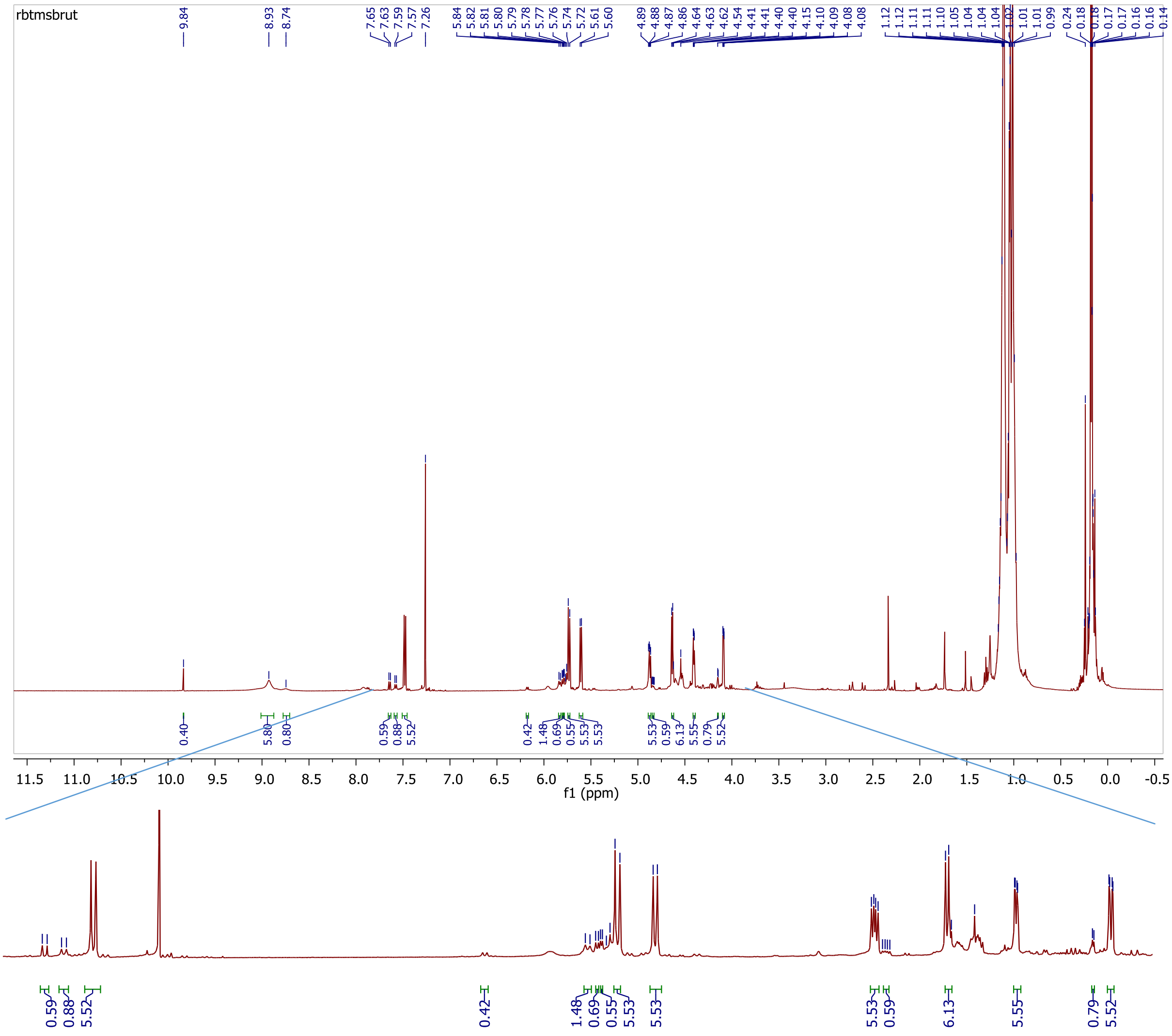

HPLC spectrum of a fraction enriched in the minor diastereomer

HPLC (CH_3CN / H_2O 60 / 40 to 0 / 100, 1.0 mL.min^{-1}, 254 nm) ; t_R= 28.08 min, t_R= 32.63 min

Area % Report

Data File: D:\EZChrom Elite\Enterprise\Projects\Aurélie\Data\rbo-tes-4.dat
Method: D:\EZChrom Elite\Enterprise\Projects\Aurélie\Method\untitled.met
Acquired: 14/04/2010 00:52:08
Printed: 13/05/2010 10:51:20

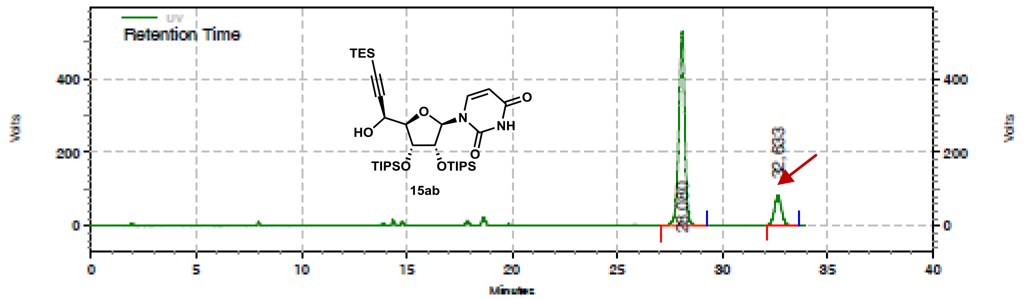

UV Results

Retention Time	Area	Area %	Height	Height %
28,080	42432780	84,71	2107745	86,87
32,633	7661250	15,29	318670	13,13

HPLC and NMR spectra of the crude for entry 12 (- 78 °C, 5 equiv. of Grignard reagent)

HPLC (CH_3CN / H_2O 60 / 40 to 0 / 100, 1.0 mL.min^{-1}, 254 nm) ; t_R= 38.29 min, t_R= 47.33 min

Area % Report

Data File: D:\EZChrom Elite\Enterprise\Projects\Aurélie\Data\rbo-2tips-2.dat
Method: D:\EZChrom Elite\Enterprise\Projects\Aurélie\Method\RBO.met
Acquired: 02/05/2016 16:04:56
Printed: 02/05/2016 17:10:34

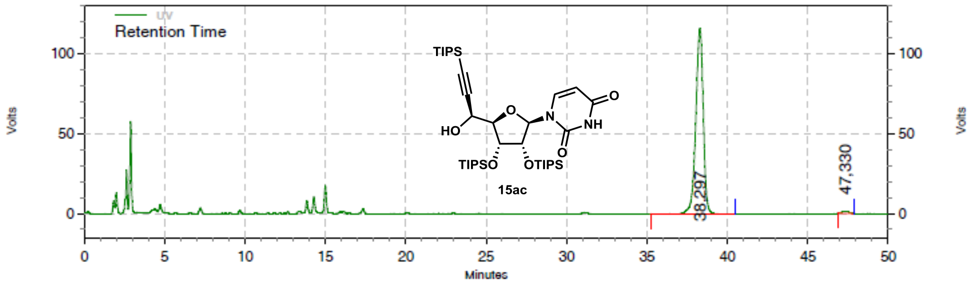

UV Results

Retention Time	Area	Area %	Height	Height %
38,297	15091725	98,81	465590	98,80
47,330	181142	1,19	5672	1,20

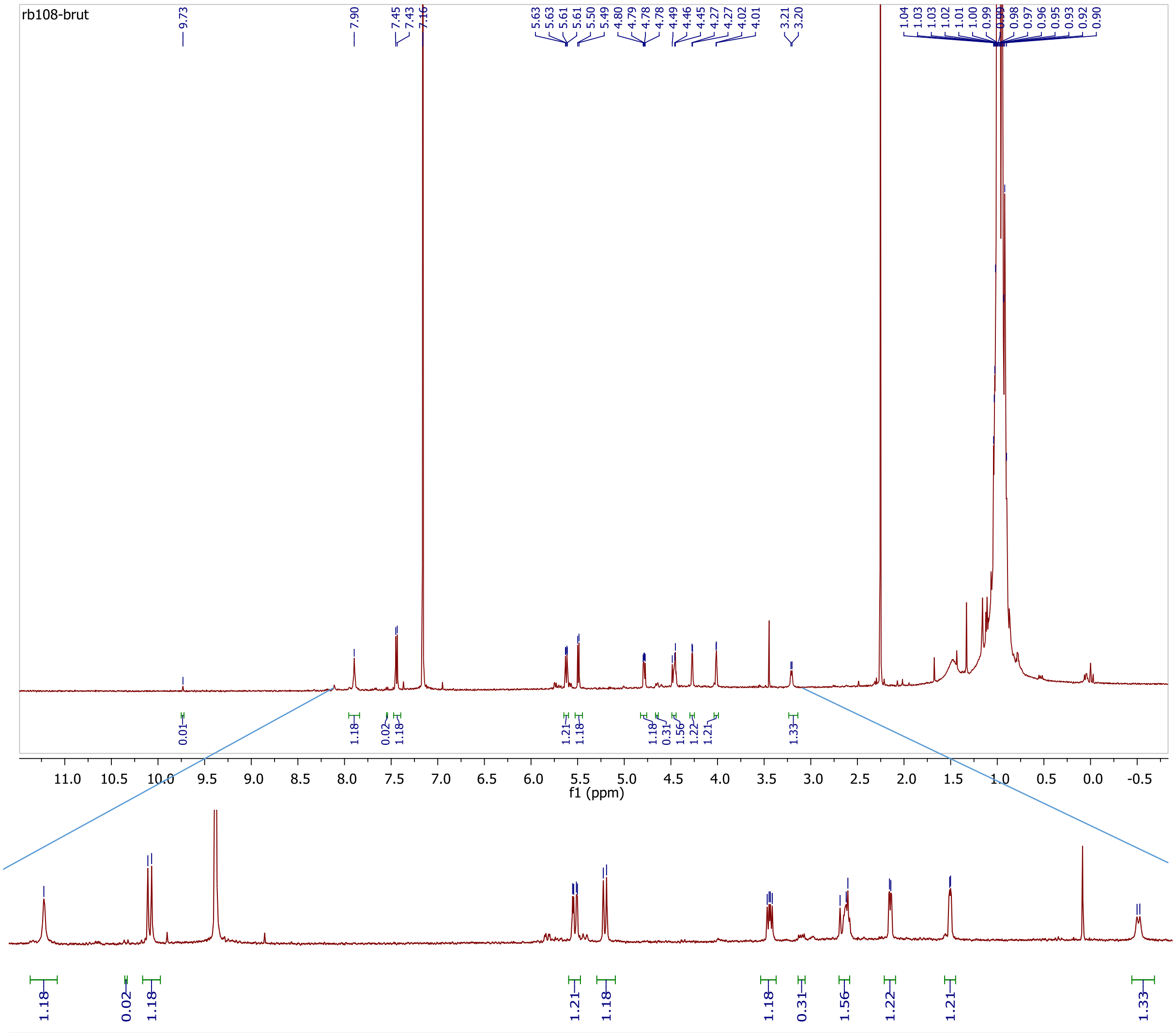

HPLC spectrum of the crude for entry 13 (- 15 °C, 5 equiv. of Grignard reagent)

HPLC (CH_3CN / H_2O 60 /40 to 0 /100, 1.0 mL.min^{-1}, 254 nm) ; t_R= 39.58 min, t_R= 48.21 min

Area % Report

Data File: D:\EZChrom Elite\Enterprise\Projects\Aurélie\Data\rbtips-tips-5eqi-15c.dat
Method: D:\EZChrom Elite\Enterprise\Projects\Aurélie\Method\RBO.met
Acquired: 21/06/2016 14:29:04
Printed: 21/06/2016 15:38:19

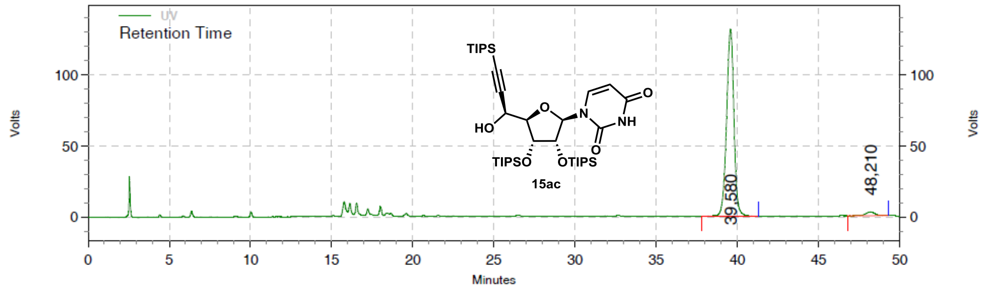

UV Results

Retention Time	Area	Area %	Height	Height %
39,580	16486718	97,48	525181	98,05
48,210	425558	2,52	10441	1,95

HPLC spectrum of the crude for entry 14 (- 15 °C, 2.5 equiv. of Grignard reagent)

HPLC (CH_3CN / H_2O 60 /40 to 0 /100, 1.0 mL.min^{-1}, 254 nm) ; t_R= 39.55 min, t_R= 48.16 min

Area % Report

Data File: D:\EZChrom Elite\Enterprise\Projects\Aurélie\Data\rbtips-tips-2.5eqi-15c.dat
Method: D:\EZChrom Elite\Enterprise\Projects\Aurélie\Method\RBO.met
Acquired: 21/06/2016 15:42:36
Printed: 21/06/2016 16:38:41

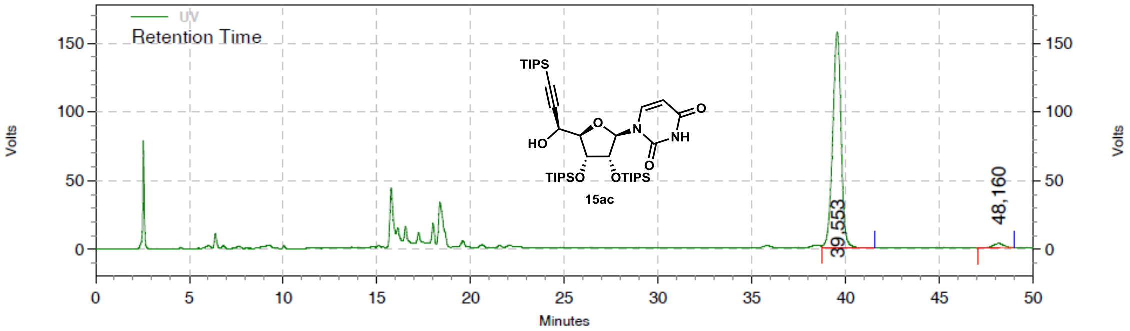

UV Results

Retention Time	Area	Area %	Height	Height %
39,553	19496086	97,51	627274	98,06
48,160	496880	2,49	12381	1,94

HPLC spectrum of the crude for entry 15

HPLC (CH_3CN / H_2O 60 / 40 to 0 / 100, 1.0.mL min^{-1}, 254 nm) ; t_R= 55.23 min, t_R= 68.48 min

Area % Report

Data File: D:\EZChrom Elite\Enterprise\Projects\Aurélie\Data\rbtips-tips-allyl -78-2.dat
Method: D:\EZChrom Elite\Enterprise\Projects\Aurélie\Method\RBO.met
Acquired: 29/06/2016 14:02:35
Printed: 29/06/2016 15:41:33

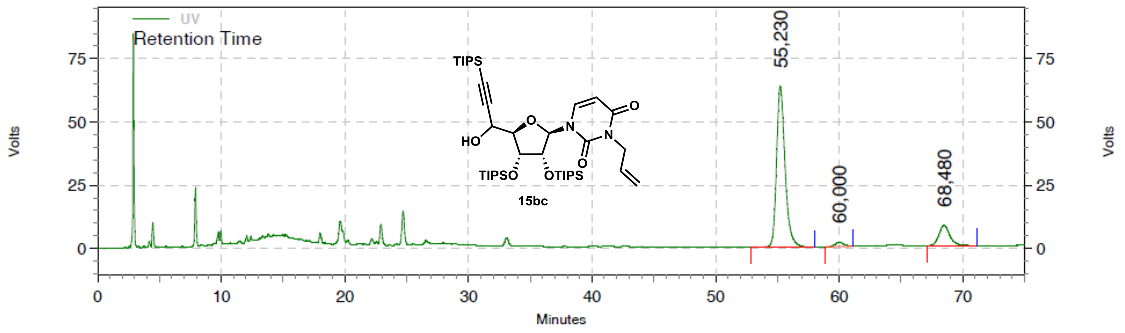

UV Results

Retention Time	Area	Area %	Height	Height %	
55,230	12103261	83,97	253196	86,73	88.83%
~~60,000~~	~~366316~~	~~2,54~~	~~6909~~	~~2,37~~	
68,480	1944803	13,49	31827	10,90	11.16%

Total 97.63

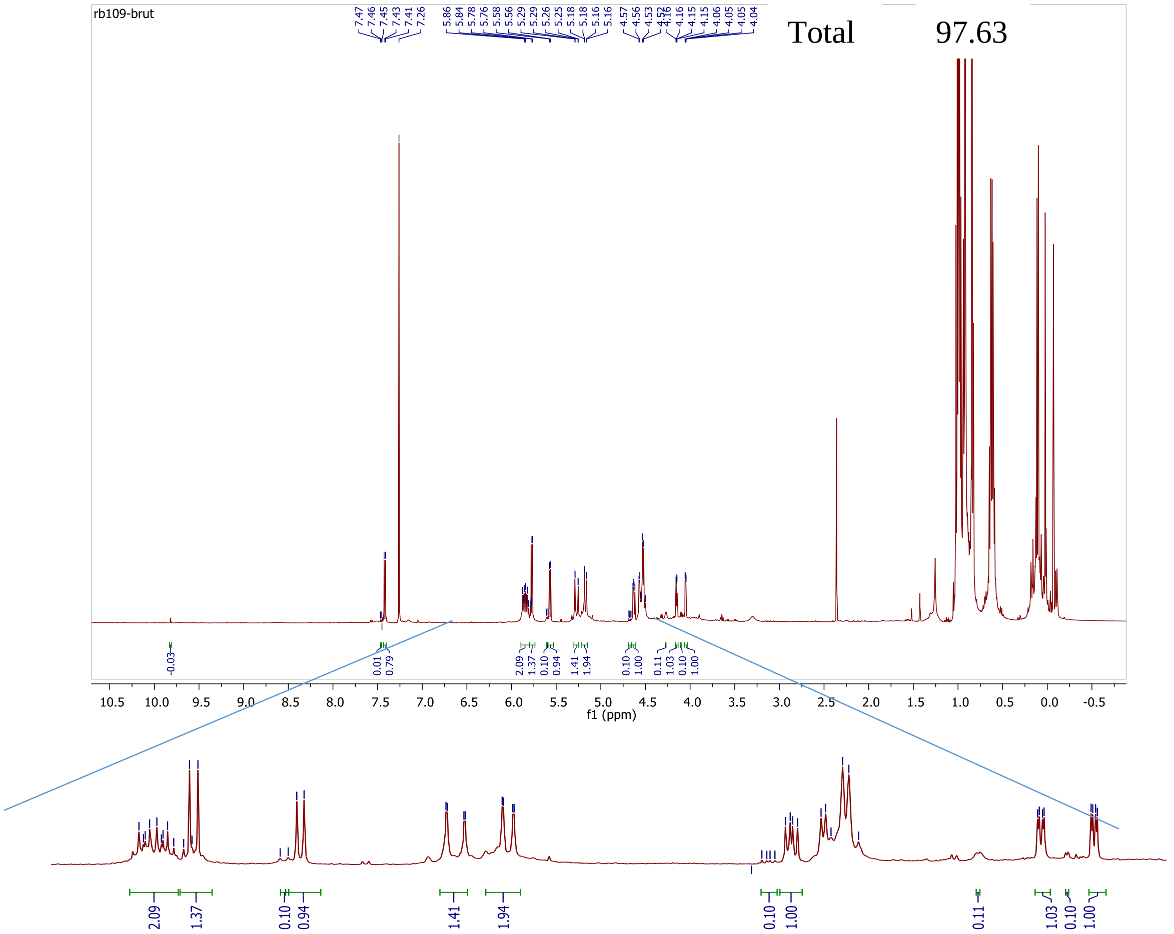

Supplement: File 2 — Copies of spectra for final compounds and NMR studies. [file Beilstein_J_Org_Chem-13-1533-s002.pdf]
